# Effects of a Guideline-Informed Clinical Decision Support System Intervention to Improve Colony-Stimulating Factor Prescribing: A Cluster Randomized Clinical Trial

**DOI:** 10.1001/jamanetworkopen.2022.38191

**Published:** 2022-10-24

**Authors:** Scott D. Ramsey, Aasthaa Bansal, Sean D. Sullivan, Gary H. Lyman, William E. Barlow, Kathryn B. Arnold, Kate Watabayashi, Ari Bell-Brown, Karma Kreizenbeck, Nguyet A. Le-Lindqwister, Carrie L. Dul, Ursa A. Brown-Glaberman, Robert J. Behrens, Victor Vogel, Nitya Alluri, Dawn L. Hershman

**Affiliations:** 1 Hutchinson Institute for Cancer Outcomes Research, Fred Hutchinson Cancer Research Center, Seattle, Washington; 2 The Comparative Health Outcomes, Policy, and Economics Institute, School of Pharmacy, University of Washington, Seattle; 3 School of Medicine, University of Washington, Seattle; 4 SWOG Statistics and Data Management Center, Seattle, Washington; 5 Illinois CancerCare–Peoria (Heartland Cancer Research National Cancer Institute Community Oncology Research Program), Peoria; 6 Ascension St John Hospital (Michigan Cancer Research Consortium National Cancer Institute Community Oncology Research Program), Detroit; 7 University of New Mexico Cancer Center (New Mexico Minority Underserved National Cancer Institute Community Oncology Research Program, Albuquerque; 8 Medical Oncology and Hematology Associates–Des Moines (Iowa-Wide Oncology Research Coalition National Cancer Institute Community Oncology Research Program), Des Moines; 9 Geisinger Medical Center (Geisinger Cancer Institute National Cancer Institute Community Oncology Research Program), Danville, Pennsylvania; 10 St Luke’s Cancer Institute–Boise (Pacific Cancer Research Consortium National Cancer Institute Community Oncology Research Program), Boise, Idaho; 11 Department of Medicine and Epidemiology, Columbia University, New York, New York

## Abstract

**Importance:**

Colony-stimulating factors are prescribed to patients undergoing chemotherapy to reduce the risk of febrile neutropenia. Research suggests that 55% to 95% of colony-stimulating factor prescribing is inconsistent with national guidelines.

**Objective:**

To examine whether a guideline-based standing order for primary prophylactic colony-stimulating factors improves use and reduces the incidence of febrile neutropenia.

**Design, Setting, and Participants:**

This cluster randomized clinical trial, the Trial Assessing CSF Prescribing Effectiveness and Risk (TrACER), involved 32 community oncology clinics in the US. Participants were adult patients with breast, colorectal, or non–small cell lung cancer initiating cancer therapy and enrolled between January 2016 and April 2020. Data analysis was performed from July to October 2021.

**Interventions:**

Sites were randomized 3:1 to implementation of a guideline-based primary prophylactic colony-stimulating factor standing order system or usual care. Automated orders were added for high-risk regimens, and an alert not to prescribe was included for low-risk regimens. Risk was based on National Comprehensive Cancer Network guidelines.

**Main Outcomes and Measures:**

The primary outcome was to find an increase in colony-stimulating factor use among high-risk patients from 40% to 75%, a reduction in use among low-risk patients from 17% to 7%, and a 50% reduction in febrile neutropenia rates in the intervention group. Mixed model logistic regression adjusted for correlation of outcomes within a clinic.

**Results:**

A total of 2946 patients (median [IQR] age, 59.0 [50.0-67.0] years; 2233 women [77.0%]; 2292 White [79.1%]) were enrolled; 2287 were randomized to the intervention, and 659 were randomized to usual care. Colony-stimulating factor use for patients receiving high-risk regimens was high and not significantly different between groups (847 of 950 patients [89.2%] in the intervention group vs 296 of 309 patients [95.8%] in the usual care group). Among high-risk patients, febrile neutropenia rates for the intervention (58 of 947 patients [6.1%]) and usual care (13 of 308 patients [4.2%]) groups were not significantly different. The febrile neutropenia rate for patients receiving high-risk regimens not receiving colony-stimulating factors was 14.9% (17 of 114 patients). Among the 585 patients receiving low-risk regimens, colony-stimulating factor use was low and did not differ between groups (29 of 457 patients [6.3%] in the intervention group vs 7 of 128 patients [5.5%] in the usual care group). Febrile neutropenia rates did not differ between usual care (1 of 127 patients [0.8%]) and the intervention (7 of 452 patients [1.5%]) groups.

**Conclusions and Relevance:**

In this cluster randomized clinical trial, implementation of a guideline-informed standing order did not affect colony-stimulating factor use or febrile neutropenia rates in high-risk and low-risk patients. Overall, use was generally appropriate for the level of risk. Standing order interventions do not appear to be necessary or effective in the setting of prophylactic colony-stimulating factor prescribing.

**Trial Registration:**

ClinicalTrials.gov Identifier: NCT02728596

## Key Points

**Question:**

Can a guideline-informed automated clinical decision support system improve use of primary prophylactic colony-stimulating factors for patients with cancer receiving myelosuppressive chemotherapy?

**Findings:**

In this cluster randomized clinical trial that included 32 community oncology practices and 2946 adult patients with cancer, primary prophylactic colony-stimulating factor use was high and did not differ significantly between groups (89.2% of patients in the intervention group vs 95.8% of patients in the usual care group). Febrile neutropenia rates were not significantly different between the intervention and usual care practices groups (6.1% vs 4.2%).

**Meaning:**

An automated standing order intervention did not improve primary prophylactic colony-stimulating factor use.

## Teaser

This cluster randomized clinical trial examines whether a guideline-based standing order for primary prophylactic colony-stimulating factors improves use of this treatment and reduces the incidence of febrile neutropenia among patients undergoing chemotherapy.

## Introduction

Unwanted variation in clinical practice has been a persistent and costly problem around the world. A common approach to address variation is evidence-based practice guidelines. Multiple studies^1,2,3,4,5,6,7,8,9^ show wide gaps between guideline recommendations and actual care. The disconnect between availability of evidence from practice guidelines and what is observed in practice has led researchers and care delivery systems to search for and test new methods aimed at their translation and implementation.

The widespread adoption of electronic health records (EHRs) has provided an opportunity to reduce variation. One widely used EHR-enabled approach is guideline-based, computerized clinical decision support systems, consisting of prompts or self-populating orders based on well-defined clinical criteria. Clinical examples of guideline-informed order sets include thromboprophylaxis prescribing and antibiotic prescribing for acute otitis media.

The potential benefits of these clinical decision support systems are particularly compelling in the case of primary prophylaxis colony-stimulating factors (PP-CSFs) for patients with cancer receiving myelosuppressive chemotherapy. PP-CSFs reduce the risk of febrile neutropenia (FN), an event associated with some chemotherapy regimens that carries a 10% to 20% mortality risk, as well as its severity and duration. For decades, clinical practice guidelines have recommended PP-CSF for patients receiving chemotherapy regimens with a high risk of FN and discouraged their use for patients receiving chemotherapy with a low risk of FN. Despite strong evidence and consistent guidelines published by multiple expert panels, prior studies^1,2,3,4,5,6,7,8,9^ have shown that between 55% and 95% of PP-CSF prescribing has been inconsistent with recommendations in both directions—that is, underuse of PP-CSF for chemotherapy regimens with high risk of FN and overuse of PP-CSF for low-risk chemotherapy regimens.^3,10,11,12,13,14^

Accordingly, we designed a stakeholder-informed, prospective, pragmatic (ie, conducted in a clinical practice setting, designed for generalizability),^15^ cluster randomized clinical trial (RCT), the Trial Assessing CSF Prescribing Effectiveness and Risk (TrACER),^16^ comparing guideline-informed, EHR-enabled, standing order sets for PP-CSF compared with usual care (UC) for patients with breast, lung, and colorectal cancer receiving initial myelosuppressive cancer-directed therapy. Our primary objective was to determine whether automated orders improved guideline adherence and reduced the incidence of FN compared with UC.

## Methods

### Trial Design and Setting

We conducted a pragmatic, cluster RCT within the SWOG Cancer Research Network and the National Cancer Institute Community Oncology Research Program (NCORP). NCORP is a network of researchers, public hospitals, physician practices, and other groups that provide health care services in US communities, organized into research bases and community sites. All aspects of the design, analysis, and reporting of the trial were informed by an external stakeholder advisory group.^17^

This study was approved by the Fred Hutchinson Cancer Center institutional review board and by the local institutional review boards at each participating study site. This study follows the Consolidated Standards of Reporting Trials (CONSORT) reporting guideline.

**Figure 1.**
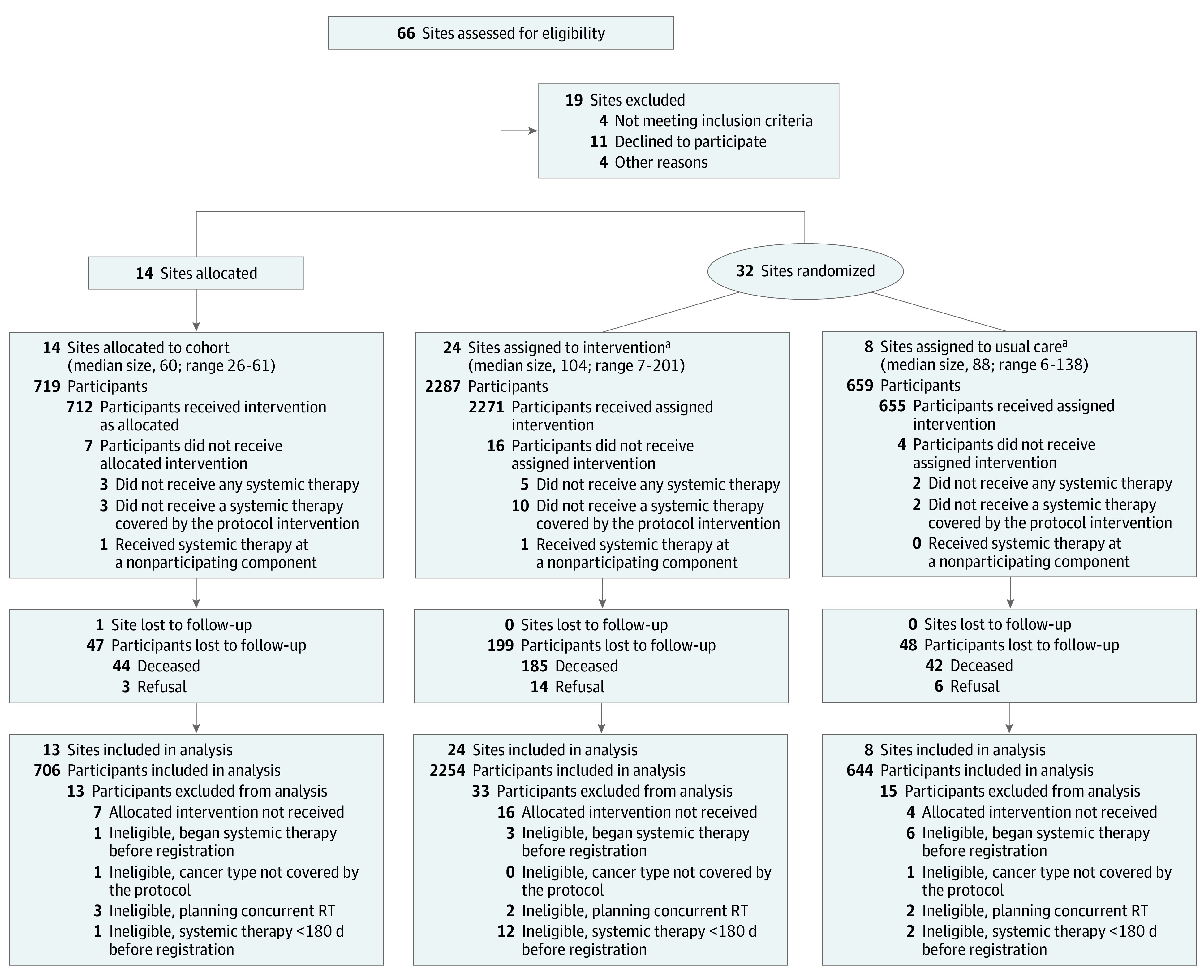
Trial Participation Diagram For the randomized sites, standing orders and system alerts recommended primary prophylactic colony-stimulating factor for all patients receiving chemotherapy regimens with a high risk of febrile neutropenia and no primary prophylactic colony-stimulating factor for patients receiving chemotherapy regimens with low risk of febrile neutropenia risk. Usual care had no modification to the existing chemotherapy order system at the site. Some patients who were lost to follow-up still had analyzable data that were submitted before they became lost to follow-up. These patients are included in the analysis section of the diagram. RT indicates radiation therapy. ^a^One additional site was allocated as a replacement for a previously randomized site that dropped out before receiving the intervention.

NCORP sites were eligible for the trial if they did not have standing PP-CSF order systems, and sites were randomized to either UC (ie, no changes to their order system) or intervention. Sites with an existing PP-CSF standing order system were assigned to a nonrandomized cohort group. Intervention sites modified their order system to include guideline-based alerts and automated PP-CSF orders for patients receiving chemotherapy regimens with high FN risk (hereafter, high-risk patients) and excluded PP-CSF orders for patients receiving regimens with low FN risk (hereafter, low-risk patients). The study included a second site randomization for patients receiving regimens with intermediate FN risk, for which PP-CSF guidelines are less clear. Results of this substudy will be described in a separate report. To maximize recruitment efficiency, sites with very low volume (<60 patients with breast, lung, and colorectal cancer seen annually) were ineligible. All clinical practices within each site were required to share the same chemotherapy ordering system (Figure 1).

### Patient Eligibility

Patients aged 18 years or older with a current diagnosis of breast, colorectal, or non–small cell lung (NSCL) cancer of any stage scheduled for first treatment with 1 of the study-allowed chemotherapy regimens were eligible. Further details on patient eligibility are available in Supplement 1. The order entry system included 77 National Comprehensive Cancer Network–recognized chemotherapy regimens and 40 biologic agents. Patients were enrolled before PP-CSF administration and provided written informed consent. The study opened to accrual in January 2016 and closed April 2020 after meeting the total accrual goal. Patient stipend or incentives were not provided. Study procedures and materials were approved by the institutional review board at each site.

### Guideline-Informed Standing Order Intervention

When clinicians entered chemotherapy orders, the order-entry intervention either autopopulated or did not populate PP-CSF orders according to the regimen’s FN risk. Clinicians could override standing orders at their discretion. Further details of the trial and intervention design are described in a separate publication.^16^ The order sets encompassed the spectrum of platforms used by the intervention clinics. In 5 clinics using paper-based orders, preprinted paper templates were incorporated into process workflows. Intervention ordering system modifications were embedded into the chemotherapy regimen order set, pilot tested, and reviewed with clinic practitioners in training sessions before patient enrollment.

### Outcomes

The primary outcomes were (1) PP-CSF use relative to recommendations for high-risk and low-risk chemotherapy regimens, with PP-CSF defined as the initiation of granulocyte CSFs during the first cycle of myelosuppressive systemic therapy, given 24 to 72 hours after cessation of systemic therapy; and (2) incidence of FN within 6 months of registration. FN was defined by the National Cancer Institute Common Terminology Criteria for Adverse Events version 4.0^18^: absolute neutrophil count less than 1000 cells/μL and a single temperature of greater than 38.3 °C (101 °F) or a sustained temperature of 38 °C (100.4 °F) or higher for more than 1 hour. Patients were observed until death or 12 months after registration.

Observational cohort study sites already had established PP-CSF automated order entry systems. Decisions on when to use or not use PP-CSF were at the discretion of the practice. The objective of the cohort study was to compare patterns of PP-CSF use and FN rates with those of the intervention and UC groups of the RCT.

### Data Collection

Clinical and demographic data including race and ethnicity were collected by clinical research staff from the medical record. Race and ethnicity were analyzed in this study because they may affect responses to chemotherapy. Additional patient demographics, comorbidities, and disease status were collected at baseline. Treatment received, dose modifications, and PP-CSF administration were collected at the end of the first cycle of systemic therapy. Antibiotic use was collected 1 month after registration. Incidence of FN was collected at the end of the first cycle and at 6 months.

### Trial Design

Randomization occurred at the site level, with sites randomly assigned to the intervention or UC groups using a 3:1 randomization scheme with stratification on clinic size and whether the clinic was classified as serving a predominantly underserved or minority population. Power calculations for the trial indicated 80% power to detect an increase in CSF use from 40% (UC) to 75% (intervention) for high-risk patients and a reduction in CSF use from 17% (UC) to 7% (intervention) for low-risk patients.^3^ There was 90% power to detect a 50% decrease in FN rate from 25% (UC) to 12.5% (intervention) for high-risk patients. To account for possible reduction in power due to correlation of patients among sites, power estimates accommodated an intraclass correlation coefficient (ICC) of 0.02 suggested by prior cluster randomized studies.^19^ The combined total accrual goal was 3600 eligible patients in the randomized and cohort groups.

### Statistical Analysis

Data analysis was performed from July to October 2021. Mixed effects multivariable logistic regression was used to compare UC with the intervention groups separately for low-risk and high-risk patients. The models included a random effect for each site assuming a normal distribution for the random effects. Two binary outcomes were tested separately: PP-CSF use and FN. A 2-sided α = .05 was used for each treatment comparison at each risk level and each outcome. Sensitivity analyses were conducted using population-averaged generalized estimating equations (GEEs) with exchangeable working correlation structure within clinics to estimate the ICC. The full statistical analysis plan is included in the protocol (Supplement 1). The statistical software suites used were SAS statistical software version 9.3 (packages SAS/BASE and SAS/STAT; SAS Institute) and R statistical software version 4.03 (extension sas7bdat; R Project for Statistical Computing).

#### Comparison of PP-CSF Use Between Intervention and UC Groups

We compared intervention vs UC with adjustment for important covariates (prognostic or potential confounders). Among high-risk patients, the primary analysis was a mixed effects logistic regression model with the binary outcome of whether PP-CSF was administered at the start of the first cycle of chemotherapy and a random effect for clinic. We adjusted for age group (<50, 50-59, 60-69, and ≥70 years), comorbidity, race, and Hispanic ethnicity. Since all high-risk patients had breast cancer, we did not adjust for sex and cancer type.

Among low-risk patients, the primary analysis was a mixed effects logistic regression model with the binary outcome of whether PP-CSF was administered at the start of the first cycle of chemotherapy and a random effect for clinic. The small number of events made it difficult to include many potential prognostic factors in the analysis; we adjusted for age group, sex, and cancer type.

#### Comparison of FN Rate Between Intervention and UC Groups

We compared the intervention vs UC groups by fitting mixed effects logistic regression models separately among high-risk and low-risk patients. The outcome was incidence of FN within 6 months of registration. We included a random effect for clinic. For high-risk patients, we adjusted for age; for low-risk patients, we adjusted for cancer type. Sensitivity analyses were conducted using population-averaged GEE with exchangeable working correlation structure within clinics to estimate the ICC.

## Results

A total of 3665 patients were registered at 45 community oncology clinics, with 2946 patients from 32 clinics included in the randomized groups (median [IQR] age, 59.0 [50.0-67.0] years; 2233 women [77.0%]; 2292 White [79.1%]); 2287 were randomized to the intervention, and 659 were randomized to UC) and 719 in the observational cohort (Table 1). Forty-eight randomized patients were found to be ineligible. Among the randomized patients, 1259 (43.4%) were high risk, 1053 (36.3%) were intermediate risk, and 586 (20.2%) were low risk.

**Table 1.** Patient Characteristics

Characteristic	Patients, No. (%)			
	Randomized cohort			Observational cohort (n = 719)
	Usual care (n = 659)	Intervention (n = 2287)	Total (N = 2946)	
Ineligible	15 (2.3)	33 (1.4)	48 (1.6)	12 (1.7)
Eligible and evaluable	644 (97.7)	2254 (98.6)	2898 (98.4)	707 (98.3)
Sites, No.	8	24	32	13
Patients per site, median (IQR)	86.0 (62.0-111.0)	101.1 (45.8-134.8)	96.0 (45.8-128.2)	59.0 (54.0-60.0)
Age, median (IQR), y	59.0 (50.0-66.0)	59.0 (49.0-67.0)	59.0 (50.0-67.0)	58.0 (49.0-67.0)
Sex				
Female	508 (78.9)	1725 (76.5)	2233 (77.0)	594 (84.0)
Male	136 (21.1)	529 (23.5)	665 (23.0)	113 (16.0)
Minority or underserved National Cancer Institute Community Oncology Research Program	237 (36.8)	576 (25.6)	813 (28.1)	0
Race				
Asian	15 (2.3)	69 (3.1)	84 (2.9)	10 (1.4)
Black	113 (7.5)	233 (10.3)	346 (11.9)	62 (8.8)
White	500 (77.6)	1792 (79.5)	2292 (79.1)	622 (88.0)
Other^a^	2 (0.3)	64 (2.8)	66 (2.3)	7 (1.0)
Unknown	14 (2.2)	96 (4.3)	110 (3.8)	6 (0.9)
Ethnicity				
Hispanic	26 (4.0)	337 (15.0)	363 (12.5)	14 (2.0)
Non-Hispanic	599 (93.0)	1858 (82.4)	2457 (84.8)	692 (97.9)
Unknown	19 (3.0)	59 (2.6)	78 (2.7)	1 (0.1)
Education				
Any high school	176 (27.3)	629 (27.9)	805 (27.8)	217 (30.7)
Any college or postgraduate	437 (67.9)	1299 (57.6)	1736 (59.9)	441 (62.4)
Unknown	31 (4.8)	326 (14.5)	357 (12.3)	49 (6.9)
Comorbidities, No.				
0	389 (60.5)	1249 (55.4)	1638 (56.9)	402 (57.3)
1-14	254 (39.5)	988 (43.8)	1242 (43.1)	299 (42.7)
Missing^b^	1	17	18	6
Cancer type				
Breast	397 (61.7)	1370 (60.8)	1767 (61.0)	508 (71.9)
Colorectal	181 (28.1)	613 (27.2)	794 (27.4)	136 (19.2)
Non–small cell lung cancer	66 (10.3)	271 (12.0)	337 (11.6)	63 (8.9)
Febrile neutropenia risk level				
High	309 (48.0)	950 (42.1)	1259 (43.4)	384 (54.3)
Intermediate^c^	207 (32.1)	846 (37.5)	1053 (36.3)	202 (28.6)
Low	128 (19.9)	458 (20.3)	586 (20.2)	121 (17.1)

^a^ Other includes Native American, Pacific Islander, and multiple races.

^b^ Missing data were not included in calculations of percentages.

^c^ Intermediate risk patients are not included in subsequent tables.

Demographic and baseline characteristics are shown in Table 1. Because sites are randomized rather than individuals, patient characteristics may be more heterogeneous between groups. The cancer type distribution was 61.0% breast (1767 patients), 27.4% colorectal (794 patients), and 11.6% NSCL (337 patients).

### PP-CSF Use

**Figure 2.**
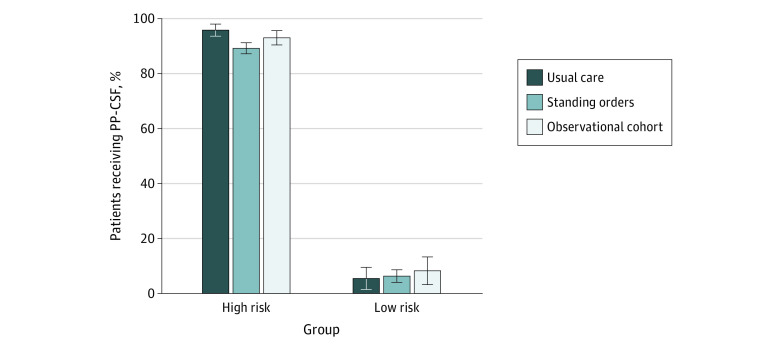
Primary Prophylactic Colony-Stimulating Factor (PP-CSF) Use by Group and Febrile Neutropenia (FN) Risk Level The bars indicate the percentage of patients using PP-CSF in each assigned treatment group by FN risk level. Error bars denote SEs. Differences in PP-CSF use between groups were not statistically significant after adjusting for covariates, in both the high-risk and low-risk groups.

Table 2 and Figure 2 describe PP-CSF use by group and FN risk level. Among high-risk patients, 95.8% (296 of 309 patients) in the UC group received PP-CSF compared with 89.2% (847 of 950 patients) in the intervention group. The adjusted odds ratio (OR) for intervention vs UC for high-risk patients was not significant (adjusted OR, 0.44; 95% CI, 0.12-1.57; *P* = .21) and was in the opposite direction from expected (Table 3). Unadjusted results were similar (data not shown), suggesting the comparison was little affected by the adjustment variables. Fitting a GEE model to directly estimate the ICC yielded intervention ORs comparable to those for the random effects models (eTable 1 in Supplement 2). When correcting for covariates, the ICC of prescribing within a clinic was 0.14, substantially higher than the pretrial estimate (ICC = 0.02). Prescribing rates for high-risk patients among intervention sites ranged from 49% to 100%, with a median (IQR) of 94% (86%-98%). Analyses suggest no difference between UC and intervention groups for high-risk patients. PP-CSF use among high-risk patients in the cohort group was 93.0% (357 of 384 patients).

**Table 2.** CSF Use and FN Rate by Treatment Group and FN Risk

Variable	Patients, No./total No. (%)			
	Randomized cohort			Observational cohort
	Usual care	Intervention	Total	
Clinics, No.	8	24	32	13
High risk^a^				
CSF use	296/309 (95.8)	847/950 (89.2)	1143/1259 (90.8)	357/384 (93.0)
Observed FN	13/308 (4.2)	58/947 (6.1)	71/1255 (5.7)	25/384 (6.5)
Low risk^a^				
CSF use	7/128 (5.5)	29/457 (6.3)	36/585 (6.1)	10/121 (8.3)
Observed FN	1/127 (0.8)	7/452 (1.5)	8/579 (1.4)	2/121 (1.7)

Abbreviations: CSF, colony-stimulating factor; FN, febrile neutropenia.

^a^ High risk refers to risk of FN greater than 20%; low risk refers to risk of FN less than 10%.

**Table 3.** Analysis 1: Colony-Stimulating Factor Use Among High-Risk and Low-Risk Patients

Variable	High-risk patients^a^		Low-risk patients^a^	
	Adjusted OR (95% CI)	*P* value	Adjusted OR (95% CI)	*P* value
Intervention vs usual care	0.44 (0.12-1.57)	.21	1.18 (0.44-3.20)	.74
Random effect variance	1.536	<.001	0.125	.29
Age group, y				
<50	1 [Reference]	.59	1 [Reference]	.11
50-59	0.84 (0.48-1.48)		0.40 (0.06-2.73)	
60-69	0.70 (0.40-1.22)		0.78 (0.15-4.11)	
≥70	1.01 (0.46-2.21)		1.58 (0.32-7.88)	
Male sex	NA^b^	NA^b^	0.43 (0.19-0.95)	.04
Cancer type				
Breast	All high-risk patients	NA	1 [Reference]	<.001
Colorectal	NA		0.50 (0.05-5.06)	
Non–small cell lung cancer	NA		11.35 (3.10-41.5)	
Comorbidity, >0 vs 0	0.76 (0.48-1.19)	.23	NA^c^	NA
Race				
Asian	0.32 (0.10-0.97)	.09	NA^c^	NA
Black	1.78 (0.85-3.74)		NA^c^	
White	1 [Reference]		NA^c^	
Other^d^	0.59 (0.19-1.81)		NA^c^	
Unknown	0.81 (0.25-2.58)		NA^c^	
Ethnicity				
Hispanic	1.07 (0.49-2.32)	.89	NA^c^	NA
Unknown	0.70 (0.14-3.56)		NA^c^	
Non-Hispanic	1 [Reference]		NA^c^	

Abbreviations: NA, not applicable; OR, odds ratio.

^a^ High-risk refers to more than 20% risk of febrile neutropenia; low-risk refers to less than 10% risk of febrile neutropenia.

^b^ Sex was not modeled because only 13 men had breast cancer.

^c^ Cancer type, comorbidity, race, and Hispanic ethnicity were not modeled because of the small number of events.

^d^ Other includes Native American, Pacific Islander, and multiple races.

PP-CSF use in low-risk patients was low within and across the randomized groups (36 of 585 patients [6.1%]) and the observational cohort (10 of 121 patients [8.3%]) (Table 2). PP-CSF use between intervention (29 of 457 patients [6.3%]) and UC (7 of 128 patients [5.5%]) groups did not differ. The adjusted OR for PP-CSF use in intervention vs UC was not statistically significant and was in the opposite direction from expected (adjusted OR, 1.18; 95% CI, 0.44-3.20; *P* = .74) (Table 3). Patients with NSCL were more likely than those with breast cancer to receive PP-CSF (adjusted OR, 11.35; 95% CI, 3.10-41.5). Most intervention clinics (13 of 23 clinics) did not prescribe any CSF for low-risk patients, but 4 clinics had rates greater than 0% and less than or equal to 10%, and 6 clinics had rates greater than 10% and less than 20%. However, after adjustment for other factors, clinics did not differ significantly according to the random effects variance. A GEE model (eTable 1 in Supplement 2) showed similar results and estimated the ICC to be 0.008 for clinic.

To better understand these results, we analyzed temporal trends in PP-CSF use and PP-CSF use by age (>65 vs ≤65 years). Over time, UC site adherence to guidelines increased for both low-risk and high-risk regimens, whereas intervention sites became less adherent in the low-risk group (eTable 2 in Supplement 2). PP-CSF use with low-risk regimens tended to be much higher among patients older than 65 years in both UC and intervention groups, a finding consistent with the literature^14,20,21^ (eTable 3 in Supplement 2). We found a high degree of concordance of PP-CSF use within clinics as indicated by the high variance in the mixed models and the high ICC in the GEE models.

### FN Rates

FN could be assessed for 1255 high-risk patients and 579 low-risk patients (Table 2). For high-risk patients, the FN rate was 4.2% (13 of 308 patients) for the UC group and 58 of 947 patients (6.1%) in the intervention group, and the difference was not statistically significant (adjusted OR, 1.49; 95% CI, 0.75-2.95; *P* = .26) (eTable 4 in Supplement 2). Although observed FN rates by clinic varied from 0% to 15.4%, with a median (IQR) of 5.3% (1.5%-8.8%), the analysis did not show significant variation beyond chance after adjustment for other variables. The ICC was very low, 0.003, so the FN rate does not depend on clinic (eTable 4 in Supplement 2). The FN rate among high-risk patients in the cohort group was slightly higher at 6.5% (25 of 384 patients). For low-risk patients, the FN rate was 0.8% (1 of 127 patients) in the UC group and 1.5% (7 of 452 patients) in the intervention groups, with no statistically significant difference (adjusted OR, 2.00; 95% CI, 0.23-18.80; *P* = .51) (eTable 4 in Supplement 2). In the cohort, the FN rate for low-risk patients was slightly higher at 1.7% (2 of 121 patients). eTable 5 in Supplement 2 shows FN rates conditional on assignment to PP-CSF prescribing, with rates of 4.7% (54 of 1141 patients) among high-risk patients and 0% (0 of 34 patients) among low-risk patients. eTable 6 in Supplement 2 shows FN rates conditional on no assignment to PP-CSF prescribing, with higher FN rates of 14.9% (17 of 114 patients) among high-risk patients and 1.5% (8 of 545 patients) among low-risk patients.

## Discussion

With the goal of improving adherence to evidence-based guidelines in PP-CSF prescribing, we designed the TrACER pragmatic cluster RCT to test an EHR-enabled, guideline-informed, automated, standing-order prescribing protocol for PP-CSF compared with UC. Contrary to expectation, there was no significant improvement in PP-CSF use or reduction in FN rates among high-risk patients enrolled at intervention sites. Overall adherence to PP-CSF prescribing in both groups was high. Similarly, among patients receiving low-risk regimens, PP-CSF use and FN rates were low and similar between the randomized groups. For cohort patients, PP-CSF use and FN rates were largely similar to that observed in the RCT.

The study results were unexpected in the context of prior studies. Among high-risk patients in the UC and intervention groups, approximately 90% were prescribed PP-CSF, a level of adherence far higher than previous studies where adherence varied between 18% and 58% of high-risk patients being prescribed PP-CSF.^2,3,4^ Adherence for low-risk patients was also very good, with only 5.5% of patients in the UC group and 6.3% of patients in the intervention group receiving PP-CSF. The results suggest that adherence to PP-CSF guidelines has improved substantially in community-based practices over time and may be associated with several factors. First, during this time frame, increased awareness of guidelines as a result of Choosing Wisely campaigns may have altered use.^22,23^ Second, increased availability of pegylated CSF and branded kits for outpatient administration may have decreased CSF administration burden.^24,25,26^ Finally, EHRs may have simplified ordering and standardized treatments without specific order entry requirements. Further study of interventions to improve PP-CSF guideline adherence may not be warranted.

It is unclear why guideline adherence was numerically lower in the intervention group. Because clinicians had to manually override standing orders, they were making a tacit decision that they did not agree with the guidelines recommendations. There were no patient characteristics or regimens for which standing orders were overridden more frequently.

We believe the results of this study have several important implications for health care decision-making. First, our study calls into question the value of EHR-enabled order entry algorithms in clinical decision-making for community oncology practices where guidelines are based on strong evidence. Order entry algorithms have been evaluated in other settings—notably, primary care—to improve antibiotic prescribing,^27,28^ appropriate ordering of imaging studies,^29^ and diabetes care,^30^ among many examples, with mixed results. Far fewer high-quality studies have evaluated order entry interventions in oncology practice, although some have shown positive results for quality of cancer care delivery.^31^ Notably, the TrACER study adhered to recommendations for developers of guideline-based clinical support systems^32,33^ and avoided several issues regarding physician, setting, and guideline contexts that have been problematic in other US-based studies.^34^ Some issues that have been cited as barriers to acceptance, especially impact on professional autonomy or medical legal concerns, were not explicitly addressed as part of this study.^35^ An additional potential confounding factor, automatic updates to PP-CSF prescribing recommendations by the National Comprehensive Cancer Network, may have caused some concern among practitioners who were familiar with earlier guidelines, since the newer guidelines reassigned several regimens to lower FN risk categories. At minimum, this study should inform clinical decision support system developers in oncology when considering whether the benefits in terms of changes in adherence to recommended care justify the considerable resources needed to develop, test, and implement such systems.

### Limitations

We note the limitations of this study. First, clinics that participated in TrACER were a voluntary subset of NCORP clinics. It is possible that the reasons clinics chose to participate were influenced by their interest in or experience with CSF. The recently published study^36^ of the Centers for Medicare & Medicaid Services’ Oncology Care Model also found high baseline levels of adherence to PP-CSF, although both that study and ours may reflect practices that are higher performing overall. Knowledge of the study hypothesis may have changed their prescribing behavior (ie, the Hawthorn Effect). The findings from the study may not apply to practice settings that are distinct from the community practices that participated (eg, academic practices). Additionally, the study sample was limited to patients with breast, NSCL, and colorectal cancer, and the applicability of the findings to other cancer sites is unknown. Furthermore, it is important to note that most patients in the high-risk group were female patients with breast cancer receiving dose-dense chemotherapy. PP-CSF is typically embedded in these regimens because of the very high risk of FN for these patients. There were far fewer patients with lung cancer in the study, where there are more patients with other FN risk factors such as age and comorbidity.

## Conclusions

In a large, prospective, cluster RCT of an automated order entry system for PP-CSF prescribing, the intervention did not improve prescribing adherence or FN rates among patients with NSCL, breast, and colorectal cancer receiving first-line myelosuppressive chemotherapy. Overall adherence to clinical practice guidelines was higher than reported historically, suggesting either a selection effect or a temporal trend toward improved prescribing. Use of guideline-based computerized clinical decision support systems in this setting was ineffective and, therefore, not recommended for community oncology practice settings.

## Notes

Supplement 1.
Trial Protocol

Supplement 2. **eTable 1.** PP-CSF Use: Comparison of Mixed and GEE Models: High and Low-Risk Patients **eTable 2.** Temporal Trends in PP-CSF Use, Assessed Using Registration Year in High-Risk and Low-Risk Patients **eTable 3.** CSF Use by Age (≤65 Years vs >65 Years) in (a) High-Risk and (b) Low-Risk Patients **eTable 4.** Febrile Neutropenia: Comparison of Mixed and GEE Models: High and Low-Risk Patients **eTable 5.** Febrile Neutropenia by Chemotherapy Risk Group, Conditional on CSF Prescription **eTable 6.** Febrile Neutropenia by Chemotherapy Risk Group, Conditional on No CSF Prescription

Supplement 3.
Data Sharing Statement
