# Supplementary material for: Effects of a Guideline-Informed Clinical Decision Support System Intervention to Improve Colony-Stimulating Factor Prescribing: A Cluster Randomized Clinical Trial
Source: JAMA Netw Open. 2022 Oct 24;5(10):e2238191. doi: 10.1001/jamanetworkopen.2022.38191 (PMC9593234; doi:10.1001/jamanetworkopen.2022.38191)
Supplement: Supplement 1. — Trial Protocol [file jamanetwopen-e2238191-s001.pdf]

PRIVILEGED COMMUNICATION
FOR INVESTIGATIONAL USE ONLY

Activation Date October 7, 2016

SWOG

A PRAGMATIC TRIAL TO EVALUATE A GUIDELINE-BASED COLONY STIMULATING FACTOR STANDING ORDER INTERVENTION AND TO DETERMINE THE EFFECTIVENESS OF COLONY STIMULATING FACTOR USE AS PROPHYLAXIS FOR PATIENTS RECEIVING CHEMOTHERAPYWITH INTERMEDIATE RISK FOR FEBRILE NEUTROPENIA –TRIAL ASSESSING CSF PRESCRIBING EFFECTIVENESS AND RISK (TRACER)

NCT #02728596

STUDY CHAIRS:

Scott D. Ramsey, M.D., Ph.D. (Internal Medicine)
Fred Hutchinson Cancer Research Center
1100 Fairview Avenue N, M3-B232
PO Box 19024
Seattle, WA 98109
Phone: 206/667-7846
FAX: 206/667-5977
mail: sramsey@fredhutch.org

Dawn L. Hershman, M.D., M.S. (Oncology)
University Medical Center
Fort Washington
10th Floor, Room 1068
New York, NY 10032
Phone: 212/305-1945
FAX: 212/ 305-0178
E-mail: dlh23@columbia.edu

Gary H. Lyman, M.D., MPH (Oncology)
Hutchinson Cancer Research Center
1100 Fairview Ave N, M3-B232
Seattle, WA 98109
Phone: 206/667-6670
FAX: 206/667-5977
E-mail: glyman@fredhutch.org

Sean D. Sullivan, Ph.D. (Pharmacy)
University of Washington
1959 NE Pacific Street
Box 257631
Seattle, WA 98195-7630
Phone: 206/685-8153
FAX: 206/543-3835
E-mail: sdsull@u.washington.edu

BIOSTATISTICIANS:

Aasthaa Bansal, Ph.D. (Biostatistics)
University of Washington
1959 NE Pacific Street
Seattle, WA 98195-7630
Phone: 206/685-9658
FAX: 206/543-3835
E-mail: abansal@uw.eduE-

William E. Barlow, Ph.D. (Biostatistics) Columbia
SWOG Statistics and Data Management Center161
1730 Minor Avenue, Suite 1900
Seattle, WA 98101-1468
Phone: 206/652-2267
FAX: 206/652-4612
E-mail: williamb@crab.org

Kathryn Arnold, M.S. (Biostatistics)
SWOG Statistics and Data Management CenterFred
Fred Hutchinson Cancer Research Center
1100 Fairview Avenue North, M3- C102
PO Box 19024
Seattle, WA 98109-1024
Phone: 206/667-7685
FAX: 206/667-4408
E-mail: karnold@fredhutch.org

SWOG Authorized Document

PARTICIPANTS

The following **SWOG, ALLIANCE, ECOG-ACRIN, and NRG** NCORP Components and Subcomponents who have submitted the **S1415CD** Component Application and have been approved for participation:

Group 1 (Cohort): (Expedited review allowed)

IL168/Carle Cancer Center NCORP-Carle Ca Ctr
Subcomponents:
IL393/CPG-Mattoon/Charleston
IL405/Carle on Vermilion-DanvilleIL406/CPG-Effingham
KS034/Wichita NCORP-Cancer Center of Kansas-Wichita
Subcomponents:
KS036/Cancer Center of Kansas - Dodge City
KS050/Cancer Center of Kansas – Winfield
KS051/Cancer Center of Kansas – Chanute
KS052/Cancer Center of Kansas – Newton
KS053/Cancer Center of Kansas – Wellington
KS054/Cancer Center of Kansas – Salina
KS063/Cancer Center of Kansas – Pratt
KS066/Cancer Center of Kansas-Kingman
KS067/Cancer Center of Kansas Wichita Medical Arts Tower - Wichita
KS073/Cancer Center of Kansas – Parsons
KS075/Cancer Center of Kansas - El Dorado
KS091/Ca Ctr of Kansas-Independence
KS094/Cancer Center of Kansas - Fort Scott
KS097/Cancer Center of Kansas-Liberal
MI080/CRC West MI NCORP-West Michigan Cancer Center
MO021/Ozarks NCORP-Mercy Hospital St. Louis
MO042/Ozarks NCORP-Cox Health South-Oncology Hematology Assoc.
MO043/Ozarks NCORP-Mercy Hospital Springfield
NC047/Southeast COR NCORP-Novant Health Forsythe Medical Center
Subcomponents:
NC048/Novant Health Oncology Specialists
NC249/Novant HOC
NC250/Novant HOC Mount Airy
NC251/Novant HOC Wilkesboro
NC252/Novant HOC Kernersville
NC262/Novant-Statesville
NE022/CORA NCORP-CHI Health Saint Francis
OH182/Columbus NCORP-Adena Regional Medical Center
OH252/Dayton NCORP-Dayton Physicians LLC-Samaritan North
Subcomponents:
OH156/Greater Dayton CC
OH465/Dayton Physicians LLC-Miami Valley South
OH466/Dayton Physicians LLC-Upper Valley
OH467/Dayton Physicians LLC-Wayne
OH468/Dayton Physicians LLC-Wilson
OH469/Dayton Physicians LLC-Signal
OH471/Dayton Physicians LLC-Atrium
SC024/Southeast COR NCORP-Spartanburg Medical Center:
Subcomponents:
SC095/Gibbs Cancer Center - Gaffney
SC101/Gibbs Cancer Center - Greer
SC109/MGC Hematology Oncology - Union
SC045/Greenville NCORP-Greenville Memorial Hospital
Subcomponents:
SC036/Greenville HS Ca Inst – Eastside
SC053/Greenville HS Ca Inst – Butternut
SC054/Greenville HS Ca Inst – Seneca
SC056/Greenville HS Ca Inst – Spartanburg
SC060/Greenville HS Ca Inst – Faris
SC085/Greenville HS Ca Inst – Greer
UT007/McKay-Dee Hospital
UT009/Logan Regional Hospital **UT056**/Northwest NCORP-Intermountain MedicalCenter
Subcomponent:
UT010/Dixie Medical Ctr Regional Ca Ctr
WA030/Northwest NCORP-Multicare Health System-Tacoma
Subcomponents:
WA029/Multicare Good Samaritan Hospital
WA085/Auburn Regional Medical Center
WA149/Rockwood Cancer Treatment Ctr-DHEC-Downtown
WA168/Rockwood Clinic Cancer Treatment Ctr-Valley
WA187/Gig Harbor Medical Park
WA215/Rockwood North Cancer Treatment Ctr

Group 2 (Usual Care): (Expedited review allowed)

IA024/Iowa-Wide NCORP-Oncology Associates at Mercy Medical Center **(ALLIANCE) ID003**/PCRC NCORP-Saint Luke's Mountain StatesTumor Inst
  Subcomponents:
  ID020/St. Luke's-Nampa
  ID021/St. Luke's-Meridian
  ID022/St. Luke's-Fruitland
  ID023/St. Luke's-Twin Falls
LA017/Gulf South MU NCORP-LSU Health Sciences Center-Shreveport
MI032/Michigan CRC NCORP-St. John Hospital & Medical Center
MI139/Michigan CRC NCORP-St. John Macomb Hospital
MO031/Kansas City NCORP-Research Medical Center
  Subcomponents:
  KS037/Menorah Medical Center
  MO092/Centerpoint Medical Center
NM022/New Mexico MU NCORP-Christus St. Vincent Regional Cancer Ctr
TN029/Baptist MU-NCORP (Baptist Memorial Hospital & Cancer Center – Memphis)
MS048/Family Cancer Center – Southhaven
MS053/Baptist Cancer Center – Golden Triangle
MS055/Baptist - DeSoto
MS056/Family Cancer Center - Oxford
MS057/Boston Baskin Cancer Center – Grenada
MS059/Baptist Memorial Hospital and Cancer Center - Union
TN066/Boston Baskin Cancer Center - Bartlett
TN173/Family Cancer Center Foundation - Memphis
TN198/Integrity Oncology - Collierville
AR019/Fowler Family Center for Cancer Care
AR047/NEA Baptist Memorial Hospital
WA007/PCRC NCORP-Swedish Cancer Institute (Swedish Medical Center-First Hill)
  Subcomponents:
  WA002/Swedish Med Ctr-Edmonds
  WA009/Swedish Med Ctr-Ballard
  WA145/Swedish Med Ctr-Cherry Hill
  WA196/Swedish Ca Inst-Issaquah

SWOG Authorized Document

Group 3 (Intermediate Risk – CSF): (Expected review allowed)

CA463/Bay Area NCORP-Contra Costa Regional Medical Center
HI010/Hawaii MU NCORP-Tripler Medical Center
IL042/SHCC NCORP-MU-John H Stroger Jr Hospital of Cook County **(ECOG-ACRIN)** **IL101**/Heartland NCORP-Illinois Cancer Care-Peoria

Subcomponent:
IL352/Illinois Cancer Care – Bloomington
IL361/Illinois Cancer Care - Canton
IL362/Illinois Cancer Care - Carthage
IL366/Illinois Cancer Care - Eureka
IL188/Illinois Cancer Care - Galesburg
IL344/Illinois Cancer Care - Kewanee Clinic
IL355/Illinois Cancer Care - Macomb
IL277/Illinois Cancer Care - Ottawa Clinic
IL354/Illinois Cancer Care - Pekin
IL353/Illinois Cancer Care - Peru **IL358**/Illinois Cancer Care – Princeton **IL185**/Heartland NCORP-Cancer Care Specialists of Central Illinois

Subcomponents: **IL208**/Crossroads Cancer Center**IL385**/Centralia Oncology Clinic
IL413/Cancer Care Specialists of Illinois Swansea

MI005/Beaumont NCORP
MI128/Beaumont Hospital Troy
MN012/Essentia Health NCORP – St. Joseph's Med Ctr
MN024/Essentia Health NCORP – Essentia Health Cancer Center
ND009/EHCC – South University Clinic
NM005/New Mexico MU NCORP-Presbyterian Kaseman Hospital

Subcomponent:
NM046/Presbyterian Rust Medical Center/Jorgenson Cancer Center

PA052/Geisinger NCORP – Geisinger Medical Center **(NRG)**

Subcomponents: **(all NRG)**
PA043/Geisinger Lewistown Hospital
PA136/Geisinger Community Medical Center
PA138/Geisinger Wyoming Valley/Henry Ca Ctr
PA209/Geisinger Medical Ctr-Ca Ctr Hazleton
PA281/Geisinger Medical Group
PA473/Geisinger Medical Oncology-Pottsville
PA475/Geisinger Medical Oncology-Lewisburg
PA493 Selinsgrove

PR030/Puerto Rico MU NCORP – Doctors Cancer Center Manati **(ECOG-ACRIN)**
TN037/Baptist MU NCORP-Meharry Medical College
WI031/Wisconsin NCORP-Marshfield Clinic

Subcomponents:
WI009/Marshfield Clinic-Minocqua Center
WI069/Marshfield Clinic-Chippewa Falls Center
WI103/Marshfield Clinic-Wausau Center
WI109/Marshfield Clinic-Rice Lake Center
WI129/Marshfield Clinic-Wisconsin Rapids Center
WI132/Marshfield Clinic-Ladysmith Center
WI144/Marshfield Clinic-Weston Center
WI208/Marshfield Clinic – Eau Claire Center

WI210/Marshfield Clinic-Stevens Point

SWOG Authorized Document

Group 4 (Intermediate Risk – No CSF): (Expected review allowed)

GA106/Georgia NCORP-Lewis Cancer & Research Pavilion at St. Joseph's/Candler
Subcomponent:
SC081/South Carolina Cancer Specialists PC
GA020/Georgia Regents MU NCORP – Georgia Regents University Medical Center
HI005/Hawaii MU NCORP-Queen's Medical Center
IA072/Iowa-Wide NCORP-Medical Oncology and Hematology Associates, Des Moines **(ALLIANCE)**
LA002/Gulf South MU-NCORP-LSU Health Science Center-New Orleans
LA029/University Hospital
Michigan CRC NCORP
ID011/Michigan CRC NCORP
MI017/St. Mary Mercy Hospital-Livonia
MI013/St. Joseph Mercy Hospital –Ann Arbor
Subcomponents:
MI221/St. Joseph Mercy Brighton
MI294/ Saint Joseph Mercy Canton
MI327/Saint Joseph Mercy Chelsea
Montana NCORP
MT002/Montana NCORP-Billings Clinic Cancer Center
MT019/Montana NCORP-Bozeman Deaconess Cancer Center

ID011/St. Alphonsus Regional Medical Center
ID009/St. Alphonsus – Nampa
ID025/Idaho Urology Inst – Meridian
ID027/St. Alphonsus CCC – Caldwell
ID030/Walter Knox Mem Hospital – Emmett
OR133/St. Alphonsus – Ontario
OR134/St. Alphonsus - Baker

Sanford NCORP (ALLIANCE) including:
IA034/Siouxland Regional Cancer Center
MN137/Sanford-Thief River Falls
MN138/Sanford Cancer Center-Worthington
MN089/Sanford Clinic-Bemidji
ND003/Sanford Medical Center-Fargo
ND005/Sanford Roger Maris Cancer Center
ND007/Sanford Bismarck Medical Center
SD003/Sanford USD Medical Ctr-Sioux Falls
SD004/Sanford Cancer Center Oncology Clinic

NM004/New Mexico MU NCORP-University of NewMexico Cancer Center
NY024/Columbia MU NCORP-Columbia University/Herbert Irving Cancer Center

TABLE OF CONTENTS

TITLE 1
PARTICIPANTS 2
TABLE OF CONTENTS 6
CANCER TRIALS SUPPORT UNIT (CTSU) ADDRESS AND CONTACT INFORMATION 8
COMPONENT SCHEMA 9
1.0 OBJECTIVES 10
1.1 Primary Objective(s) 10
1.2 Secondary Objective(s) 10
1.3 Additional Objective(s) 10
2.0 BACKGROUND 11
3.0 DRUG INFORMATION 14
4.0 STAGING CRITERIA 14
5.0 ELIGIBILITY CRITERIA FOR PATIENTS 14
5.1 Disease Related Criteria 14
5.2 Prior/Concurrent Therapy Criteria 14
5.3 Clinical Criteria 15
5.4 Regulatory Criteria 15
6.0 STRATIFICATION FACTORS 15
7.0 STUDY PROCEDURES 15
7.1 General Study Description 15
7.2 Modification of Components' Electronic Medical Record (EMR) Systems 17
7.3 Process for Assessing Febrile Neutropenia Risk Level 20
7.4 Patient Recruitment and Registrations 21
7.5 Data Collection and Patient Questionnaires 21
7.6 Follow-Up Period 21
7.7 Data Reporting 21
8.0 TOXICITIES TO BE MONITORED AND DOSE MODIFICATIONS 21
8.1 NCI Common Terminology Criteria for Adverse Events Occurring with Colony Stimulating Factors(CSFs) 21
8.2 Dose Modifications 22
9.0 STUDY CALENDAR 23
10.0 CRITERIA FOR EVALUATION AND ENDPOINT ANALYSIS 25
10.1 Primary Endpoints 25
10.2 Other Endpoints 26
10.3 Performance Status 26
11.0 STATISTICAL CONSIDERATIONS 26
11.1 Estimate of Sample Size 26
11.2 Analysis of Primary Objectives 29
11.3 Analysis of Secondary Objectives 31
11.4 Analysis of Additional Objectives 34
11.5 Intent-to-Treat Analysis 34
11.6 Missing Data 34
11.7 Heterogeneity of Treatment Effect 35
11.8 Data and Safety Monitoring 35
11.9 Interim Analysis 35
12.0 DISCIPLINE REVIEW 36
13.0 REGISTRATION GUIDELINES 36
13.1 Registration Timing 36
13.2 Investigator/Site Registration Procedures 36
13.3 CTEP Registration Procedures 36
13.4 CTSU Registration Procedures 37
13.5 Oncology Patient Enrollment Network (OPEN) Registration Requirements 39
13.6 Exceptions to SWOG registration policies will not be permitted. 41
14.0 DATA SUBMISSION SCHEDULE 41
14.1 Data Submission Requirement 41
14.2 Master Forms 41

SWOG Authorized Document

14.3 Data Submission Procedures 41
14.4 Data Submission Overview and Timepoints 42
15.0 SPECIAL INSTRUCTIONS 44
15.1 Component Application, Review, and Participation 44
15.2 Administration of Patient-Completed Questionnaires 45
15.3 Optional Training for Administration of Patient-Completed Questionnaires 45
16.0 ETHICAL AND REGULATORY CONSIDERATIONS 46
16.1 Adverse Event Reporting Requirements 46
17.0 BIBLIOGRAPHY 50
18.0 APPENDIX 54
18.1 **S1415CD** List of Protocol Approved Regimens 55

SWOG Authorized Document

CANCER TRIALS SUPPORT UNIT (CTSU) ADDRESS AND CONTACT INFORMATION

CONTACT INFORMATION

**For regulatory requirements:**	**For patient enrollments:**	**For study data submission:**
Regulatory documentation mustbe submitted to the CTSU via the Regulatory Submission Portal:<br><br>(Sign in at www.ctsu.org, and select the Regulatory > Submission.)<br><br>Institutions with patients waiting that are unable to use the Portal should alert the CTSU Regulatory Office immediately at 1-866-651-2878 to receive further instruction and support.<br><br>Contact the CTSU Regulatory Help Desk at 1-866-651-2878for regulatory assistance.	Refer to the patient enrollment section of the protocol for instructions on using the Oncology Patient Enrollment Network (OPEN). OPEN can be accessed at https://www.ctsu.org/OPEN_SYSTEM/ or https://OPEN.ctsu.org.<br><br>Contact the CTSU Help Desk with any OPEN-related questions at ctsucontact@westat.com.	Data collection for this study will be done exclusively through Medidata Rave. Refer to the data submission section of the protocol for further instructions.<br><br>Other Tools and Reports:<br>Institutions participating through the CTSU continue to have access to other tools and reportsavailable on the SWOG CRA Workbench via the SWOG website (www.swog.org).

The most current version of the **study protocol and all supporting documents** must be downloaded from the protocol-specific Web page of the CTSU Member Web site located at https://www.ctsu.org.

Access to the CTSU members' website is managed through the Cancer Therapy and EvaluationProgram – Identity and Access Management (CTEP-IAM) registration system and requires user log on with CTEP-AIM username and password.

For patient eligibility questions or data submission questions contact the SWOG Statistics andData Management Center by phone or email:

206-652-2267
cancercontrolquestion@crab.org

For study procedure related questions contact the SWOG Statistics and Data Management Center byphone or e-mail.

For questions unrelated to patient eligibility, site requirements or data submission contact theCTSU Help Desk by phone or e-mail:

CTSU General Information Line:
1-888-823-5923
ctsucontact@westat.com

All calls and correspondence will be triaged to the appropriate CTSU representative.

The CTSU Website is located at https://www.ctsu.org.

COMPONENT SCHEMA

SWOG, Alliance, NRG and ECOG-ACRIN NCORP COMPONENTS AND SUBCOMPONENTS [1]

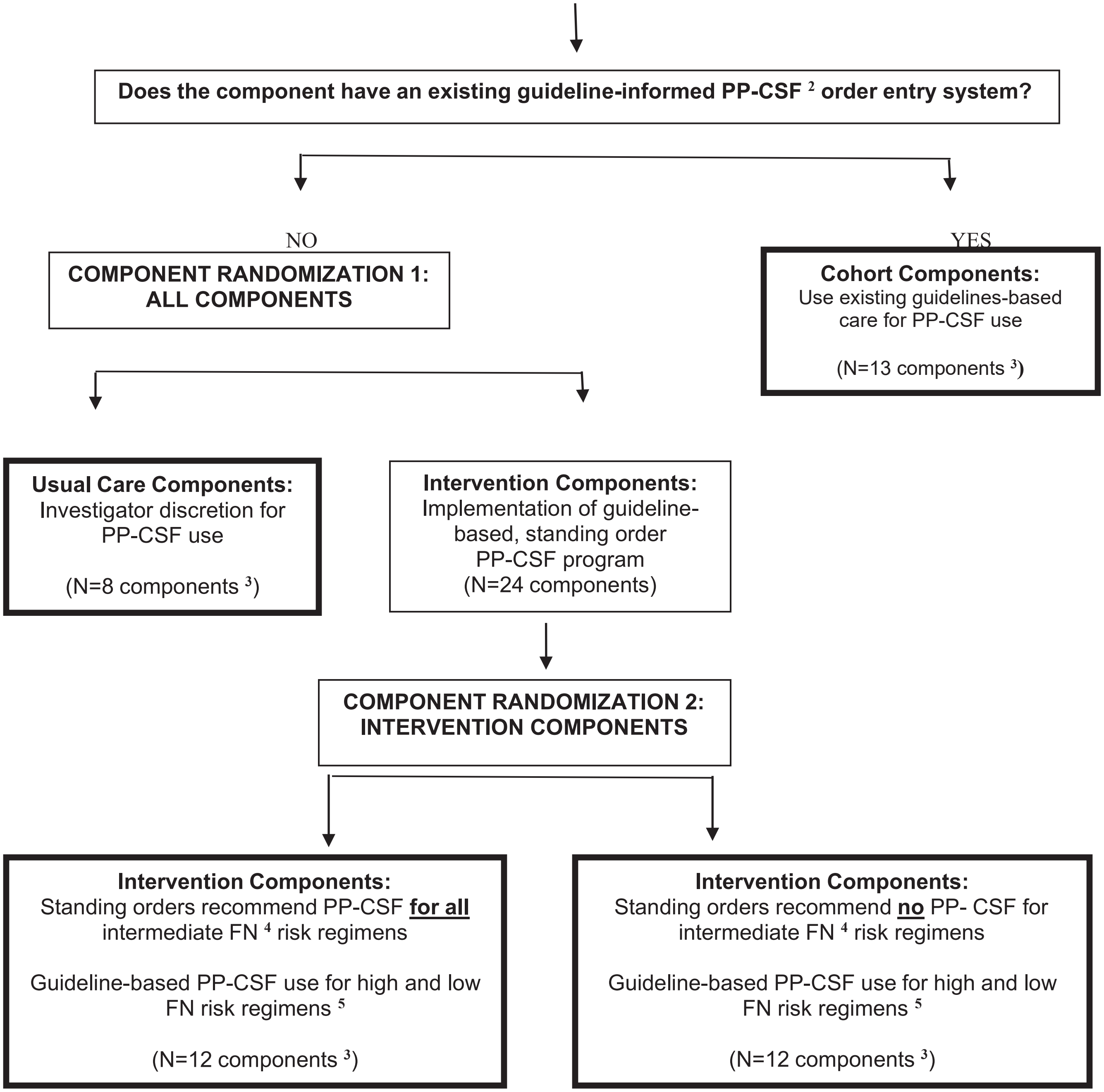

[1] **Eligible CCDR components must have submitted the S1415CD Component Application andbeen approved for participation.**

[2] **Primary Prophylactic Colony Stimulating Factor.**

[3] **All patients at participating components will be subject to the PP-CSF use care as determined by component assignment (Usual Care, Intervention, or Cohort). Only consented patients registered to the study will**

SWOG Authorized Document

participate in the data collection.
[4] **Febrile neutropenia.**
[5] **As determined at point-of-care by the guideline-based standing order PP-CSF program (see Appendix 18.1)**

1.0 OBJECTIVES

1.1 Primary Objective(s)

a. To compare the use of primary prophylactic colony stimulating factor (PP-CSF) according to recommended clinical practice guidelines among patients registered at Intervention components versus Usual Care components.

b. To compare the rate of febrile neutropenia (FN) among patients registered at Intervention components versus Usual Care components.

c. To compare the rate of FN among intermediate risk patients registered at Intervention components by component treatment assignment (administer PP- CSF to intermediate risk patients versus not).

1.2 Secondary Objective(s)

a. To compare the rate of FN among low-risk patients registered at Intervention components versus Usual Care components.)

b. To compare the FN-related health-related quality of life (HRQL) among low-risk patients registered at Intervention components versus Usual Care components.

c. To compare patient adherence to PP-CSF prescribing among patients registered at Intervention components versus Usual Care components.

d. To compare patient knowledge of the indications for, efficacy of, and side effects associated with PP-CSF between the initiation and conclusion of the first cycle of myelosuppressive systemic therapy among patients registered at Intervention components versus Usual Care components.

e. To compare the proportion of patients completing the initial systemic therapy regimen at planned duration and at planned dose intensity among patients registered at Intervention components versus Usual Care components.

f. To compare antibiotic use both as prophylaxis and as treatment for FN among patients registered at Intervention components versus Usual Care components.

g. To compare the rate of FN-related emergency department visits and hospitalizations among intermediate risk patients registered to Intervention components by component treatment assignment (administer PP-CSF to intermediate risk patients versus not).

h. To compare the FN-related health-related quality of life (HRQL) among intermediate risk patients registered to Intervention components by component treatment assignment (administer PP-CSF to intermediate risk patients versus not).

i. To compare overall survival among intermediate risk patients registered to Intervention components by component treatment assignment (administer PP- CSF to intermediate risk patients versus not).

1.3 Additional Objective(s)

a. To characterize and descriptively report the differences among Cohort components and the Intervention and Usual Care components, according to the endpoints outlined in Section 10.0.

SWOG Authorized Document

b. To evaluate the time to invasive recurrence in non-metastatic patients bycomponent treatment assignment.

2.0 BACKGROUND

Complications during chemotherapy are a major source of morbidity, mortality and cost for cancerpatients. Among the nearly 600,000 US patients who will be diagnosed with breast, colorectal, or lung cancer this year, nearly half will be offered chemotherapy. *(1)* A substantial proportion of those patients are at risk of febrile neutropenia (FN), a serious condition that involves a fever and an abnormally low number of neutrophils in the blood. Chemotherapy regimens associated with an increased risk of FN are usually administered in a cyclical fashion over several weeks. The regimen type and the number of doses are related to the risk of FN. A patient can develop FN within 3-10 days after the initial administration of chemotherapy, when their neutrophil count reaches its nadir. Many patients with FN are hospitalized, and nearly 8% of those hospitalized for FN die. *(2)* In addition, patients experiencing FN commonly receive lower doses of chemotherapy on later cycles of treatment, potentially compromising the likelihood of remission orcure for their cancer. *(3)* FN is costly to patients, with additional expense ranging from $5,000 to $38,000 per episode and a mean hospital length of stay of more than 10 days. *(4)*

Colony-Stimulating Factors (CSFs) can be used to maintain the white blood cell count, and are commonly used to reduce the risk of FN, as well as its severity and duration. Clinical practice guidelines recommend prophylactic CSFs with the first cycle of chemotherapy—termed primary prophylactic CSF (PP-CSF) use—for patients receiving chemotherapy that carries a high risk of FN (>20%), and suggest “consideration” of PP-CSFs in conjunction with chemotherapy that has an intermediate risk (10-20%) of FN (Table 1).

Although evidence-based clinical practice guidelines for CSF have been available for nearly two decades, multiple studies show that the gap between best scientific evidence and clinical practiceis wide for PP-CSF prescribing: between 55% and 95% of PP-CSFs prescribing is inconsistent with guidelines. *(5,6,7,8,9,10,11,12,13)* Inappropriate prescribing occurs in both directions: underuse for high FN risk regimens (where PP-CSF is recommended); and overuse of PP-CSF for patients receiving low-risk chemotherapy (where PP-CSF is not recommended).

Table 1. ASCO recommendations for Primary Prophylactic CSF use in patients receiving chemotherapy. (Risk is based on regimen) *(14)*

Recommend use	Consider use	Do not recommend use
High-risk of FN (>20%)	Intermediate-risk of FN (10-20%)	Low-risk of FN (<10%)

When used in recommended settings, PP-CSF has clear benefit for patients—including reducing the risk of hospitalization and death due to infection—therefore it is critically important to ensure that PP-CSF is prescribed when supported by evidence. Researchers at the Hutchinson Institute for Cancer Outcomes Research (HICOR) have conducted several retrospective studies of community practice, finding that among breast cancer patients receiving chemotherapy carrying ahigh or intermediate risk of FN, PP-CSF use significantly reduced the FN rates. *(15)* In another study of 99 community oncology practices, HICOR found that cancer patients receiving PP-CSF within 3 days of chemotherapy (i.e., as primary prophylaxis) had much lower rates of FN than situations where CSF was used after 3 days in any chemotherapy cycle or not at all. *(16)*

Overuse and underuse of PP-CSF both expose patients to unnecessary risks. HICOR and others have published several observational studies finding that PP-CSF is widely under and over utilized in community settings. *(17,18,19,20,21)* For example, in a retrospective claims analysisof 2,728 cancer patients diagnosed over a four year period in Washington State, HICOR researchers

SWOG Authorized Document

found that PP-CSF was underutilized for patients receiving chemotherapy carrying a high FN risk:only 28% of breast cancer and 1.5% of lung cancer patients receiving high-risk chemotherapy were prescribed PP-CSF. *(22)* Underuse appears to be common in certain groups (the elderly, low socioeconomic status), and needlessly increases patients' risk for FN. Overuse exposes patients to side effects, primarily bone and muscle pain, and potentially high out of pocket costs, with unclear benefits. *(23)* The HICOR study found that over 16.5% of colorectal and 20% of non-small cell lung cancer patients received CSF alongside chemotherapy regimens carrying low risk of FN. *(24)*

Non-adherence to guidelines may be partially explained by an insufficient evidence base supporting their recommendations. The evidence base underpinning clinical practice guidelines islargely based on FDA registration trials involving high risk chemotherapy regimens; however, the FN risk for chemotherapy regimens most commonly given in the community today are thought to be lower than those used in the trials. *(25,26)* While one prospective controlled clinical trial evaluated PP-CSF use in breast cancer patients receiving intermediate risk chemotherapy, the regimen studied is not commonly used today. *(27)*

A further complication is that most guidelines recommend consideration of "patient factors" (e.g., age, comorbid illness) alongside chemotherapy regimen when estimating the risk of FN, yet thereis little high quality evidence supporting the impact of patient-specific factors on FN risk. A final issue is conflict between guidelines as to whether the particular regimen is low, intermediate, or high risk. *(28,29,30)* To better support clinician and patient decisions, there is an urgent need to improve the evidence base for PP-CSF with modern chemotherapy regimens.

Lack of adherence with guidelines has significant financial implications for patients and the health care system

Colony stimulating factor is financially burdensome for cancer patients and families who are already at risk for financial toxicity and even bankruptcy. *(31)* In 2010, the Centers for Medicare and Medicaid Services (CMS) paid $888 million for CSF, its 6th largest individual Part B drug expenditure. *(32)* These issues are not likely to change with the advent of short acting biosimilars (currently tbo-filgrastim) as the price is only modestly below branded prices and long acting agents (Neulasta®) dominate the market. Out of concern of overprescribing PP-CSF, the American Society of Clinical Oncology (ASCO) and American Board of Internal Medicine include PP-CSF in its widely cited "Choosing Wisely" list of "10 Things Physicians and Patients Should Question." *(33,34)*

This pragmatic trial is designed both to test an intervention to increase compliance with guidelines, while also generating high quality evidence to assess effectiveness of PP-CSF on reducing rates of FN for patients receiving intermediate-risk chemotherapy regimens. The persistent problems of overuse and underuse of CSF, the financial burden of CSF to patients, and the gap in evidence regarding the effectiveness of PP-CSF in the intermediate risk setting, make a compelling case for the proposed study. Despite nearly twenty years of recognition that PP-CSF prescribing is suboptimal, there have been very few studies of interventions to improveits use in clinical practice. *(35,36)* One nonrandomized, pre-post evaluation of a peer to peer consultation protocol designed to reduce inappropriate CSF use among 22 community oncology practices reduced the use of PP-CSF among low-risk chemotherapy patients by 71% percent.
(37) We are not aware of prospective, controlled studies designed to align PP-CSF use with clinical practice guidelines.

This study addresses gaps in evidence at both the patient and health-system levels, and providesan important opportunity to improve cancer patient outcomes while guiding more appropriate, effective care. For health care delivery systems, this pragmatic study will address the question of whether implementing order entry systems for supportive care alongside chemotherapy improves evidence-based prescribing and patient outcomes. For clinicians and patients, this study will greatly improve evidence base around the effectiveness of PP-CSF for most commonly prescribed regimens for the most common solid tumors, thereby supporting clinical decision- making.

SWOG Authorized Document

The specific objectives of this study address two vital evidence gaps in real world practice. This pragmatic study is designed to compare outcomes of PP-CSF use in community practice as currently managed by physicians and clinic staff (usual care) with care that includes a protocol- based, guideline-informed standing PP-CSF order system. The intent of the first study aim is to align PP-CSF prescribing with clinical evidence, where the benefit to patients is likely to be highest. The second specific aim will evaluate the effectiveness of PP-CSF in modern, intermediate-risk chemotherapy regimens, for which there is currently an evidence gap. The design of this study is innovative in that it generates evidence on an important patient-level clinical question within the context of evaluating the effectiveness of a system-level cancer care delivery intervention. Given the documented variation in CSF prescribing and attendant impact onpatient outcomes, this pragmatic trial has the potential to provide substantial benefit to many of the 275,000 breast, lung and colorectal cancer patients who receive myelosuppressive chemotherapy each year. *(38,39,40)*

Cohort Study

The cohort study will provide a parallel observational study of CSF use in community practice as heterogeneous prescribing protocols in the usual care arm will make comparison difficult. The cohort study serves several specific purposes. First, while it is known that some practices and systems have implemented electronic order entry for CSF prescribing, the degree to which these order systems adhere to guidelines, apply to relevant patient populations, or are impacting CSF prescribing are not known. Some systems will be similar enough to the study intervention to render component sites ineligible for the randomized control trial. The **S1415CD** Study Team at the SWOG Statistical Center would like to see how the study approach compares to these sites. Second, this will allow tracking of trends in CSF prescribing over time, comparing the impact of the stakeholder-driven intervention with the others, both in terms of their effectiveness, and how they affect trends in CSF use and related outcomes over time. Finally, the cohort study will facilitate understanding of post-randomized control trial implementation of the intervention if it is effective.

Inclusion of Women and Minorities

This study was designed to include women and minorities, but was not designed to measure differences of intervention effects. The anticipated accrual in the ethnicity/race and sex categories is shown in the table below.

Racial Categories	Ethnic Categories				Total
	Not Hispanic or Latino		Hispanic or Latino		
	Female	Male	Female	Male	
American Indian/ AlaskaNative	21	4	7	1	33
Asian	136	29	3	0	168
Native Hawaiian or Other PacificIslander	4	1	1	0	6
Black or African American	344	73	12	3	432
White	2438	517	270	56	3281
More Than One Race	28	6	5	1	40
**Total**	2971	630	298	61	3960

SWOG Authorized Document

3.0 DRUG INFORMATION

The choice of CSF on this study is at the discretion of the treating physician, but the CSF used forprimary prophylaxis must be FDA-approved. Please refer to the package insert for the drugs that the individual patient will receive for approved language related to information on dosing, toxicities, preparation and administration of these agents.

4.0 STAGING CRITERIA

Refer to AJCC Cancer Staging, 7th Edition, breast cancer, non-small cell lung cancer, and colorectal cancer staging

5.0 ELIGIBILITY CRITERIA FOR PATIENTS

Patient eligibility requirements are the same for all participating components, regardless of component treatment assignment (Cohort, Intervention, and Usual Care).

Each of the criteria in the following section must be met in order for a patient to be considered eligible for registration. For each criterion requiring test results and dates, please record this information on the Onstudy Form and submit via Medidata Rave® (see Section 14.0). Any potential eligibility issues should be addressed to the SWOG Statistics and Data Management Center in Seattle at 206/652-2267 prior to registration. NCI policy does not allow for waiver of any eligibility criterion (http://ctep.cancer.gov/protocol(Development/policies_deviations.htm).

In calculating days of tests and measurements, the day a test or measurement is done is considered Day 0. Therefore, if a test is done on a Monday, the Monday 2 weeks later would be considered Day 14. Thisallows for efficient patient scheduling without exceeding the guidelines. **If Day 180 falls on a weekend or holiday, the limit may be extended to the next working day.**

5.1 Disease Related Criteria

a. Patients must have a current diagnosis of breast cancer, non-small cell lung cancer, or colorectal cancer. The current diagnosis may be an initial diagnosis or recurrence and/or progression of previously diagnosed disease. Cancer may be metastatic or non-metastatic.

5.2 Prior/Concurrent Therapy Criteria

a. Patients must be registered prior to or on the same day as their first cycle of chemotherapy for their current disease and stage (or disease setting). Patients must not have had any systemic therapy (chemotherapy or combination regimens) in the 180 days just prior to registration. Prior biologic therapy, immunotherapy, tyrosine kinase inhibitors, and hormonal therapy are allowed.

b. Patients must be planning to receive one of the study-allowed regimens listed in Appendix 18.1 as their initial treatment for their current disease. (Effective December 2, 2019, only registration of patients on regimens with intermediate FN risk will be allowed for randomized sites (Groups 2, 3 and 4. Refer to Partial Closure Notice for more information). Myelosuppressive therapy must follow the standard regimen, although a dose reduction of up to 10% is permitted. This treatment may be neoadjuvant or adjuvant chemotherapy. Patients must not be receiving or planning to receive concurrent radiation during systemic treatment.

SWOG Authorized Document

c. Patients must not have any known contraindication to CSFs prior to registration, including prior hypersensitivity to Escherichia coli-derived proteins, filgrastim, pegfilgrastim, or tbo-filgrastim.

5.3 Clinical Criteria

a. Patient must be at least 18 years of age.

b. Patients must be able to understand and provide information for the patient- completed study forms in either English or Spanish.

c. Patients may have had a prior malignancy.

d. Patients must not be participating or plan to participate in other clinical trials that involve investigational systemic cancer treatments or investigational uses of CSFduring their first 6 months after registration.

5.4 Regulatory Criteria

a. Patients must be informed of the investigational nature of this study and must sign and give written informed consent in accordance with institutional and federal guidelines.

b. As a part of the OPEN registration process (see Section 13.0 for OPEN access instructions) the treating institution's identity is provided in order to ensure thatthe current (within 365 days) date of institutional review board approval for this study has been entered in the system.

6.0 STRATIFICATION FACTORS

Randomized participating components will be randomly assigned to Usual Care or Intervention with stratification by component size (number of patients at that component) and type of component (Minority Underserved-NCORP vs non-Minority Underserved-NCORP).

7.0 STUDY PROCEDURES

7.1 General Study Description

Primary prophylactic CSF (PP-CSF) is defined as CSF administered with the first cycle ofsystemic therapy, within 24 to 72 hours after the initial systemic therapy dose. PP-CSF and general CSF use is observed and reported.

This study uses a cluster randomized design: the study intervention is administered at thecomponent level, rather than the patient level. The study intervention is to change the component's standing order system to reflect guidelines-based PP-CSF use, or to not change the order system. All patients at a given component will be subject to the same order system, regardless of study participation; only registered patients will have their data reported to the study. Treating physicians at each component may change any individual patient's orders at any time, at their discretion.

a. Randomized Components

The initial treatment regimen is defined as the systemic treatment intended for cancer treatment, at registration as determined by the treating physician and as defined in Section 18.1. Patients are followed for 12 months after registration,

SWOG Authorized Document

regardless of treatment status. This protocol does not direct systemic cancertreatment therapy.

SWOG Authorized Document

b. Cohort Components

During component application review (see Section 15.1), practices that are identified by the **S1415CD** Study Team as ineligible for the randomized trial because they are already using a guideline-based PP-CSF ordering system will be considered for the cohort study. A total of thirteen cohort practices will be selected and approved to participate. Patient eligibility, registration, enrollment and data collection procedures are identical for both the cohort and randomized components.

7.2 Modification of Components' Electronic Medical Record (EMR) Systems

Figure 1 shows an overview of how the study intervention will be implemented into default orders that appear in the affected EMR systems. The process for changing the order systems to include the standing orders will involve the following steps:

1) The **S1415CD** Study Team will work with the component Primary Investigator to establish a local project team and begin mapping out a local process for modifying the order set.
2) The local project team and **S1415CD** Study Team will establish a blueprint for order set implementation.

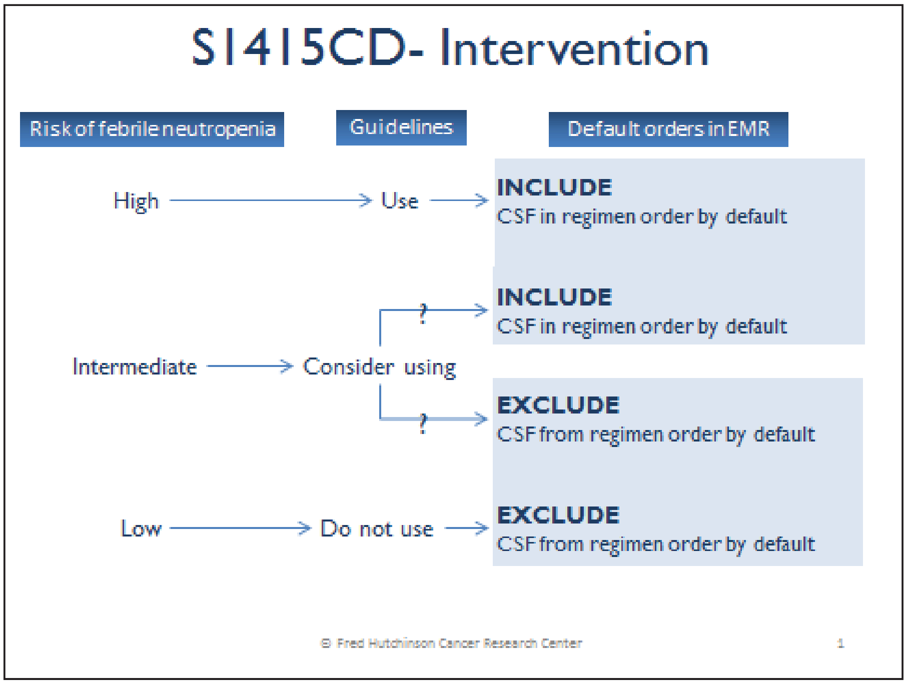

Figure 1. Overview of integration of standing orders into EMR

3) The local project team will perform analyst mock-up and test changes with end-users (e.g., pharmacist and clinic staff).
4) The local project team will implement changes in production clinical systemenvironment.

The **S1415CD** Study Team will provide technical assistance to components in the formsof EMR consultants, training workshops, and videos.

Below are screenshots showing how the standing orders could appear in the EMR or paper ordering system, for patients on high, low and intermediate risk regimens. The screenshots are for explanation only; ordering system appearance, content, and function will vary by vendor and hospital.

Example Order System- High Risk Regimen

In this example (Figure 2), the physician has prescribed a high-risk breast cancer regimen. CSF is included in the order by default. A text alert states the FN risk of the regimen and the recommendation for PP-CSF use. Pegfilgrastim is shown in the example, but any type of CSF may be used as the default.

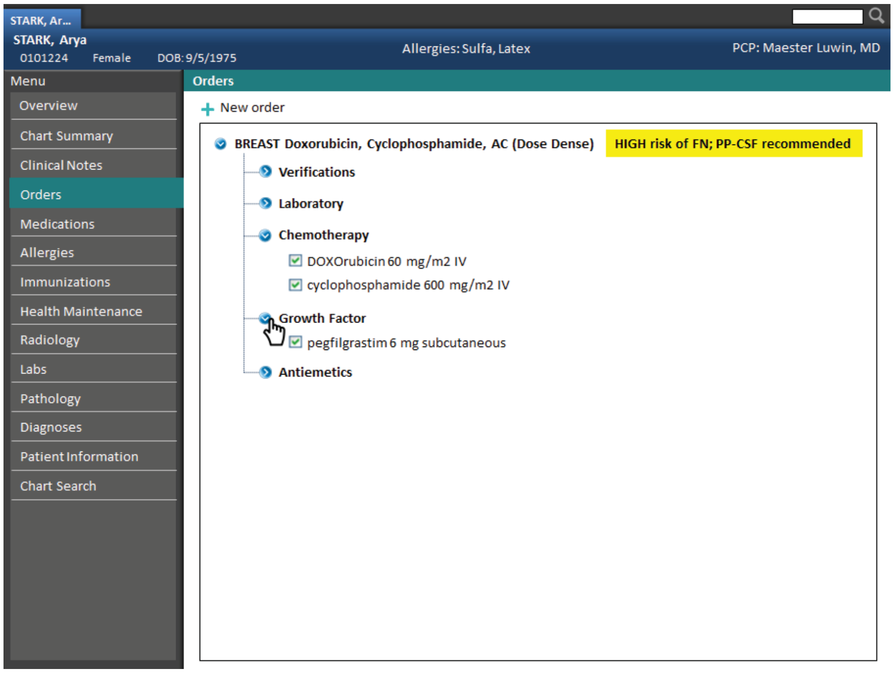

Figure 2

Example Order System- Low Risk Regimen

In this example (Figure 3), the physician has prescribed a low-risk breast cancer regimen.CSF is excluded from the order by default. A text alert states the FN risk of the regimen and the recommendation for PP-CSF use.

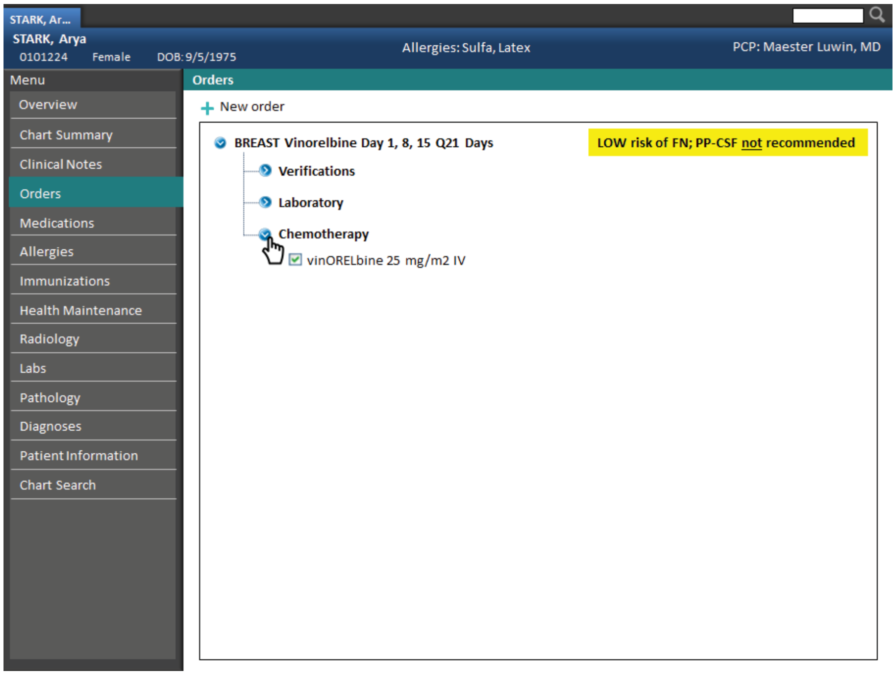
STARK, Ar...
STARK, Arya
0101224 Female DOB: 9/5/1975
Allergies: Sulfa, Latex
PCP: Maester Luwin, MD
Menu
Overview
Chart Summary
Clinical Notes
Orders
Medications
Allergies
Immunizations
Health Maintenance
Radiology
Labs
Pathology
Diagnoses
Patient Information
Chart Search
Orders
New order
BREAST Vinorelbine Day 1, 8, 15 Q21 Days
LOW risk of FN; PP-CSF not recommended
Verifications
Laboratory
Chemotherapy
vinORELbine 25 mg/m2 IV

Figure 3

Order System- Intermediate-Risk Regimen

What a physician will see if they prescribe an intermediate risk regimen depends on their study arm. If a component is randomized to include standing orders for PP-CSF for all intermediate risk regimens, default PP-CSF orders will appear on the screen in the same way as the default orders for high-risk regimens. If a component is randomized to not include standing orders for PP-CSF for intermediate-risk regimens, the screen will function in the same way as for low-risk regimens. The alert text will be modified to be specific to the intermediate risk classification of the regimen and the randomized assignment.

Order System- Paper

Paper ordering systems will have the same basic design as the EMR modification but willdiffer in the implementation strategy, as shown below in Figure 4.

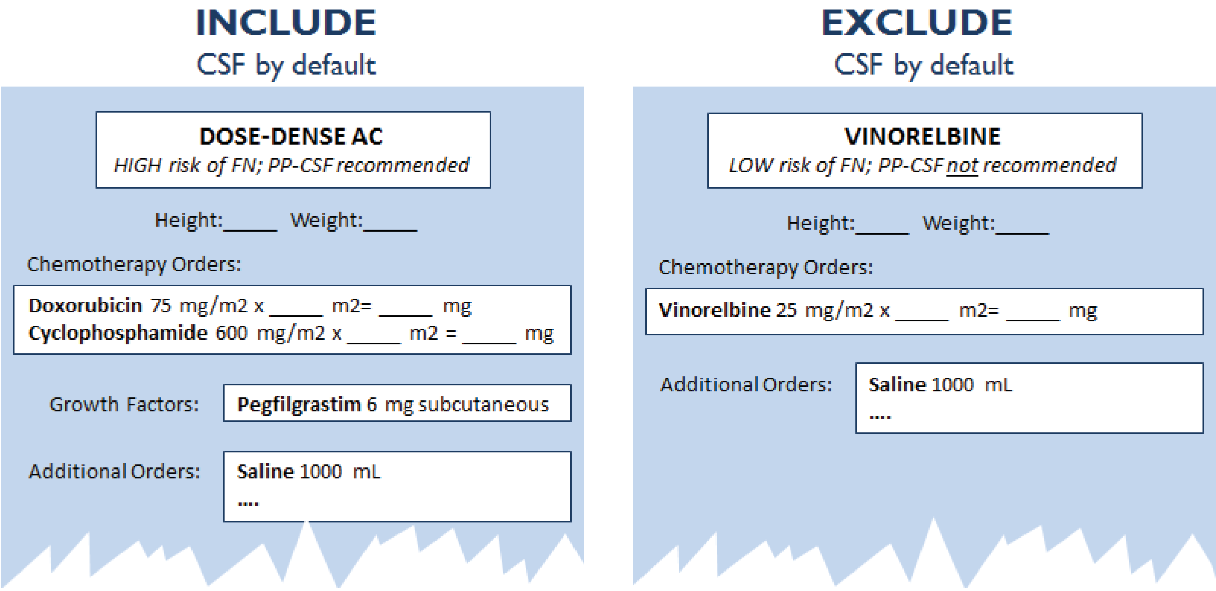

Figure 4

The treating physician may change an individual patient's orders from what is specifiedby following standard procedures for changing a prescription order when adding orremoving CSF. The specific procedures will depend on the EMR and local component preference.

7.3 Process for Assessing Febrile Neutropenia Risk Level

Patients' febrile neutropenia (FN) risk level in the order system will be determined by the febrile neutropenia (FN) risk algorithm developed by the **S1415CD** Study Team. The algorithm is based on current NCCN guidelines and peer-reviewed studies, with input from pharmacy and guidelines experts from the investigator team and its external stakeholder advisory group. The algorithm is regimen-based; patient comorbid factors do not inform the algorithm or regimen risk classification at point of order, but will becollected for analysis.

The chemotherapy regimens included in the algorithm are standard breast, non-small celllung, and colorectal cancer regimens pulled from standard NCCN order templates available on the NCCN website (www.NCCN.org). FN risk is expressed as three different categories: low (<10% risk of FN), intermediate (10-20%), and high (>20%). Risk levels for each regimen are determined by NCCN guidelines and/or peer-reviewed studies. The

list of study recognized regimens and their associated FN risk level is available in Section18.1.

When a physician orders a particular regimen, the regimen order set will automatically include or exclude CSF based on the FN risk algorithm classification for that regimen andthe intermediate risk randomization assignment at that component (see Figure 1 inSection 7.2).

7.4 Patient Recruitment and Registrations

Components may begin registering patients to the study after the component has received approval from the **S1415CD** Study Team to begin patient recruitment andregulatory administration is complete per Section 13.2 and Section 13.3.

Clinic research staff will work closely with the physician team to screen scheduledpatients and flag the charts of those who meet eligibility criteria for approach at their next clinic visit. To be eligible, patients must already have orders for a study-recognized chemotherapy regimen (see Section 18.1 for the list of regimens), but have not started the first cycle of their treatment. Depending on the timing of the chemotherapy orders relative to patient's scheduled appointments, patients will be approached at a clinic visit either before or on the day they are scheduled to begin treatment.

Staff will approach potential participants with information about the primary study and asktheir permission to participate. All eligible patients for the randomized trial and cohort study will be provided information and consent forms prior to enrollment. Tailored consentinformation will be provided for patients eligible for each of these groups. Approach procedures are the same, regardless of the study group assignment (Cohort,Intervention, or Usual Care).

7.5 Data Collection and Patient Questionnaires

Please refer to Sections 9.0, 14.0, and 15.0 for instructions regarding data collection and patient questionnaires.

7.6 Follow-Up Period

All patients will be followed until 12 months after registration, regardless of whether initial treatment regimen was discontinued or if CSFs were ever used. Refer to Section 14.4 for forms submission requirements.

7.7 Data Reporting

Time points after End of First Cycle are not patient visit driven, but instead are cumulativefrom date of registration up to the form specified time point. Therefore, the medical recordreview at the 1 Month, 6 Month and 12 Month time points must happen on or after the patient reaches 1 month, 6 months or 12 months past registration. (Refer to Sections 9.0 and 14.4 for Study Calendar and Data Submission information.)

8.0 TOXICITIES TO BE MONITORED AND DOSE MODIFICATIONS

8.1 NCI Common Terminology Criteria for Adverse Events Occurring with Colony Stimulating Factors (CSFs)

This study will utilize the CTCAE (NCI Common Terminology Criteria for Adverse Events)Version 4.0 for toxicity and Serious Adverse Event reporting related to CSFs. A copy of the CTCAE Version 4.0 can be downloaded from the CTEP home page (http://ctep.cancer.gov). All appropriate treatment areas should have access to a copy of the CTCAE Version 4.0.

SWOG Authorized Document

8.2 Dose Modifications

All treatments and dose modifications are at the discretion of the treating investigator,including PP-CSF and systemic therapy.

9.0 STUDY CALENDAR

	**Registration**	**After first cycle of systemic therapy**	**Mo1**	**Mo6**	**Mo 12**	**If initial treatment regimen isdiscontinued [2]**	**If patient dies**
**PATIENT QUESTIONNAIRES**							
**S1415CD** Baseline Patient Survey	X						
**S1415CD** Medical Conditions Questionnaire	X						
**S1415CD** FACT-N (Version 4)	X	X					
**S1415CD** Follow-up Patient Survey		X					
**S1415CD** Use and Copayment Survey				X [3]			
**SITE COMPLETED FORMS**							
**S1415CD** Registration Worksheet [4]	X						
**S1415CD** Onstudy Form	X						
**S1415CD** Baseline Laboratory Values Form [1]	X						
**S1415CD** Cover Sheet for Patient-CompletedQuestionnaires	X	X		X[3]			
**S1415CD** CSF and Cancer Treatment Form		X					
**S1415CD** CSF Adverse Event Form		X[5]					
**S1415CD** Febrile Neutropenia Form		X					
**S1415CD** Antibiotics Log			X				
**S1415CD** 6 Month Febrile Neutropenia Log				X			
**S1415CD** 6 Month Status Update [2]				X			
**S1415CD** 12 Month Status Update [2]					X		
**S1415CD** Discontinuation of Initial SystemicTherapy [2]						X	
Notice of Death							X
**S1415CD** Cause of Death Supplement							X

1. Laboratory tests are not directed by this protocol, however; ANY laboratory results that have been obtained within 28 days prior to registration should be submitted on the **S1415CD** Baseline Laboratories Values Form. Medical records should be closely inspected for baseline leukocytes, ANC, peripheral platelet count and hemoglobin as they are of particular interest to this study.
2. Patients are followed for 12 months, regardless of whether they have discontinued the initial treatment regimen.
3. This survey is due by Month 6, but may be submitted whenever the patient has received the necessary documentation to complete this form. The **S1415CD** Cover Sheet for Patient-Completed Questionnaires should be submitted at the same time the **S1415CD** Use and Copayment Survey is submitted per Section 15.2f.
4. Complete this form prior to patient registration per Section 13.1, but do not submit this form.
5. The CSF Adverse Event Form is required only for those patients who receive CSF during first cycle of initial systemic therapy.

NOTE: Forms are found on the protocol abstract page on the SWOG website (www.swog.org). Forms submission guidelines are found in Section 14.0.

10.0 CRITERIA FOR EVALUATION AND ENDPOINT ANALYSIS

10.1 Primary Endpoints

a. Incidence of Febrile Neutropenia (clinical) within six months: Febrile neutropenia as defined by the NCI Common Terminology Criteria for Adverse Events (CTCAE) Version 4.0: absolute neutrophil count (ANC) < 1,000/uL and a single temperature of > 38.3 degrees C (101ºF) or a sustained temperature of ≥ 38ºC (100.4ºF) for more than one hour. (6) Collected at the end of the first cycle on the**S1415CD** Febrile Neutropenia Form and again at six months on the **S1415CD** 6 Month Febrile Neutropenia Log.

b. Rate of CSF prescribing as primary prophylaxis (clinical) during the first cycle of systemic therapy: Primary prophylaxis of CSF (PP-CSF) is defined as the initiation of granulocyte CSFs during the first cycle of myelosuppressive systemictherapy, given 24 to 72 hours after cessation of systemic therapy, as captured onthe **S1415CD** CSF and Cancer Treatment form.

c. Rate of FN-related emergency department (ED) visits and hospitalizations (clinical) during the first cycle: Admission to ED or hospital with documentation of fever and decreased ANC (see FN definition above), as recorded on the **S1415CD** Febrile Neutropenia form.

d. Overall survival (clinical): Time from date of registration to date of death due to any cause, using data from the **S1415CD** CSF and Cancer Treatment form, **S1415CD** 6 Month Status Update, **S1415CD** 12 Month Status Update, **S1415CD** Discontinuation of Initial Systemic Therapy form, and the Notice of Death. Patients who are lost to follow-up will be censored.

e. Prophylactic and FN-related antibiotic use during the first month of therapy (clinical): Outcomes will be measured as total number of antibiotic agents used and duration of antibiotic use, as collected on the **S1415CD** Febrile Neutropenia Form and the **S1415CD** 6 Month Febrile Neutropenia Log.

f. FN-related HRQL (patient report): Functional Assessment of Cancer Therapy - Febrile Neutropenia (Version 4) (FACT-N). Includes FACT general cancer score and FN-related score. (41,42,43) Evaluated at baseline and at the end of the first cycle of systemic therapy.

g. Patient adherence to PP-CSF prescription (clinical and patient report) at the end of the first cycle of systemic therapy: Proportion receiving PP-CSF among those for whom PP-CSF was ordered by the physician. The primary adherence measure will be obtained from the patient's chart as reported on the **S1415CD** CSF and Cancer Treatment form; secondary adherence will be measured via self-report on the **S1415CD** Follow-Up Patient Survey.

h. Patient knowledge of PP-CSF indications, risks, benefits, and out-of-pocket costs(patient report): Evaluated using a patient survey capturing information on PP- CSF that is relevant to informed decision-making (i.e., knowledge of potential benefits, side effects, and short and long-term potential harms, out of pocket costs, and alternative approaches to therapy). Patient knowledge will be evaluated at baseline and at the end of the first cycle of systemic therapy using the **S1415CD** Baseline Patient Survey and the **S1415CD** Follow-Up Patient Survey. Out of pocket costs will be assessed by 6 months after registration based on self-report using the **S1415CD** Use and Copayment Survey.

SWOG Authorized Document

i. Proportion completing initial systemic therapy regimen: a) at planned duration; b) at planned dose intensity (clinical): Records of early discontinuation or dose modification relative to the initially prescribed regimen will be captured on the **S1415CD** Discontinuation of Initial Systemic Therapy form and the **S1415CD** 12 Month Status Update.

10.2 Other Endpoints

a. Cancer relapse (clinical): Time from registration to documented invasive local or regional recurrence will be assessed using the **S1415CD** 6 Month Status Update and the **S1415CD** 12 Month Status Update. Invasive recurrence includes local, regional, or distant recurrence with histological confirmation preferred. Applies only to patients with local or regional disease treated with curative intent. Relapse will not be assessed for persons with metastatic disease at diagnosis. This endpoint will be monitored because it may affect the likelihood of allowing a full dose of systemic therapy, but is not tied to a formal hypothesis because ofthe small expected number of events.

10.3 Performance Status:

Patients will be graded according to the Zubrod Performance Status Scale.POINT

DESCRIPTION

0 Fully active, able to carry on all pre-disease performance without restriction.

1 Restricted in physically strenuous activity but ambulatory and able to carry out work of a light or sedentary nature, e.g., light housework, office work.

2 Ambulatory and capable of self-care but unable to carry out any work activities; up and about more than 50% of waking hours.

3 Capable of limited self-care, confined to bed or chair more than 50% of waking hours.

4 Completely disabled; cannot carry on any self-care; totally confined to bed or chair.

11.0 STATISTICAL CONSIDERATIONS

11.1 Estimate of Sample Size

Because FN events are less common in the intermediate risk group, we calculated sample size by taking a 2-step approach, working backwards from Primary Objective c to Primary Objective b and then demonstrating that Primary Objective a will have adequate power. For Primary Objective c, we fixed power at 80% and the number of components at24 (12 for PP-CSF, 12 for no PP-CSF). For these parameters, we obtained the sample size required in the Intervention arm to test the primary hypothesis for Primary Objective c: a 50% reduction in FN occurrence between the experimental (PP-CSF) and control (noPP-CSF) arms among patients receiving intermediate risk chemotherapy (measured by the odds ratio). Using this sample size, we determined the number of components needed in the Usual Care arm to give us at least 90% power to detect a 50% reduction inPrimary Objective b.

Step 1: For Primary Objective c, data used to inform the sample size calculations include:the risk of FN among control patients observed in retrospective studies (15%); and the

relative proportions of patients with breast, lung, and colorectal cancer receiving intermediate risk chemotherapy. *(44,45,46,47,48)* The null hypothesis used for determining sample size is that there will be no difference in FN incidence within 6months from registration (i.e., initiation of chemotherapy) between intervention and control groups.

Sample size estimates for the intermediate risk group are based on a 50% reduction in the FN rate (the reduction observed in trials for high risk settings). This translates to a reduction in FN rate from 15% to 7.5%, when comparing intermediate risk control arm patients (no PP-CSF) to intermediate risk intervention arm patients (PP-CSF). We believethis is reasonable because the one prospective trial evaluating PP-CSF for intermediate risk chemotherapy given to women with breast cancer achieved a risk reduction of 90%, and a systematic review of clinical trials of PP-CSF use in several settings found an FN rate reduction ranging from 17% to 78%. *(49)* To account for possible correlation between patients within components we estimate an intraclass correlation coefficient (ICC) of 0.02. An ICC of zero corresponds to randomization of individuals rather than components; ICC>0 indicates patient outcomes are more similar within a component thanacross components. We estimated the ICC for FN in the SWOG adjuvant breast cancer study **S0221** which has 262 clinics with 2,666 patients. *(50)* Using a random effects logistic regression program (component is the random effect) followed by numerical integration, the ICC was estimated to be 0.01 although statistically significantly greater than zero. We are using 0.02 to protect against underestimation of the ICC. Based on a two-sided 0.05 alpha level test, 24 components in the Intervention arm (12 components randomized to PP-CSF and 12 components to no PP-CSF among intermediate risk patients) and 45 intermediate-risk patients per component on average will give 80% power to detect whether PP-CSF reduces FN rate by 50% among patients receiving intermediate risk chemotherapy. We assume that high, intermediate and low risk patients comprise 30%, 50% and 20% at each component, respectively; this translates to a totalof 90 patients per component on average. *(51,52)*

Step 2: Consider the hypothesis of Primary Objective b: a relative reduction in FN occurrence over 6 months from registration between the Intervention and Usual Care arms (measured by the odds ratio) over both high and intermediate risk groups. We assume FN risk of 25% for the high risk chemotherapy group (translating to a reduction inFN rate from 25% to 12.5%). Using the sample size calculations from Step 1, we employa 3:1 randomization scheme in the main trial, resulting in 8 components randomized to Usual Care and 24 components randomized to Intervention. This translates to a total of 2,880 patients, 32 components (90 patients per component on average) for the study, and gives us 90% power for Primary Objective b: to detect a 50% reduction in FN events among high and intermediate risk patients in components randomized to the guideline- based PP-CSF order Intervention vs. Usual Care. **Figure 1** displays a power curve forour first and second Primary Objectives.

SWOG Authorized Document

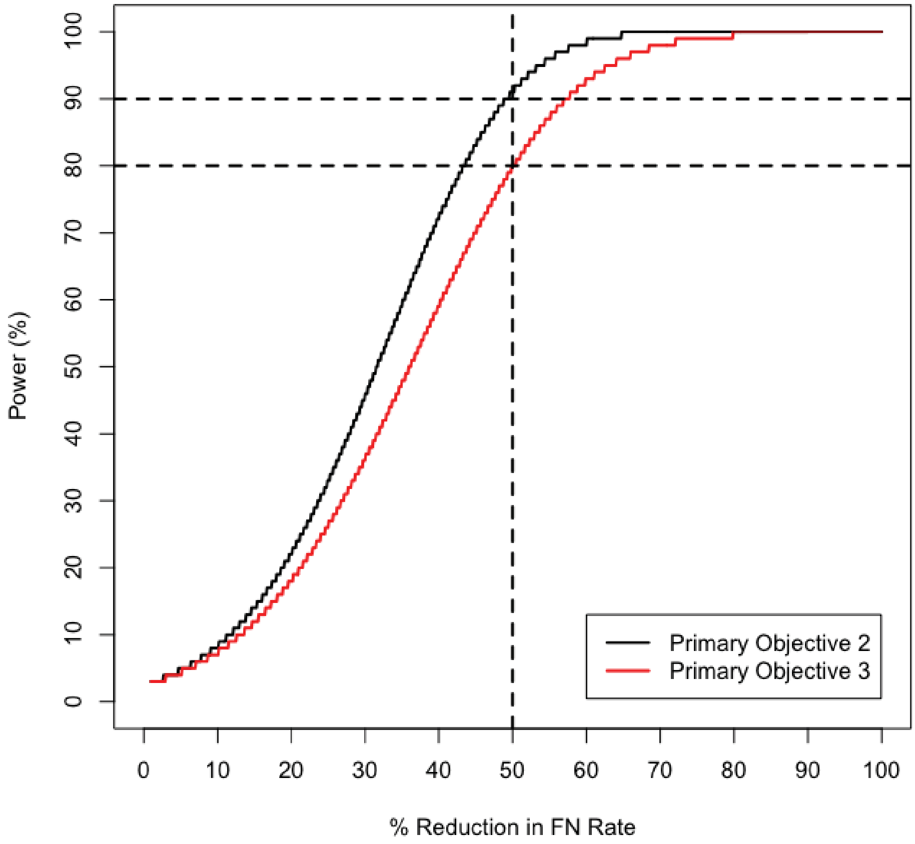

Figure 1: Power with respect to the reduction in FN rate. Thedashed vertical line is at 50% reduction. The two dashed horizontal lines are at 80% and 90% power.

Table 2 below illustrates how the required sample size for Primary Objective c and corresponding power for Primary Objective a vary according to the ICC.

Table 2. Power to detect 50% relative reduction in the FN rate. Power depends oncontrol arm event rate and ICC.

Intra-Class Correlation	Total Sample Size	Per arm sample size		Per arm # of components		Per component sample size		Power	
		Usual Care	Order Entry	Usual Care	Order Entry	Usual Care	Order Entry	Primary Obj b	Primary Obj a
0	1536	384	1152	8	24	48	48	80%	98%
.01	1984	496	1488	8	24	62	62	80%	94%
**.02**	**2880**	**720**	**2160**	**8**	**24**	**90**	**90**	**80%**	**90%**
.03	5376	1344	4032	8	24	168	168	80%	86%

Using this sample size and with greater than 80% power, we can detect the following effect of Intervention versus Usual Care in the process outcomes of Primary Objective a, namely the proportions of patients using CSF as primary prophylaxis versus other use: areduction in CSF use from 17% to 7% among low risk patients and an increase in CSF use from 40% to 75% among high-risk patients (based on observed use in our prior observational study). *(53)*

SWOG Authorized Document

In addition to the 32 study components, we will include 13 components with existing PP- CSF order entry systems in the parallel cohort to recruit 720 patients (56 patients per component on average).

A total of 3,600 eligible patients will be included in the study over a 2.75 year accrual period: 2,160 for the Intervention arm; 720 for the Usual Care arm; and 720 for the Cohort. We will allow for 10% additional accrual for a total of 3,960 patients. On average, this translates to 99 patients per component in the RCT, and 60 patients per componentin the Cohort. We will allow the maximum total number of patients per site to be flexible (i.e. not capping accrual at 99 patients per site), in order to allow high-accruing sites to compensate for low-accruing sites, and ultimately allow us to reach our total required sample size of 3,960 patients for the study. This total will account for ineligible patients,as well as dropout and death (not due to FN) that may occur earlier than 6 months after registration and therefore act as a competing event with FN occurrence. Additionally, we will monitor accrual to ensure that our assumed distribution of 30% high, 50% intermediate and 20% low risk patients in the overall sample across all components is roughly maintained. Major deviations from this assumption could result in a loss of power,and therefore if such deviations are observed, we will consider capping accrual byregimen risk.

11.2 Analysis of Primary Objectives

Primary prophylactic CSF (PP-CSF) is defined as CSF administered with the first cycle ofsystemic therapy, within 24 to 72 hours after the initial therapy dose. PP-CSF use is observed and reported.

The natural history of FN is cycle specific: a patient can develop FN within 3 to 10 days after the initial administration of chemotherapy, when their neutrophil count reaches its nadir. Therefore initial data collection is generally based on cycles rather than calendar time.

This study uses a cluster randomized design: the study intervention is administered at thecomponent level, rather than the patient level. The study intervention is to change the component's standing order system to reflect guidelines-based PP-CSF use, or to not change to the order system. All patients at a given component will be subject to the same order system, regardless of study participation; only registered patients will have their data reported to the study. Treating physicians at each component may change any individual patient's orders at any time, at their discretion.

The initial treatment regimen is defined as the systemic treatment intended for cancer treatment, at registration as determined by the treating physician and as defined in Section 18.1. Patients are followed through 12 months after registration, regardless of treatment status. This protocol does not direct systemic cancer treatment therapy.

Given the cluster randomization design, we will estimate the intraclass correlation coefficient (ICC) and adjust for it in all analyses.

a. *Primary Objective a: To compare the use of primary prophylactic colony stimulating factor (PP-CSF) according to recommended clinical practice guidelines between patients registered at Intervention components versus Usual Care components.*

PP-CSF is by definition administered at the start of the first cycle of chemotherapy; this objective will evaluate the use of CSF during the first cycle of systemic therapy.

Randomization is at the component level; outcomes are measured at the patient level. Since patients are nested within components, this structure will be

SWOG Authorized Document

accounted for by using mixed effects regression models including a random effect for component. Among high risk and low risk patients, separate mixed effects logistic models will be fit to assess the effect of the intervention on PP- CSF use. In addition to including a random effect for component and an indicator variable for randomized group (Intervention versus Usual Care), we will adjust forcomponent-level characteristics and patient-level clinical and demographic variables.

If the quality of the component application data is acceptable (proportion of missingness ≤ 10%), we will descriptively compare trends in PP-CSF prescribing prior to the start of the study versus after the start of the study, in order to determine if there is a change in PP-CSF prescribing at the components basedon their participation in the study (i.e., a Hawthorne effect).

b. *Primary Objective b: To compare the rate of febrile neutropenia (FN) between patients registered to Intervention components versus Usual Care components.*

The rate of FN will be evaluated at 6 months post-registration. The risk of FN is greatest during the first cycle, but patients are still at risk throughout therapy. TheFN risk levels assigned to the various systemic therapies in this protocol are based on risk for the duration of treatment, and so the analysis will look at the FNrate at 6 months into therapy.

Randomization is at the component level; outcomes are measured at the patient level. Since patients are nested within components, this structure will be accounted for by using mixed effects regression models including a random effect for component. A mixed effects logistic model will be used to assess the effect of the intervention on FN occurrence within 6 months from registration. In addition to including an indicator variable for randomized group (Intervention versus Usual Care), we will adjust for component-level characteristics and patient-level clinical and demographic variables. We will also descriptively compare time to FN occurrence between Intervention and Usual Care practices using Kaplan-Meier plots.

In a secondary analysis, we will do a three-way comparison among the intermediate risk patients, comparing Usual Care to each of the two Intervention groups – PP-CSF and no PP-CSF. For this analysis, we will fit a similar model asabove, but instead of an indicator variable for randomized group (Intervention versus Usual Care), we will include a categorical variable for randomized group with three levels: (i) Usual Care (reference), (ii) Intervention – PP-CSF, and (iii) Intervention – no PP-CSF.

Subgroup analysis: We will assess the magnitude of intervention effect in subgroups and how that effect changes across subgroups (after stratification by subgroup). We will use interaction tests and construct a forest plot to display the heterogeneity in intervention effects across subgroups (details in Section 11.7). Subgroup analyses will be performed for each cancer type, level of FN risk, race and the following variables: race (African-American, white, other); age (categorized as < 50, 50-59, 60-69, 70-79, 80+ years); non-cancer comorbidity performance status (Zubrod), stage of cancer at enrollment (distant, local, regional). *(54,55,56,57,58,59)*

c. *Primary Objective c: To compare the rate of FN among intermediate risk patients registered to Intervention components by component treatment assignment (administer PP-CSF to intermediate risk patients versus not).*

The rate of FN will be evaluated at 6 months post-registration. The risk of FN is greatest during the first cycle, but patients are still at risk throughout therapy. TheFN risk levels assigned to the various systemic therapies in this protocol are based on risk for the duration of treatment, and so the analysis will look at the FNrate at 6 months into therapy.

As before, since patients are nested within components, a mixed effects logistic model with a random effect for component will be used to assess the effect of prescribing CSFs as primary prophylaxis on FN occurrence within 6 months from registration. In addition to including an indicator variable for randomized group (CSFs prescribed as primary prophylaxis or not), we will adjust for component- level characteristics and patient-level clinical and demographic variables.

Subgroup Analysis: Among intermediate risk patients registered to Intervention components, we will assess the magnitude of PP-CSF effect in subgroups defined by FN risk factors and how that effect changes across subgroups (after stratification by subgroup). We will use interaction tests and construct a forestplot to display the heterogeneity in PP-CSF effects across subgroups (details in Section 11.7). Subgroup analyses will be performed for the following risk factors: age (categorized as < 50, 50-59, 60-69, 70-79, 80+ years), race, comorbidconditions, poor performance status, prior chemotherapy, serum albumin abnormal liver function, poor renal function, low baseline WBC, chemotherapy dose intensity, type of chemotherapy.

11.3 Analysis of Secondary Objectives

a. *Secondary Objective a: To compare the rate of FN between low-risk patients registered at Intervention components versus Usual Care components.*

The rate of FN will be evaluated at 6 months post-registration. The risk of FN is greatest during the first cycle, but patients are still at risk throughout therapy. TheFN risk levels assigned to the various systemic therapies in this protocol are based on risk for the duration of treatment, and so the analysis will look at the FNrate at 6 months into therapy.

A mixed effects logistic model with a random effect for component will be used toassess the effect of prescribing CSFs as primary prophylaxis on FN occurrence within 6 months from registration among low-risk patients. In addition to includingan indicator variable for component assignment (Intervention versus Usual Care),we will adjust for component-level characteristics and patient-level clinical and demographic variables.

b. *Secondary Objective b: To compare the FN-related health-related quality of life (HRQL) between low-risk patients registered at Intervention components versus Usual Care components.*

HRQL is evaluated at the end of the first cycle, the target period for the intervention, while accounting for baseline.

A linear mixed effects model will be fit to assess the effect of the intervention on HRQL, treating the FACT-N score at the follow-up clinic visit as the outcome and adjusting for the baseline FACT-N score. In addition to including an indicator variable for randomized group and a random effect for component, we will adjust for component-level characteristics and patient-level clinical and demographic variables.

c. *Secondary Objective c: To compare patient adherence to PP-CSF prescribing between patients registered at Intervention components versus Usual Care components.*

SWOG Authorized Document

PP-CSF is only prescribed during the first cycle, so patient adherence to PP-CSFwill be evaluated during the first cycle.

For the home and clinic settings, separate mixed effects logistic models will be used to assess the effect of the intervention on adherence to PP-CSF orders, treating adherence after the start of the study as the outcome. In addition to including an indicator variable for randomized group and a random effect for component, we will adjust for component-level characteristics and patient-level clinical and demographic variables.

d. *Secondary Objective d: To compare patient knowledge of the indications for, efficacy of, and side effects associated with PP-CSF between the initiation and conclusion of the first cycle of myelosuppressive systemic therapy between patients registered at Intervention components versus Usual Care components.*

PP-CSF is only prescribed during the first cycle, so patient knowledge of PP-CSFwill be evaluated as the change in knowledge between baseline and the end of the first cycle.

We will analyze change in the patient knowledge score between the initiation and conclusion of the first cycle of myelosuppressive systemic therapy, by fitting a linear mixed effects model with a time variable and an interaction betweenrandomized group and time. We will use random effects to accommodate both the correlation among measures from the same patient as well as the correlation among patients from the same component. We will also adjust for component- level characteristics and patient-level clinical and demographic variables.

e. *Secondary Objective e: To compare the proportion of patients completing the initial systemic therapy regimen at planned duration and at planned dose intensity between patients registered at Intervention components versus Usual Care components.*

Patients are followed for 12 months; therapy status will be determined by 12 months.

Two separate mixed effects logistic models will be used to assess the effect of the intervention on completion of the initial systemic therapy regimen (i) at planned duration and (ii) at planned dose intensity. In addition to including an indicator variable for randomized group and a random effect for component, we will adjust for component- level characteristics and patient-level clinical and demographic variables.

f. *Secondary Objective f: To compare antibiotic use both as prophylaxis and as treatment for FN between patients registered at Intervention components versus Usual Care components.*

Antibiotic use as prophylaxis for FN and as a treatment for FN is relevant during the first cycle when PP-CSF may be administered. To adequately cover allantibiotic use that may be related to prophylaxis and treatment of FN, antibiotic prescriptions for the first 30 days of systemic treatment will be evaluated.

A linear mixed effects model will be fit to assess the effect of the intervention on duration of antibiotics use with number of days as the outcome. Mixed effects Poisson models will be used to assess the effect of the intervention on total number of antibiotics agents used. Three separate models will be fit, with the following outcomes: (i) the number of times antibiotics were used as prophylaxis,
(ii) the number of times antibiotics were used as treatment for FN, and (iii) the

total number of times antibiotics were used. In addition to including an indicator variable for randomized group and a random effect for component, we will adjust for component-level characteristics and patient-level clinical and demographic variables. Robust variance estimation will be used to relax the strongassumptions about the variance made by Poisson regression. If a large numberof zero counts is observed, then zero-inflated Poisson regression will be used.

g. *Secondary Objective g: To compare the rate of FN-related emergencydepartment visits and hospitalizations among intermediate risk patients registered to Intervention components by component treatment assignment (administer PP-CSF to intermediate risk patients versus not).*

Hospitalizations and ED visits will be evaluated at the end of the first cycle, when patients are at greatest risk for FN and thus more likely to have a hospitalization and/or ED visit for FN.

A mixed effects Poisson model will be used to assess the effect of the intervention on FN-related ED visits and hospitalizations, stated as a composite endpoint because some patients are directly admitted from clinic to hospital, bypassing the ED. In addition to including an indicator variable for randomized group and a random effect for component, we will adjust for component-level characteristics and patient-level clinical and demographic variables. We will account for variable lengths of follow-up across patients due to varying lengths of the initial regimen by including an offset term in the model. Robust variance estimation will be used to relax the strong assumptions about the variance made by Poisson regression. If a large number of zero counts is observed, then zero- inflated Poisson regression will be used.

h. *Secondary Objective h: To compare the FN-related health-related quality of life (HRQL) patients among intermediate risk patients registered to Intervention components by component treatment assignment (administer PP-CSF to intermediate risk patients versus not).*

HRQL is evaluated at the end of the first cycle, the target period for the intervention, while accounting for baseline.

A linear mixed effects model will be fit to assess the effect of the intervention on HRQL, treating the FACT-N score at the follow-up clinic visit as the outcome and adjusting for the baseline FACT-N score. In addition to including an indicator variable for randomized group and a random effect for component, we will adjust for component-level characteristics and patient-level clinical and demographic variables

i. *Secondary Objective i: To compare overall survival among intermediate risk patients registered to Intervention components by component treatment assignment (administer PP-CSF to intermediate risk patients versus not).*

Patients are followed for 12 months; survival status will be determined by 12 months.

A Cox proportional hazards model will be used to model survival. The outcome will be overall survival measured in days since trial entry to 1 year following entry.In addition to including an indicator variable for randomized group, we will adjust for component-level characteristics and patient-level clinical and demographic variables. A separate analysis of cause-specific survival to address FN-related deaths will also be conducted.

SWOG Authorized Document

11.4 Analysis of Additional Objectives

a. *Additional Objective a: To characterize and descriptively report the differences between Cohort components (components with pre-existing guideline-based order entry systems) to the Intervention and Usual Care components, according to the endpoints outlined in Section 10.0.*

Endpoints will be evaluated at the time points described in Sections 11.2 and 11.3.

We will characterize and descriptively report all study outcomes in the Cohort components in comparison with the Intervention and Usual Care components. We will evaluate all outcomes, namely adherence to recommendations, clinical outcomes (e.g., FN rates, FN-related hospitalizations) and patient-reported outcomes. As such, this analysis will be exploratory and comparative.

b. *Additional Objective b: To evaluate the time to invasive recurrence in non-metastatic patients by component treatment assignment.*

Patients are followed for 12 months; time to invasive recurrence will be determined by 12 months.

Time from registration to documented invasive local or regional recurrence. Invasive recurrence includes local, regional, or distant recurrence with histological confirmation preferred. Applies only to patients with local or regional disease treated with curative intent. Relapse will not be assessed for persons with metastatic disease at diagnosis. This endpoint will be monitored because it may affect the likelihood of allowing a full dose of systemic therapy, but is not tiedto a formal hypothesis because of the small expected number of events. We will summarize this endpoint by calculating cumulative incidence over time.

11.5 Intent-to-Treat Analysis

There may be non-compliance at the component level in the primary randomization andat the component or patient level in the secondary randomization. The primary analysis will be an intent-to-treat (ITT) analysis, which will disregard non-compliance and analyze patients and components according to the groups to which they were randomly assigned.As a sensitivity analysis, we will account for non-compliance and conduct an as-treated analysis. In order to understand any differences between results from the ITT and as- treated analyses and to assess the extent to which crossover components and patients may have biased results, we will compare their characteristics to those of all other components and patients in the analysis.

11.6 Missing Data

SWOG has established procedures to minimize missing data. If the data are not reported at the expected time, a query is generated in the electronic data capture (EDC) system (iMedidata Rave) and the clinic CRA must respond to the missing data request. In the rare situation that the outcome data remain missing, it most likely indicates that a patient did not have a PP-CSF prescription and did not have a FN outcome (otherwise accurate clinical reporting is not occurring). Accordingly, the patient will be assigned a zero for thisoutcome. While one could consider imputation methods that would then assign a 0 or 1 to the patient with some probability, we believe that could be misleading. Since this is an intent-to-treat analysis it is important that each patient be represented. A secondary analysis will exclude those patients without a known outcome. Assignment of a zero is non-differential by treatment group and would be conservative with respect to testing for

SWOG Authorized Document

a treatment difference. It could underestimate slightly the proportion of patients who had a PP-CSF prescription or a FN outcome. Nonetheless, in medical record review one would never assume that a prescription was given or an outcome occurred without support in the medical record. Any secondary analyses that show significant variation with the primary analysis due to missing values will be reported in the manuscript. Additionally, we will include a comparison of the baseline characteristics of patients with and without missing data.

Any patients who drop out have a note recorded in the database for the reason. These are reported in the Consolidated Standards of Reporting Trials (CONSORT) figure included with every clinical trial report. All participants included in the study will be accounted for in the report, whether or not they are included in the analysis.

11.7 Heterogeneity of Treatment Effect

Heterogeneity of treatment effect is routinely shown using interaction tests and forest plots of the odds ratio (or hazard ratio) and 95% CI for each relevant subgroup. For the primary outcomes, we will use forest plots to assess the magnitude of intervention effect in subgroups (after stratification) and how that effect changes across subgroups. The interaction of intervention and each factor will also be tested in the model after the main effects have been included. The scientific plausibility of that subset effect will be assessed only if there is evidence of statistically significant interaction. In the absence ofa significant main effect, it is likely that most effects in a subset are spurious and should not be regarded as meaningful. If there is a significant overall main effect, but certain subgroups do not share that effect as evidenced by an odds ratio close to unity that mightsuggest heterogeneity. Interest lies in interactions that may result in different treatment decisions for different subgroups. This translates to a greater interest in qualitativeinteractions (treatment effects among subgroups differ in direction) than quantitative interactions (treatment effects differ in magnitude but not in direction). While a significant interaction shows if subgroups are different from each other, it could miss qualitative interactions. Forest plots show the treatment effect estimates and variability within each subgroup. They will be used as a complementary visual tool for assessing both qualitative and quantitative interactions.

We expect the relative effect (but not the absolute effect) of Intervention versus Usual Care to be the same in high and intermediate patients. This will be tested by an interaction term, but there is already a separate planned analysis for the intermediate group alone that has 80% power. Additionally, there is a planned analysis of CSF use as primary prophylaxis in the high and low risk groups that has >80% power. No other a priori hypotheses are stated, and therefore all other subgroup analyses are exploratory.

11.8 Data and Safety Monitoring

A Data and Safety Monitoring Committee will oversee the conduct of the study (see SWOG Policy #21). The Committee consists of four members from outside of the SWOG, 3 SWOG members, 3 non-voting representatives from the National CancerInstitute (NCI), and the Group Statistician (non-voting). The members of this Committee will receive confidential reports every 6 months from the SWOG Statistical Center, and will meet at the Group's bi-annual meetings as necessary. The Committee will be responsible for recommendations regarding possible termination and/or early reporting ofthe study.

11.9 Interim Analysis

An interim analysis for Primary Objective c will be conducted. This may allow early termination of part of the sub-trial (e.g. a single arm) in intermediate risk patients due to demonstrated efficacy or futility of PP-CSF in an interim analysis of FN incidence. Interim

SWOG Authorized Document

analyses will be conducted when 360 patients (24 clinics * 15 patients/per clinic) and 650 patients (24 clinics * 27 patients/per clinic) in the intermediate risk group at Intervention clinics have complete outcome information, i.e., their outcomes have been observed and classified as positive or negative; these sample sizes correspond to 50% and 75% information. Information is calculated based on the ICC of 0.02, and therefore it accumulates faster than does patient accrual. We will use O'Brien-Fleming stopping rules, which employ conservative criteria for termination and result in a minimal loss of power. Pre-specified decision criteria will be as follows: for 80% power, the cumulative alpha spent at the 50%, 75%, and final time points will be 0.003, 0.019, and 0.050, respectively. The corresponding p-value cutoffs for frequentist inference will be 0.003, 0.018, and 0.044, respectively. Futility analysis will also be conducted at the same time points. If the p-value is >0.936, >0.292 or >0.044, respectively, or if the odds ratio is inthe opposite direction as that hypothesized, consideration will be given to declaring futility.

The interim analyses will be reported to the DSMC, who will make a recommendation to continue or stop the sub-trial. If early termination of the sub-trial for Primary Objective c occurs, the DSMC will be consulted about next steps regarding accrual to meet sample size requirements for Primary Objectives a and b.

12.0 DISCIPLINE REVIEW

Discipline review is not necessary for this study.

13.0 REGISTRATION GUIDELINES

13.1 Registration Timing

Components must have received approval from the **S1415CD** Study Team before registering patients to the study (see Section 15.1).

Patients must be registered prior to initiation of study-allowed therapy (no more than 5 working days prior to planned start of initial systemic therapy).

13.2 Investigator/Site Registration Procedures

Prior to the recruitment of a patient for this study, investigators must be registered members of a Cooperative Group. Each investigator must have an NCI investigator number and must maintain an "active" investigator registration status through the annual submission of a complete investigator registration packet to CTEP.

13.3 CTEP Registration Procedures

Food and Drug Administration (FDA) regulations and National Cancer Institute (NCI) policy require all individuals contributing to NCI-sponsored clinical trials to register and to renew their registration annually. To register, all individuals must obtain a CancerTherapy Evaluation Program (CTEP) Identity and Access Management (IAM) account at https://ctepcore.nci.nih.gov/iam. In addition, persons with a registration type of Investigator (IVR), Non-Physician Investigator (NPIVR), or Associate Plus (AP) (i.e.,clinical site staff requiring write access to OPEN, Rave, or acting as a primary site contact) must complete their annual registration using CTEP's web-based Registration and Credential Repository (RCR) (https://ctepcore.nci.nih.gov/rcr).

RCR utilizes five-person registration types.

- IVR — MD, DO, or international equivalent;

- NPIVR — advanced practice providers (e.g., NP or PA) or graduate level researchers(e.g., PhD);
- AP — clinical site staff (e.g., RN or CRA) with data entry access to CTSUapplications (e.g., Roster Update Management System (RUMS), OPEN, Rave,);
- Associate (A) — other clinical site staff involved in the conduct of NCI-sponsoredtrials; and
- Associate Basic (AB) — individuals (e.g., pharmaceutical company employees) withlimited access to NCI-supported systems.

RCR requires the following registration documents.

**Documentation Required**	**IVR**	**NPIVR**	**AP**	**A**	**AB**
FDA Form 1572	☐	☐			
Financial Disclosure Form	☐	☐	☐		
NCI Biosketch (education, training, employment, license, and certification)	☐	☐	☐		
GCP training	☐	☐	☐		
Agent Shipment Form (if applicable	☐				
CV (optional)	☐	☐	☐		

An active CTEP-IAM user account and appropriate RCR registration is required to accessall CTEP and Cancer Trials Support Unit (CTSU) websites and applications. In addition, IVRs and NPIVRs must list all clinical practice sites and Institutional Review Boards (IRBs) covering their practice sites on the FDA Form 1572 in RCR to allow the following:

- Addition to a site roster;
- Assign the treating, credit, consenting, or drug shipment (IVR only) tasks in OPEN;
- Act as the site-protocol Principal Investigator (PI) on the IRB approval; and
- Assign the Clinical Investigator (CI) role on the Delegation of Tasks Log (DTL).

In addition, all investigators act as the Site-Protocol PI, consenting/treating/drug shipment, or as the CI on the DTL must be rostered at the enrolling site with a participating organization (i.e., Alliance).

Additional information is located on the CTEP website at https://ctep.cancer.gov/investigatorResources/default.htm. For questions, please contact the **RCR Help Desk** by email at RCRHelpDesk@nih.gov.

13.4 CTSU Registration Procedures

This study is supported by the NCI Cancer Trials Support Unit (CTSU).

a. **IRB Approval**

Sites using their local IRB or REB, must submit their approval to the CTSU Regulatory Office using the Regulatory Submission Portal located in the Regulatory section of the CTSU website. Acceptable documentation of local IRB/REB approval includes:

- Local IRB documentation;
- IRB-signed CTSU IRB Certification Form; and/or
- Protocol of Human Subjects Assurance Identification/IRB Certification/Declaration of Exemption Form.

In addition, the Site-Protocol Principal Investigator (PI) (i.e. the investigator on the IRB/REB approval) must meet the following criteria to complete processing of the IRB/REB approval record:

- Holds an Active CTEP status;
- Rostered at the site on the IRB/REB approval (*applies to US and Canadian sites only*) and on at least one participating roster;
- If using NCI CIRB, rostered on the NCI CIRB Signatory record;
- Includes the IRB number of the IRB providing approval in the Form FDA 1572 in the RCR profile; and
- Holds the appropriate CTEP registration type for the protocol.

Additional Requirements

Assignment of site registration status in the CTSU Regulatory Support System (RSS) uses extensive data to make a determination of whether a site has fulfilled all regulatory criteria including but not limited to: the following:

- An active Federal Wide Assurance (FWA) number;
- An active roster affiliation with the Lead Protocol Organization (LPO) or a Participating Organization (PO); and
- Compliance with all protocol-specific requirements (PSRs).

b. **Downloading Site Registration Documents**

Download the site registration forms from the protocol-specific page located on the CTSU members' website. Permission to view and download this protocol andits supporting documents is restricted and is based on person and site roster assignment housed in the CTSU Regulatory Support System (RSS). To participate, the institution and its associated investigators and staff must be associated with the LPO or a PO on the protocol.

- Log on to the CTSU members' website (https://www.ctsu.org) using your CTEP-IAM username and password;
- Click on *Protocols* in the upper left of your screen
  - o Enter the protocol number in the search field at the top of the protocol tree, or
  - o Click on the By Lead Organization folder to expand, then select *SWOG*, and protocol number ***S1415CD***.

Click on *Documents*, select *Site Registration*, and download and complete theforms provided.

c. **Requirements for S1415CD *Site* Registration**

- CTSU IRB Certification
- CTSU IRB/Regulatory Approval Transmittal Sheet

d. **Submitting Regulatory Documents**

Submit required forms and documents to the CTSU Regulatory Office via the Regulatory Submission Portal on the CTSU website.

To access the Regulatory Submission Portal log on to the CTSU members'website Regulatory Regulatory Submission.

Institutions with patients waiting that are unable to use the Regulatory Submission Portal should alert the CTSU Regulatory Office immediately at 1- 866-651-2878 in order to receive further instruction and support.

e. **Checking Your Site's Registration Status**

You can verify your site registration status on the members' side of the CTSU website.

- Log on to the CTSU members' website;
- Click on *Regulatory* at the top of your screen;
- Click on *Site Registration;*
- Enter your 5-character CTEP Institution Code and click on Go.

Note: The status shown only reflects institutional compliance with site registration requirements as outlined above. It does not reflect compliance with protocolrequirements for individuals participating on the protocol or the enrolling investigator's status with the NCI or their affiliated networks.

13.5 Oncology Patient Enrollment Network (OPEN) Registration Requirements

The individual registering the patient must have completed the appropriate SWOG Registration Worksheet. The completed form must be referred to during the registration but should not be submitted as part of the patient data.

The Oncology Patient Enrollment Network (OPEN) is a web-based registration system available on a 24/7 basis. OPEN is integrated with CTSU regulatory and roster data and with the Lead Protocol Organization (LPOs) registration/randomization systems or Theradex Interactive Web Response System (IWRS) for retrieval of patient registration/randomization assignment. OPEN will populate the patient enrollment data in NCI's clinical data management system, Medidata Rave.

Requirements for OPEN access:

- A valid CTEP-IAM account;
- To perform enrollments or request slot reservations: Be on a LPO roster, ETCTN Corresponding roster, or PO roster with the role of Registrar. Registrars must hold a minimum of an AP registration type;
- If a Delegation of Tasks Log (DTL) is required for the study, the registrar(s) must hold the OPEN Registrar task on the DTL for the site; and
- Have an approved site registration for a protocol prior to patient enrollment.

To assign an Investigator (IVR) or Non-Physician Investigator (NPIVR) as the treating, crediting, consenting, drug shipment (IVR only), or receiving investigator for a patient transfer in OPEN, the IVR or NPIVR must list the IRB number used on the site's IRB approval on their Form FDA 1572 in RCR. If a DTL is required for the study, the IVR or NPIVR must be assigned the appropriate OPEN-related tasks on the DTL.

Prior to accessing OPEN, site staff should verify the following:

- Patient has met all eligibility criteria within the protocol stated timeframes and the affirmation of eligibility on the Registration Worksheet has been signed by the registering investigator or another investigator designate. Site staff should referto Section 5.0 to verify eligibility.
- All patients have signed an appropriate consent form and HIPAA authorization form (if applicable).

SWOG Authorized Document

Note: The OPEN system will provide the site with a printable confirmation of registration and treatment information. Please print this confirmation for your records.

OPEN will also ask additional questions that are not present on the SWOG Registration Worksheet. The individual registering the patient must be prepared to provide answers to the following questions:

a. Institution CTEP ID

b. Protocol Number

c. Registration Step

d. Treating Investigator

e. Credit Investigator

f. Patient Initials

g. Patient’s Date of Birth

h. Patient SSN (SSN is desired, but optional. Do not enter invalid numbers.)

i. Country of Residence

j. ZIP Code

k. Gender (select one):
- Female Gender
- Male Gender

l. Ethnicity (select one):
- Hispanic or Latino
- Not Hispanic or Latino
- Unknown

m. Method of Payment (select one):
- Private Insurance
- Medicare
- Medicare and Private Insurance
- Medicaid
- Medicaid and Medicare
- Military or Veterans Sponsored NOS
- Military Sponsored (Including Champus & Tricare)
- Veterans Sponsored
- Self Pay (No Insurance)
- No Means of Payment (No Insurance)
- Other
- Unknown

n. Race (select all that apply):
- American Indian or Alaska Native
- Asian
- Black or African American
- Native Hawaiian or other Pacific Islander

SWOG Authorized Document

- White
- Unknown

All site staff will use OPEN to enroll patients to this study. Access OPEN at https://open.ctsu.org or from the OPEN link on the CTSU members' website. Further instructional information is in the OPEN section of the CTSU website at https://www.ctsu.org, https://open.ctsu.org, or from the OPEN Patient Registration link on the SWOG CRA Workbench. For any additional questions, contact the CTSU Help Desk at 1-888-823-5923 orctsucontact@westat.com.

13.6 Exceptions to SWOG registration policies will not be permitted.

a. Patients must meet all eligibility requirements.

b. Institutions must be identified as approved for registration.

c. Registrations may not be cancelled.

d. Late registrations (after the start date of treatment) will not be accepted.

14.0 DATA SUBMISSION SCHEDULE

14.1 Data Submission Requirement

Data must be submitted according to the protocol requirements for **ALL** patients registered, including patients deemed to be ineligible. Patients for whom documentation is inadequate to determine eligibility will generally be deemed ineligible.

14.2 Master Forms

Master forms can be found on the protocol specific web page of the CTSU Member Website located at https://www.ctsu.org or on the protocol abstract page on the SWOG website (www.swog.org) and (with the exception of the sample consent form and the Registration Worksheet) must be submitted on-line via the Web; see Section 14.3a for details.

14.3 Data Submission Procedures

a. Medidata Rave® is a clinical data management system being used for data collection for this trial/study. Access to the trial in Rave is controlled through the CTEP-IAM system and role assignments. To access Rave via iMedidata:

- Site staff will need to be registered with CTEP and have a valid and active CTEP-IAM account; and
- Assigned one of the following Rave roles on the relevant Lead Protocol Organization (LPO) or Participating Organization roster at the enrolling site: Rave CRA, Rave Read Only, Rave CRA (LabAdmin), Rave SLA, or Rave Investigator. Refer to https://ctep.cancer.gov/investigatorResources/default.htm for registration types and documentation required.
    - To hold Rave CRA or Rave CRA (Lab Admin) role, site staff must hold a minimum of an AP registration type;
    - To hold Rave Investigator role, the individual must be registered as an NPIVR or IVR; and
    - To hold Rave Read Only role, site staff must hold an Associates (A) registration type.

SWOG Authorized Document

If the study has a Delegation of Tasks Log (DTL), individuals requiring writeaccess to Rave must also be assigned the appropriate Rave tasks on the DTL.

Upon initial site registration approval for the study in Regulatory Support System (RSS), all persons with Rave roles assigned on the appropriate roster will be senta study invitation e-mail from iMedidata. To accept the invitation, site staff must log into the Select Login (https://login.imedidata.com/selectlogin) using their CTEP-IAM user name and password, and click on the "accept" link in the upper right-corner of the iMedidata page. Site staff will not be able to access the studyin Rave until all required Medidata and study specific trainings are completed. Trainings will be in the form of electronic learnings (eLearnings), and can be accessed by clicking on the link in the upper right pane of the iMedidata screen.If an eLearning is required and has not yet been taken, the link to the eLearning will appear under the study name in iMedidata instead of the Rave EDC link;once the successful completion of the eLearning has been recorded, access to the study in Rave will be granted, and a Rave EDC link will display under the study name.

Site staff that have not previously activated their iMedidata/Rave account at the time of initial registration approval for the study in RSS will also receive aseparate invitation from iMedidata to activate their account. Account activation instructions are located on the CTSU website, Rave tab under the Rave resourcematerials (Medidata Account Activation and Study Invitation Acceptance). Additional information on iMedidata/Rave is available on the CTSU members' website in the Data Management > Rave section at www.ctsu.org/RAVE/ or by contacting the CTSU Help Desk at 1-888-823-5923 or by e-mail at ctsucontact@westat.com.

b. You may also access Rave® via the SWOG CRA Workbench via the SWOGwebsite (www.swog.org).

For difficulties with the CRA Workbench, please email technicalquestion@crab.org.

c. Institutions participating through Cancer Trials Support Unit (CTSU) please referto the CTSU Participation Table.

14.4 Data Submission Overview and Timepoints

NOTE: Cycle length is determined by treatment regimen. Regimens that normally have a cycle length of fewer than 14 days will be reported as a 14-day cycle for the purposes of study data collection. Specifically, regimens with a cycle length of 1 day or 7 days will have a data collection at Day 14 for "end of first cycle" data, including patient completed questionnaires, using Day 14 as the Cycle End Date. See Section 18.1 for study-allowed regimens and regimen-specific cycle lengths.

a. WITHIN 7 DAYS AFTER REGISTRATION:

Submit the following:

S1415CD Onstudy Form

S1415CD Baseline Laboratory Values Form

S1415CD Cover Sheet for Patient-Completed Questionnaires

S1415CD Baseline Patient Survey

S1415CD FACT-N (Version 4)

S1415CD Medical Conditions Questionnaire

b. AT 1 MONTH (WITHIN 14 DAYS AFTER THE FIRST 30 DAYS OF INITIAL SYSTEMIC THERAPY):

Submit the **S1415CD** Antibiotics Log.*

* The data reported must cover the full 30 days.

c. WITHIN 14 DAYS AFTER THE PATIENT HAS COMPLETED FIRST CYCLE OF THERAPY:

Submit the following:

S1415CD CSF and Cancer Treatment Form

S1415CD CSF Adverse Event Form
(Required only if patient received CSF during first cycle of initial systemictherapy.)

S1415CD Febrile Neutropenia Form

S1415CD Cover Sheet for Patient-Completed Questionnaires

S1415CD Follow-Up Patient Survey

S1415CD FACT-N (Version 4)

d. AT 6 MONTHS (WITHIN 14 DAYS AFTER 6 MONTHS):

Submit the following:

S1415CD Cover Sheet for Patient-Completed Questionnaires

S1415CD Use and Copayment Survey (This survey is due by Month 6, but may be submitted whenever the patient has received the necessary documentation tocomplete this.)

S1415CD 6 Month Febrile Neutropenia Log *

S1415CD 6 Month Status Update *

* The data reported must cover the full 6 months.

e. AT 12 MONTHS (WITHIN 14 DAYS AFTER 12 MONTHS):

Submit the following:

S1415CD 12 Month Status Update *

* The data reported must cover the full 12 months.

SWOG Authorized Document

f. <u>WITHIN 14 DAYS OF KNOWLEDGE OF DISCONTINUATION OF INITIAL TREATMENT REGIMEN</u>:

Submit the following:

S1415CD Discontinuation of Initial Systemic Therapy

g. <u>WITHIN 4 WEEKS OF KNOWLEDGE OF DEATH</u>:

Submit the Notice of Death and the **S1415CD** Cause of Death Supplement documenting death information. Also submit the **S1415CD** 6 Month Status Update or **S1415CD** 12 Month Status Update (whichever is due next) and the **S1415CD** Discontinuation of Initial Systemic Therapy (if the patient was still on initial treatment regimen). Also, submit the **S1415CD** 6 Month Febrile Neutropenia Log if the patient died prior to 6 months on study.

NOTE: Forms required at time of death should report all data from date of registration up to date of death.

15.0 SPECIAL INSTRUCTIONS

15.1 Component Application, Review, and Participation

a. Application

The **S1415CD** Component Application is available on the protocol abstract page on the SWOG Website (www.swog.org) and the CTSU Member Website (https://www.ctsu.org). All interested SWOG, Alliance, NRG, and ECOG-ACRIN NCORP CCDR components must submit an application to be considered for participation. The purpose of the application is to verify that components will be able to fully meet the study requirements before attempting to open the trial. The application contains the component requirements and asks questions intended todetermine component eligibility.

b. Review

Submitted applications will be carefully reviewed by the **S1415CD** Study Team before a component will be approved to participate. If necessary, additional questions may be asked of individual components during the application review period to help the **S1415CD** Study Team determine the feasibility of the trial at the particular location.

The **S1415CD** Study Team will inform components whether they have been approved for participation.

c. Participation

If a component is unable to continue participation in the protocol at any timewhile it is still actively accruing, the component must notify the **S1415CD** Study Team at TrACER@fredhutch.org.

Components may begin recruiting to the trial after approval has been granted by the **S1415CD** Study Team and regulatory administration has been completed perSection 13.2 and Section 13.3.

15.2 Administration of Patient-Completed Questionnaires

a. Patients are to complete questionnaires at registration (following consent and prior to or on the same day as initiation of systemic therapy), at the end of the first cycle of systemic therapy and by 6 months after registration. (See Section 9.0 and 14.0 for the list of questionnaires and time points they are to be administered.) Questionnaires are anticipated to require 15-20 minutes to complete at each study time point.

b. In order to minimize patient burden and streamline patient visits, it is preferable for questionnaires to be given to the patient at the clinic visit immediately prior to the clinician assessment; however, the study staff should accommodate thepatient's preferences for filling out the questionnaires as described below. Questionnaires may be taken home and returned by mail to the component within ± 1 week of the assessment timepoint.

c. The research site should provide the patient with options for completing the questionnaires after the patient has reviewed them. The patient's review may help them decide if they need information they do not have with them at their visitor they may need assistance from a family member or caregiver.

d. As a general reminder, review all completed questionnaires to be sure all of the questions have been answered and, when the patient is directed to mark onlyone response, that only one answer is marked. If the patient has marked more than one answer per question, ask which answer reflects how the patient is feeling. If the patient has skipped a question, tell the patient that a question was not answered and ask if the patient would like to answer the question. If the patient is unable to answer the question at the time of the visit, site staffs are encouraged to retain the questionnaire and contact the patient by phone to obtain outstanding information. If patient does not want to answer a particular question, the CRA will enter "Not answered by the patient" in Medidata RAVE®.

e. Caregivers may assist patients with their questionnaires by administering the questionnaire orally to the patient, helping the patient find information and/or recording the patient's answers. Caregivers cannot answer for the patient unlessthe questionnaire specifically indicates that the caregiver may do so. For patients who are too sick to complete the questionnaires (even with assistance from the caregiver) or who are not able to come to a clinic visit (e.g. enrolled in hospice care), the CRA will record on the **S1415CD** Cover Sheet that the patient was too sick to complete the questionnaire.

f. **S1415CD** Cover Sheet for Patient-Completed Questionnaires

For each time point, the nurse or CRA completes the **S1415CD** Cover Sheet for Patient-Completed Questionnaires. The Cover Sheet is submitted with the set of patient-completed forms at each scheduled assessment. The Cover Sheet is very important for tracking how and when the patient forms were completed. When a patient-completed form is not administered at a scheduled time point, itis important to know why the assessment did not occur; the form includespotential reasons for a patient not completing a form. All issues of noncomplianceare noted on the **S1415CD** Cover Sheet.

15.3 Optional Training for Administration of Patient-Completed Questionnaires:

Anyone involved in the collection of quality of life data in SWOG trials should review thetraining program available on the SWOG website accessible from three locations. On the

SWOG Authorized Document

SWOG Home Page (prior to member login), in the QUICKLINKS section on the bottom right corner of the page, there is a link to the Patient Reported Outcomes Training. The other two locations that the training is available are after SWOG member login on the CRA Workbench. The Training section and the New CRAs! Section both contain access to the Patient Reported Outcomes (PROs) training module. The training program is a narrated set of slides designed to standardize the way quality of life data is collected frompatients. Questions regarding the quality of life assessments can be addressed to the SWOG Data Operations Office (206-652-2267).

16.0 ETHICAL AND REGULATORY CONSIDERATIONS

The following must be observed to comply with Food and Drug Administration regulations for the conduct and monitoring of clinical investigations; they also represent sound research practice:

Informed Consent

The principles of informed consent are described by Federal Regulatory Guidelines (Federal Register Vol. 46, No. 17, January 27, 1981, part 50) and the Office for Protection from Research Risks Reports: Protection of Human Subjects (Code of Federal Regulations 45 CFR 46). They must be followed to comply with FDA regulations for the conduct and monitoring of clinical investigations.

Institutional Review

This study must be approved by an appropriate institutional review committee as defined by Federal Regulatory Guidelines (Ref. Federal Register Vol. 46, No. 17, January 27, 1981, part 56) and the Office for Protection from Research Risks Reports: Protection of Human Subjects (Code of Federal Regulations 45 CFR 46).

Monitoring

This study will be monitored by the Clinical Data Update System (CDUS) Version 3.0. Cumulative, abbreviated CDUS data will be submitted quarterly to CTEP by electronic means. Reports are due January 31, April 30, July 31 and October 31.

Confidentiality

Please note that the information contained in this protocol is considered confidential and should not be used or shared beyond the purposes of completing protocol requirements until or unless additional permission is obtained.

Adverse Events

16.1 Adverse Event Reporting Requirements

a. Purpose

Adverse event data collection and reporting, which are required as part of every clinical trial, are done to ensure the safety of patients enrolled in the studies as well as those who will enroll in future studies using similar agents. Adverse events are reported in a routine manner at scheduled times during a trial. (Directions for routine reporting are provided in Section 14.0.) Additionally, certain adverse events must be reported in an expedited manner to allow formore timely monitoring of patient safety and care. The following guidelines prescribe expedited adverse event reporting for this protocol.

SWOG Authorized Document

b. Reporting method

This study requires that expedited adverse events be reported using the CancerTherapy Evaluation Program Adverse Event Reporting System (CTEP-AERS).CTEP's guidelines for CTEP-AERS can be found at http://ctep.cancer.gov. ACTEP-AERS report must be submitted to the SWOG Operations Officeelectronically via the CTEP-AERS Web-based application located at http://ctep.cancer.gov/protocolDevelopment/electronic_applications/adverse_eve nts.htm.

In the rare event when internet connectivity is disrupted an electronic reportMUST be submitted immediately upon re-establishment of internet connection.

c. When to report an event in an expedited manner

When the adverse event requires expedited reporting, submit the report within 10calendar days of learning of the event.

d. Other recipients of adverse event reports

The SWOG Operations Office will forward reports and documentation to the appropriate regulatory agencies and drug companies as required.

Adverse events determined to be reportable to the Institutional Review Board responsible for oversight of the patient must be reported according to local policy and procedures.

e. Expedited reporting for commercial agents

Commercial reporting requirements are provided in Table 16.1 and are limited to the commercial agent(s) of interest. The commercial agents for this study are Colony Stimulating Factors (CSFs). If there is any question about the reportabilityof an adverse event or if on-line CTEP-AERS cannot be used, please telephone or email the SAE Program at the Operations Office, 210/614-8808 oradr@swog.org, before preparing the report.

SWOG Authorized Document

Table 16.1. Expedited reporting requirements for adverse events experienced bypatients using CSFs within 30 days of the last administration of the CSF.

**ATTRIBUTION**	**Grade 4**		**Grade 5[a]**	
	Unexpected	Expected	Unexpected	Expected
Unrelated or Unlikely			**CTEP-AERS**	**CTEP-AERS**
Possible, Probable, Definite	**CTEP-AERS**		**CTEP-AERS**	**CTEP-AERS**

CTEP-AERS: Indicates an expedited report is to be submitted via CTEP-AERSwithin 10 calendar days of learning of the event[b].

[a] This includes all deaths within 30 days of the last dose of treatment with a commercial agent(s), regardless of attribution. Any death that occurs more than 30 days after the last dose of treatment with a commercial agent(s) and isattributed (possibly, probably, or definitely) to the agent(s) and is not due to cancer recurrence must be reported according to the instructions above.

[b] Submission of the on-line CTEP-AERS report plus any necessary amendments generally completes the reporting requirements. You may, however, be asked to submit supporting clinical data to the Operations Office in order to complete the evaluation of the event. If requested, the specified data should be sent within 5 calendar days by fax to 210-614-0006.

f. Reporting Pregnancy, Fetal Death, and Death Neonatal

1. **Pregnancy** Study participants who become pregnant while on study; that pregnancy should be reported in an expedited manner via CTEP-AERS as **Grade 3 "Pregnancy, puerperium and perinatal conditions – Other (pregnancy)"** under the **Pregnancy, puerperium and perinatal conditions** SOC.

   Additionally, the pregnancy outcome for patients on study should be reported via CTEP-AERS at the time the outcome becomes known,accompanied by the same Pregnancy Report Form used for the initial report.

2. **Fetal Death Fetal Death defined in CTCAE as "A disorder characterized by** death in utero; failure of the product of conception to show evidence of respiration, heartbeat, or definite movement of a voluntary muscle after expulsion from the uterus, without possibility of resuscitation" should be reported expeditiously as **Grade 4 "pregnancy, puerperium and perinatal conditions – Other (pregnancy loss)" under the Pregnancy, puerperium and perinatal conditions** SOC.

3. **Death Neonatal** Neonatal death, defined in CTCAE as "A disorder characterized by cessation of life occurring during the first 28 days of life"that is felt by the investigator to be at least possibly due to the investigational agent/intervention should be reported expeditiously.

   A neonatal death should be reported expeditiously as **Grade 4 "General disorders and administration – Other (neonatal loss)"** under the **General disorders and administration** SOC.

SWOG Authorized Document

*Fetal death and neonatal death should **NOT** be reported as a Grade 5 event. If reported as such, the CTEP-AERS interprets this as a death of the patient being treated.*

NOTE: When submitting CTEP-AERS reports for “Pregnancy, “Pregnancy loss”, or “Neonatal loss”, the Pregnancy Information Form should also be completed and faxed with any additional medical information to 301-230-0159. The potential risk of exposure of the fetus to the investigational agent(s) or chemotherapy agent(s) should be documented in the “Description of Event”section of the CTEP-AERS report.

The Pregnancy Information Form is available at:
http://ctep.cancer.gov/protocolDevelopment/adverse_effects/htm.

SWOG Authorized Document

17.0 BIBLIOGRAPHY

1. Siegel R, Ma J, Zou Z, Jemal A. Cancer statistics CA Cancer J Clin 2014;64:9-29, 2014.

2. Kuderer NM, Dale DC, Crawford J, Cosler LE, Lyman GH. Mortality, morbidity, and cost associated with febrile neutropenia in adult cancer patients. Cancer 106:2258-66, 2006.

3. Culakova E, Thota R, Poniewierski MS, et al. Patterns of chemotherapy-associated toxicity and supportive care in US oncology practice: a nationwide prospective cohort study. Cancer medicine3:434-44, 2014.

4. Kuderer NM, Dale DC, Crawford J, Cosler LE, Lyman GH. Mortality, morbidity, and cost associated with febrile neutropenia in adult cancer patients. Cancer 106:2258-66, 2006.

5. Aapro MS, Bohlius J, Cameron DA, et al. 2010 update of EORTC guidelines for the use of granulocyte-colony stimulating factor to reduce the incidence of chemotherapy-induced febrile neutropenia in adult patients with lymphoproliferative disorders and solid tumours. Eur J Cancer 47:8-32, 2011.

6. Potosky AL, Malin JL, Kim B, et al. Use of colony-stimulating factors with chemotherapy: opportunities for cost savings and improved outcomes. J Natl Cancer Inst 103:979-82, 2011.

7. Ramsey SD, McCune JS, Blough DK, et al. Colony-stimulating factor prescribing patterns in patients receiving chemotherapy for cancer. Am J Manag Care 16:678-86, 2010.

8. Crawford J, Dale DC, Kuderer NM, et al. Risk and timing of neutropenic events in adult cancer patients receiving chemotherapy: the results of a prospective nationwide study of oncologypractice. Journal of the National Comprehensive Cancer Network : JNCCN 6:109-18, 2008.

9. Smith TJ, Khatcheressian J, Lyman GH, et al. 2006 update of recommendations for the use of white blood cell growth factors: an evidence-based clinical practice guideline. J Clin Oncol24:3187-205, 2006.

10. Schnipper LE, Smith TJ, Raghavan D, et al. American Society of Clinical Oncology identifies five key opportunities to improve care and reduce costs: the top five list for oncology. J Clin Oncol 30:1715-24, 2012.

11. Clinical Practice Guidelines in Oncology: Breast Cancer, v.1.2010. 2010. (Accessed November 2,2011, at http://www.nccn.org/professionals/physician_gls/f_guidelines.asp.)

12. Clinical Practice Guidelines in Oncology: Myeloid Growth Factors, v.1.2010. 2010. (Accessed November 2, 2011, at http://www.nccn.org/professionals/physician_gls/f_guidelines.asp.)

13. Clinical Practice Guidelines in Oncology: Non-small Cell Lung Cancer, v.2.2009. 2009. (AccessedNovember 2, 2011, at http://www.nccn.org/professionals/physician_gls/f_guidelines.asp.)

14. Smith TJ, Khatcheressian J, Lyman GH, et al. 2006 update of recommendations for the use of white blood cell growth factors: an evidence-based clinical practice guideline. J Clin Oncol24:3187-205, 2006.

15. McCune JS, Sullivan SD, Blough DK, et al. Colony-stimulating factor use and impact on febrile neutropenia among patients with newly diagnosed breast, colorectal, or non-small cell lungcancer who were receiving chemotherapy. Pharmacotherapy 32:7-19, 2012.

SWOG Authorized Document

16. Hershman D, Hurley D, Wong M, Morrison VA, Malin JL. Impact of primary prophylaxis on febrile neutropenia within community practices in the US. J Med Econ 12:203-10, 2009.

17. *Ramsey SD, McCune JS, Blough DK, et al. Colony-stimulating factor prescribing patterns in patients receiving chemotherapy for cancer. Am J Manag Care 16:678-86, 2010.*

18. Krzemieniecki K, Sevelda P, Erdkamp F, et al. Neutropenia management and granulocyte colony-stimulating factor use in patients with solid tumours receiving myelotoxic chemotherapy--findings from clinical practice. Support Care Cancer 22:667-77, 2014.

19. Kalinka-Warzocha E, Plazas JG, Mineur L, et al. Chemotherapy treatment patterns and neutropenia management in gastric cancer. Gastric cancer : official journal of the International Gastric Cancer Association and the Japanese Gastric Cancer Association 2014.

20. Langeberg WJ, Siozon CC, Page JH, Morrow PK, Chia VM. Use of pegfilgrastim primary prophylaxis and risk of infection, by chemotherapy cycle and regimen, among patients with breastcancer or non-Hodgkin's lymphoma. Support Care Cancer 22:2167-75, 2014.

21. Choi MR, Solid CA, Chia VM, et al. Granulocyte colony-stimulating factor (G-CSF) patterns of usein cancer patients receiving myelosuppressive chemotherapy. Support Care Cancer 22:1619-28, 2014.

22. *Ramsey SD, McCune JS, Blough DK, et al. Colony-stimulating factor prescribing patterns in patients receiving chemotherapy for cancer. Am J Manag Care 16:678-86, 2010.*

23. Lyman GH, Dale DC, Wolff DA, et al. Acute myeloid leukemia or myelodysplastic syndrome in randomized controlled clinical trials of cancer chemotherapy with granulocyte colony-stimulating factor: a systematic review. J Clin Oncol 28:2914-24, 2010.

24. *Ramsey SD, McCune JS, Blough DK, et al. Colony-stimulating factor prescribing patterns in patients receiving chemotherapy for cancer. Am J Manag Care 16:678-86, 2010.*

25. Neulasta: Highlights of Prescribing Information. Amgen Manufacturing, Limited, 2014. (Accessed Aug 1, 2014, at http://pi.amgen.com/united_states/neulasta/neulasta_pi_hcp_english.pdf.)

26. Neupogen (Filgrastim) Package Insert. Amgen Manufacturing, Limited, 2013. (Accessed Aug 1, 2014, at http://pi.amgen.com/united_states/neupogen/neupogen_pi_hcp_english.pdf.)

27. Vogel CL, Wojtukiewicz MZ, Carroll RR, et al. First and subsequent cycle use of pegfilgrastim prevents febrile neutropenia in patients with breast cancer: a multicenter, double-blind, placebo- controlled phase III study. J Clin Oncol 23:1178-84, 2005.

28. Smith TJ, Khatcheressian J, Lyman GH, et al. 2006 update of recommendations for the use of white blood cell growth factors: an evidence-based clinical practice guideline. J Clin Oncol24:3187-205, 2006.

29. Clinical Practice Guidelines in Oncology: Myeloid Growth Factors, v.1.2010. 2010. (Accessed November 2, 2011, at http://www.nccn.org/professionals/physician_gls/f_guidelines.asp.)

30. Lyman GH, Kleiner JM. Summary and comparison of myeloid growth factor guidelines in patients receiving cancer chemotherapy. Cancer treatment and research 157:145-65, 2011.

31. Ramsey S, Blough D, Kirchhoff A, et al. Washington State cancer patients found to be at greater risk for bankruptcy than people without a cancer diagnosis. Health Aff (Millwood) 32:1143-52, 2013.

SWOG Authorized Document

32. Cosgrove J. Information on Highest Expenditure Part B Drugs. In. http://www.gao.gov/assets/660/655608.pdf; 2013.

33. Schnipper LE, Smith TJ, Raghavan D, et al. American Society of Clinical Oncology identifies five key opportunities to improve care and reduce costs: the top five list for oncology. J Clin Oncol 30:1715-24, 2012.

34. 10 Things Physicians and Patients Should Question. American Society of Clinical Oncology, 2013. (Accessed Aug 6, 2014, at http://www.choosingwisely.org/doctor-patient-lists/american-society-of-clinical-oncology/.)

35. Bennett CL, Smith TJ, Weeks JC, et al. Use of hematopoietic colony-stimulating factors: the American Society of Clinical Oncology survey. The Health Services Research Committee of ASCO. J Clin Oncol 1996;14:2511-20.

36. Bennett CL, Weeks JA, Somerfield MR, Feinglass J, Smith TJ. Use of hematopoietic colony- stimulating factors: comparison of the 1994 and 1997 American Society of Clinical Oncology surveys regarding ASCO clinical practice guidelines. Health Services Research Committee of ASCO. J Clin Oncol 17:3676-81, 1999.

37. Fishman ML, Kumar A, Davis S, Shimp W, Hrushesky WJ. Guideline-Based Peer-to-Peer Consultation Optimizes Pegfilgrastim Use With No Adverse Clinical Consequences. J Oncol Pract8:e14s-7s, 2012.

38. Krzemieniecki K, Sevelda P, Erdkamp F, et al. Neutropenia management and granulocyte colony-stimulating factor use in patients with solid tumours receiving myelotoxic chemotherapy--findings from clinical practice. Support Care Cancer 22:667-77, 2014.

39. Choi MR, Solid CA, Chia VM, et al. Granulocyte colony-stimulating factor (G-CSF) patterns of usein cancer patients receiving myelosuppressive chemotherapy. Support Care Cancer 22:1619-28, 2014.

40. Cancer Treatment & Survivorship Facts & Figures. American Cancer Society, 2014-2015.(Accessed Aug 6, 2014, at http://www.cancer.org/acs/groups/content/@research/documents/document/acspc-042801.pdf.)

41. Calhoun EA, Chang CH, Welshman EE, Cella D, Grp AS. Development and validation of theFACT-Neutropenia. Blood 98:427a-a, 2001.

42. Ropka ME, Padilla G. Assessment of neutropenia-related quality of life in a clinical setting. OncolNurs Forum 34:403-9, 2007.

43. Functional Assessment Cancer Therapy- Neutropenia FACT-N. FACIT, 2007. (Accessed Aug 5,2014, at http://www.facit.org/facitorg/questionnaires.)

44. Aapro MS, Bohlius J, Cameron DA, et al. 2010 update of EORTC guidelines for the use of granulocyte-colony stimulating factor to reduce the incidence of chemotherapy-induced febrile neutropenia in adult patients with lymphoproliferative disorders and solid tumours. Eur J Cancer 47:8-32, 2011.

45. Potosky AL, Malin JL, Kim B, et al. Use of colony-stimulating factors with chemotherapy:opportunities for cost savings and improved outcomes. J Natl Cancer Inst 103:979-82, 2011.

46. McCune J, Sullivan S, Clarke L, et al. Colony Stimulating Factor Use and Impact on Febrile Neutropenia among Patients with Newly Diagnosed Breast, Colorectal, or Non-small Cell Lung Cancer Receiving Chemotherapy. Pharmacotherapy. Pharmacotherapy In Press.

SWOG Authorized Document

47. Morrison VA, Wong M, Hershman D, Campos LT, Ding B, Malin J. Observational study of the prevalence of febrile neutropenia in patients who received filgrastim or pegfilgrastim associated with 3-4 week chemotherapy regimens in community oncology practices. J Manag Care Pharm 13:337-48, 2007.

48. Kuderer NM, Lyman GH. Personalized medicine and cancer supportive care: appropriate use of colony-stimulating factor support of chemotherapy. J Natl Cancer Inst 103:910-3, 2011.

49. Kuderer NM, Dale DC, Crawford J, Lyman GH. Impact of primary prophylaxis with granulocyte colony-stimulating factor on febrile neutropenia and mortality in adult cancer patients receiving chemotherapy: a systematic review. J Clin Oncol 25:3158-67, 2007.

50. Zirpoli GR, Brennan PM, Hong CC, et al. Supplement use during an intergroup clinical trial for breast cancer (S0221). Breast Cancer Res Treat 137:903-13, 2013.

51. Potosky AL, Malin JL, Kim B, et al. Use of colony-stimulating factors with chemotherapy: opportunities for cost savings and improved outcomes. J Natl Cancer Inst 103:979-82, 2011.

52. *Ramsey SD, McCune JS, Blough DK, et al. Colony-stimulating factor prescribing patterns in patients receiving chemotherapy for cancer. Am J Manag Care 16:678-86, 2010.*

53. *Ramsey SD, McCune JS, Blough DK, et al. Colony-stimulating factor prescribing patterns in patients receiving chemotherapy for cancer. Am J Manag Care 16:678-86, 2010.*

54. Aapro MS, Bohlius J, Cameron DA, et al. 2010 update of EORTC guidelines for the use of granulocyte-colony stimulating factor to reduce the incidence of chemotherapy-induced febrile neutropenia in adult patients with lymphoproliferative disorders and solid tumours. Eur J Cancer47:8-32, 2011.

55. Smith TJ, Khatcheressian J, Lyman GH, et al. 2006 update of recommendations for the use ofwhite blood cell growth factors: an evidence-based clinical practice guideline. J Clin Oncol 24:3187-205, 2006.

56. Clinical Practice Guidelines in Oncology: Myeloid Growth Factors, v.1.2010. 2010. (Accessed November 2, 2011, at http://www.nccn.org/professionals/physician_gls/f_guidelines.asp.)

57. Clinical Practice Guidelines in Oncology: Non-small Cell Lung Cancer, v.2.2009. 2009. (Accessed November 2, 2011, at http://www.nccn.org/professionals/physician_gls/f_guidelines.asp.)

58. Smith K, Wray L, Klein-Cabral M, et al. Ethnic disparities in adjuvant chemotherapy for breast cancer are not caused by excess toxicity in black patients. Clin Breast Cancer6:260-6; discussion7-9, 2005.

59. Lyman GH, Kuderer NM, Crawford J, et al. Predicting individual risk of neutropenic complicationsin patients receiving cancer chemotherapy. Cancer 117:1917-27, 2011.

SWOG Authorized Document

18.0 APPENDIX

18.1 **S1415CD** List of Protocol Approved Regimens

SWOG Authorized Document

18.1 **S1415CD** List of Protocol Approved Regimens

a. **Concurrent Radiation is not allowed**

Regimens with concurrent radiation will not be addressed by TrACER as radiation is a contraindication for primary prophylactic growth factors. Patients undergoing concurrent radiation and order templates that include concurrent radiation (e.g. carboplatin/PACLitaxel + XRT) should NOT be considered for this study.

b. **Sequential Regimens**

Sequential regimens (e.g. AC-> T) will not be addressed jointly by TrACER. Eligibility should be determined based solely on the first regimen administered to a patient regardless of subsequent regimens (e.g. by AC only in the sequence AC-> T).

c. **Determining Chemotherapy Order Eligibility**

When determining which regimens to target in the existing EMR/paper order system, the practice should include regimens that are identified in its system as targeting breast, non-small cell lung, or colorectal cancer only, or in the absence of a disease-specific counterpart, either any superset of these diseases (e.g. lungis a superset of non-small cell lung) or no specified disease site. See Table 1 below.

Table 1: EXAMPLES for Determining Chemotherapy Order Eligibility

STUDY REGIMEN (from Table 3)	CHEMOTHERAPY ORDER IN SYSTEM	CHEMOTHERAPYORDER ELIGIBLE FOR STUDY	RATIONALE
NSCLC CARBOplatin/etoposide	NSCLC carboplatin/etoposide	Yes	NSCLC is the specific disease targeted by this study
NSCLC CARBOplatin/etoposide	SCLC carboplatin/etoposide	No	NSCLC is not atype of SCLC
NSCLC CARBOplatin/etoposide	LUNG carboplatin/etoposide<br>*(NSCLC carboplatin/etoposide NOT in system)*	Yes	NSCLC is a type of lung cancer and there is no other regimen with these same agents specifically targeting NSCLC
NSCLC CARBOplatin/etoposide	Carboplatin/etoposide<br>*(NSCLC carboplatin/etoposide NOT in system)*	Yes	No specific disease targeted and there is no other regimen withthese same agents specifically targeting NSCLC
NSCLC CARBOplatin/etoposide	LUNG carboplatin/etoposide<br>*(NSCLC carboplatin/etoposide in system)*	No	There is another regimen in the system with these same agents specifically targeting NSCLC.In this

			case, only

SWOG Authorized Document

Table 1: EXAMPLES for Determining Chemotherapy Order Eligibility			
**STUDY REGIMEN (from Table 3)**	**CHEMOTHERAPY ORDER IN SYSTEM**	**CHEMOTHERAPYORDER ELIGIBLE FOR STUDY**	**RATIONALE**
			NSCLC regimen that matches the regimen listed in Table 3 is eligible for the study.
BREAST Gemcitabine	BREAST gemcitabine	Yes	Breast cancer is the specific disease targeted by this study
BREAST Gemcitabine	OVA gemcitabine	No	Breast cancer is not a type of ovarian cancer

d. **Multiple Chemotherapy Orders**

Determining appropriate CSF standing order defaults across multiple chemotherapy orders is beyond the scope of this study. For this reason, patients on multiple chemotherapy orders to be administered within the very first cycle of chemotherapy (including monoclonal antibodies) are ineligible for participation. See Table 2 below.

Table 2: EXAMPLES for Multiple Chemotherapy Orders			
**STUDY REGIMEN (from Table 3)**	**CHEMOTHERAPY ORDER(S) IN SYSTEM**	**CHEMOTHERAPY ORDER ELIGIBLE FOR STUDY**	**RATIONALE**
BREAST TC	Order 1: BREAST TC starting 1/1/2016	Yes	All agents in theregimen are included in oneorder
BREAST TC	Order 1: docetaxel starting on 1/1/2016<br><br>Order 2: cyclophosphamide starting on 1/1/2016	No	The agents that make up the regimen are ordered separately; the study does not support multiple regimen orders.
BREAST PACLitaxel weekly + trastuzumab	Order 1: BREAST paclitaxel weekly + Trastuzumab, cycle 1 starting 1/1/2016	Yes	All agents that make up the regimen are in the same order
BREAST PACLitaxel weekly + trastuzumab	Order 1: BREAST paclitaxel weekly, cycle 1 starting 1/1/2016<br><br>Order 2: Trastuzumab, cycle 1 starting	No	The agents that make up the regimen are ordered separately; the study does not support multiple regimen orders.

SWOG Authorized Document

	1/1/2016		

e. **For Intervention Arm Components**

Components should only change those regimens that already exist and are used in their ordering system. No practice is expected to add/remove regimens to/fromtheir system as part of this study.

Regimens should only be modified if they *exactly match* the regimen listed in Table 3 below. Regimens should match in dose, cycle length, and chemotherapy drug(s), including any monoclonal antibodies. All regimens derived from NCCN chemotherapy templates at www.nccn.org. Contact TrACER@fredhutch.org withquestions.

Effective December 2, 2019, only registration of patients on regimens with intermediate FN risk will be allowed for randomized sites (Groups 2, 3 and 4). Offlimit regimens are shaded in gray.

TABLE 3

Non- Small Cell Lung Cancer		
**Regimen**	**Cycle Length**	**FN Risk**
Afatinib	1 day	Low
Albumin-bound PACLitaxel	21 days	Low
Bevacizumab	21 days	Low
CARBOplatin/albumin-bound PACLitaxel	21 days	Low
CARBOplatin/albumin-bound PACLitaxel + pembrolizumab	21 days	Low
CARBOplatin/PACLitaxel + pembrolizumab	21 days	Intermediate
CARBOplatin/DOCEtaxel	21 days	Intermediate
CARBOplatin/etoposide	21 days	Intermediate
CARBOplatin/gemcitabine	28 days	Intermediate
CARBOplatin/PACLitaxel + atezolizumab + bevacizumab	21 days	Intermediate
CARBOplatin/PACLitaxel + bevacizumab	21 days	Intermediate
CARBOplatin/PACLitaxel	21 days	Intermediate
CARBOplatin/PEMEtrexed + bevacizumab	21 days	Low
CARBOplatin/PEMEtrexed	21 days	Low
CARBOplatin/PEMEtrexed + pembrolizumab	21 days	Low
CARBOplatin/vinORELBine	21 days	Low
Ceritinib	1 day	Low
CISplatin/albumin-bound PACLitaxel + pembrolizumab	21 days	Low
CISplatin/DOCEtaxel	21 days	Intermediate
CISplatin/etoposide	21-28 days	Intermediate
CISplatin/gemcitabine	21 days	Low
CISplatin/gemcitabine + necitumumab	21 days	Low
CISplatin/PACLitaxel	21 days	Intermediate
CISplatin/PACLitaxel + pembrolizumab	21 days	Intermediate
CISplatin/PEMEtrexed + bevacizumab	21 days	Low
CISplatin/PEMEtrexed	21 days	Low

SWOG Authorized Document

Non- Small Cell Lung Cancer		
**Regimen**	**Cycle Length**	**FN Risk**
CISplatin/PEMEtrexed + pembrolizumab	21 days	Low
CISplatin/vinORELBine every 21 days	21 days	Intermediate
CISplatin/vinORELBine every 28 days	28 days	Intermediate
CISplatin/vinORELBine + cetuximab	21 days	Intermediate
Crizotinib	1 day	Low
DOCEtaxel	21 days	Intermediate
DOCEtaxel + ramucirumab	21 days	Intermediate
Erlotinib	1 day	Low
Etoposide (oral)	28-35 days	Intermediate
Gemcitabine	21-28 days	Low
Gemcitabine/DOCEtaxel	21 days	Intermediate
Irinotecan	21 days	Low
PACLitaxel	21-28 days	Low
PEMEtrexed	21 days	Low
Pembrolizumab	21 days	Low
PEMEtrexed + bevacizumab	21 days	Low
VinORELBine	21 days	Low
VinORELBine/gemcitabine	21 days	Low

Colorectal Cancer		
**Regimen**	**Cycle Length**	**FN Risk**
Capecitabine	21 days	Low
Capecitabine + bevacizumab	21 days	Low
CapeOX (XELOX) (capecitabine, oxaliplatin)	21 days	Low
CapeOX (XELOX) + bevacizumab (capecitabine, oxaliplatin)	21 days	Low
Cetuximab	7 days	Low
FLOX (fluorouracil, leucovorin, oxaliplatin)	56 days	Low
Fluorouracil/leucovorin weekly	7 days	Low
Fluorouracil/leucovorin weekly + bevacizumab	7 days	Low
FOLFIRI (leucovorin, fluorouracil, irinotecan)	14 days	Low
FOLFIRI + afilbercept (leucovorin, fluorouracil, irinotecan)	14 days	Low
FOLFIRI + bevacizumab (leucovorin, fluorouracil, irinotecan)	14 days	Low
FOLFIRI + cetuximab (leucovorin, fluorouracil, irinotecan)	14 days	Low
FOLFIRI + panitumumab (leucovorin, fluorouracil, irinotecan)	14 days	Low
FOLFIRI + ramucirumab (leucovorin, fluorouracil, irinotecan)	14 days	Low
FOLFOX4 (leucovorin, fluorouracil, oxaliplatin)	14 days	Intermediate
FOLFOXIRI (leucovorin, fluorouracil, oxaliplatin, irinotecan)	14 days	Low

SWOG Authorized Document

Colorectal Cancer		
**Regimen**	**Cycle Length**	**FN Risk**
FOLFOXFIRI + bevacizumab (leucovorin, fluorouracil, oxaliplatin, irinotecan)	14 days	Low
Irinotecan	21 days	Low
Irinotecan every 14 days	14 days	Low
Irinotecan + bevacizumab	14 days	Low
Irinotecan every 14 days + aflibercept	14 days	Low
Irinotecan every 14 days + cetuximab	14 days	Low
Irinotecan every 14 days + panitumumab	14 days	Low
Irinotecan every 14 days + ramucirumab	14 days	Low
Irinotecan every 21 days + cetuximab	21 days	Low
Irinotecan + cetuximab + vemurafenib	14 days	Low
Irinotecan + panitumumab + vemurafenib	14 days	Low
IROX (irinotecan, oxaliplatin)	21 days	Low
IROX (irinotecan, oxaliplatin) + bevacizumab	21 days	Low
mFOLFOX6 (leucovorin, fluorouracil, oxaliplatin)	14 days	Intermediate
mFOLFOX6 + bevacizumab (leucovorin, fluorouracil, oxaliplatin)	14 days	Intermediate
mFOLFOX6 + cetuximab (leucovorin, fluorouracil, oxaliplatin)	14 days	Intermediate
mFOLFOX6 + panitumumab (leucovorin, fluorouracil, oxaliplatin)	14 days	Intermediate
mFOLFOX7 (leucovorin, fluorouracil, oxaliplatin)	14 days	Intermediate
Panitumumab	14 days	Low
Roswell Park fluorouracil/leucovorin	56 days	Low
Roswell Park fluorouracil/leucovorin + bevacizumab	56 days	Low
Simplified biweekly infusional fluorouracil/leucovorin	14 days	Low
Simplified biweekly infusional fluorouracil/leucovorin + bevacizumab	14 days	Low

Breast Cancer		
**Regimen**	**Cycle Length**	**FN Risk**
AC every 21 days (DOXOrubicin, cyclophosphamide)	21 days	Intermediate
Ado-trastuzumab emantasine	21 days	Low
Albumin-bound PACLitaxel	21 days	Low
Albumin-bound PACLitaxel every 21 days + trastuzumab	21 days	Low
Albumin-bound PACLitaxel every 28 days + trastuzumab	28 days	Low
Anastrozole + trastuzumab	21 days	Low
CAF (cyclophosphamide [oral], DOXOrubicin, fluorouracil)	28 days	Low
Capecitabine	21 days	Low

SWOG Authorized Document

Breast Cancer		
**Regimen**	**Cycle Length**	**FN Risk**
Capecitabine + trastuzumab	21 days	Low
CARBOplatin	21-28 days	Low
CARBOplatin + trastuzumab	21-28 days	Low
CARBOplatin/gemcitabine	21 days	Intermediate
CARBOplatin/PACLitaxel every 21 days + trastuzumab	21 days	Intermediate
CARBOplatin/PACLitaxel weekly + trastuzumab	28 days	Low
CEF (cyclophosphamide [oral], epirubicin, fluorouracil)	28 days	Intermediate
CISplatin	21 days	Low
CISplatin + trastuzumab	21 days	Low
CMF Bonadonna (classic) (oral cyclophosphamide, I.V.methotrexate, fluorouracil)	28 days	Intermediate
CMF (IV cyclophosphamide, methotrexate, fluorouracil)	21 days	Intermediate
Cyclophosphamide	28 days	Low
Cyclophosphamide + trastuzumab	28 days	Low
DOCEtaxel every 21 days	21 days	Intermediate
DOCEtaxel every 21 days + pertuzumab + trastuzumab	21 days	Intermediate
DOCEtaxel every 21 days + trastuzumab	21 days	Intermediate
DOCEtaxel weekly	7 days	Low
DOCEtaxel weekly + trastuzumab	7 days	Low
DOCEtaxel/capecitabine	21 days	Intermediate
Dose-dense AC (DOXOrubicin, cyclophosphamide)	14 days	High
Dose-dense PACLitaxel	14 days	High
Dose-dense PACLitaxel + trastuzumab	14 days	High
DOXOrubicin every 21 days	21 days	Intermediate
DOXOrubicin weekly	7 days	Low
EC every 21 days (EPIrubicin, cyclophosphamide)	21 days	Low
EPIrubicin	21 days	Low
EriBULin	21 days	Low
EriBULin + trastuzumab	21 days	Low
Everolimus + exemestane	1 day	Low
FAC (fluorouracil, DOXOrubicin, cyclophosphamide)	21 days	Low
FEC (fluorouracil, epirubicin, cyclophosphamide)	21 days	Intermediate
Gemcitabine	28 days	Low
Gemcitabine + trastuzumab	28 days	Low
Ixabepilone	21 days	Low
Ixabepilone + trastuzumab	21 days	Low
Lapatinib + letrozole	1 day	Low

SWOG Authorized Document

Breast Cancer		
**Regimen**	**Cycle Length**	**FN Risk**
Lapatinib + trastuzumab	7 days	Low
Lapatinib/capecitabine	21 days	Low
Liposomal DOXOrubicin	28 days	Low
PACLitaxel + bevacizumab	28 days	Low
PACLitaxel every 21 days	21 days	Intermediate
PACLitaxel every 21 days + trastuzumab	21 days	Intermediate
PACLitaxel every 21 days + trastuzumab + pertuzumab	21 days	Intermediate
PACLitaxel weekly	7 days	Low
PACLitaxel weekly + trastuzumab	7 days	Low
PACLitaxel weekly + trastuzumab + pertuzumab	7 days	Low
PACLitaxel/gemcitabine	21 days	Low
Palbociclib + fulvestrant	28 days	Low
Palbociclib + letrozole	28 days	Low
Pertuzumab + trastuzumab	21 days	Low
TAC (DOCEtaxel, DOXOrubicin, cyclophosphamide)	21 days	High
TC (DOCEtaxel, cyclophosphamide)	21 days	High
TC + trastuzumab (DOCEtaxel, cyclophosphamide)	21 days	High
TCH (DOCEtaxel, CARBOplatin, trastuzumab)	21 days	High
TCH + pertuzumab (DOCEtaxel, CARBOplatin, trastuzumab)	21 days	High
Trastuzumab	21 days	Low
VinORELBine	7 days	Low
VinORELBine every 21 days + trastuzumab	21 days	Intermediate
VinORELBine + trastuzumab	7 days	Low

SWOG Authorized Document

Informed Consent Model for S1415CD

*NOTES FOR LOCAL INSTITUTION INFORMED CONSENT AUTHORS:

This model informed consent form has been reviewed by the DCTD/NCI and is the official consent document for this study. Local IRB changes to this document are allowed. (Institutionsshould attempt to use sections of this document that are in bold type in their entirety.) Editorialchanges to these sections may be made as long as they do not change information or intent. If the institutional IRB insists on making additions, deletions or more substantive modifications tothe risks or alternatives sections, they may be justified in writing by the investigator and approved by the IRB. Under these circumstances, the revised language, justification and a copyof the IRB minutes must be forwarded to the SWOG Operations Office for approval before a patient may be registered to this study.

Please particularly note that the questions related to banking of specimens for future studyare in bolded type and may not be changed in any way without prior approval from the SWOG Operations Office.

Readability Statistics:
Flesch Reading Ease _61.3_ (targeted above 55)
Flesch-Kincaid Grade Level _8.8 (targeted below 8.5)

- Instructions and examples for informed consent authors are in *[italics]*.
- A blank line,_______________, indicates that the local investigator should provide the appropriate information before the document is reviewed with the prospective research participant.
- The term "study doctor" has been used throughout the model because the local investigator for a cancer treatment trial is a physician. If this model is used for atrial in which the local investigator is not a physician, another appropriate term should be used instead of "study doctor".
- The dates of protocol updates in the header and in the text of the consent is for reference to this model only and should not be included in the informed consentform given to the prospective research participant.
- The local informed consent must state which parties may inspect the research records. This includes the NCI, the drug manufacturer for investigational studies,any companies or grantors that are providing study support (these will be listed inthe protocol's model informed consent form) and SWOG.

  "SWOG" must be listed as one of the parties that may inspect the research records in all protocol consent forms for which patient registration is being credited to SWOG. This includes consent forms for studies where all patients are registered directly through the SWOG Data Operations Office, all intergroup studies for which the registration is being credited to SWOG (whether the registration is through the SWOG Data Operations Office or directly through the other group), as

SWOG Authorized Document

well as consent forms for studies where patients are registered via CTSU and theregistration is credited to SWOG.

- When changes to the protocol require revision of the informed consent document, the IRB should have a system that identifies the revised consent document, in orderto preclude continued use of the older version and to identify file copies. An appropriate method to identify the current version of the consent is for the IRB to stamp the final copy of the consent document with the approval date. The stamped consent document is then photocopied for use. Other systems of identifying the current version of the consent such as adding a version or approval date are allowedas long as it is possible to determine during an audit that the patient signed the mostcurrent version of the consent form.

*NOTES FOR LOCAL INVESTIGATORS:

- The goal of the informed consent process is to provide people with sufficient informationfor making informed choices about participating in research. The consent form provides a summary of the study, of the individual's rights as a study participant, and documents their willingness to participate. The consent form is, however, only one piece of an ongoing exchange of information between the investigator and study participant. For more information about informed consent, review the "Recommendations for the Development of Informed Consent Documents for Cancer Clinical Trials" prepared by the Comprehensive Working Group on Informed Consent in Cancer Clinical Trials for the National Cancer Institute. The Web site address for this document is http://cancer.gov/clinicaltrials/understanding/simplification-of-informed-consent-docs/

- A blank line, "_______________", indicates that the local investigator should provide theappropriate information before submitting to the IRB.

*These notes for investigators are instructional and should not be included in the consent formsent to IRBs.

SWOG Authorized Document

Study Title for Study Participants:

"TrACER": Trial Assessing CSF prescribing Effectiveness and Risk

Official Study Title for Internet Search on http://www.ClinicalTrials.gov:

S1415CD, "A Pragmatic Trial to Evaluate a Guideline-Based Colony Stimulating Factor Standing Order Intervention and to Determine the Effectiveness of Colony Stimulating Factor Use as Prophylaxis for Patients Receiving Chemotherapy with Intermediate Risk for Febrile Neutropenia –TrialAssessing CSF Prescribing Effectiveness and Risk ("TrACER")"

Why is this study being done?

Some drugs used to treat cancer raise a patient's risk of febrile neutropenia. Febrile neutropeniais a condition that involves a fever and a low number of neutrophils (a type of white blood cell) in the blood. Having a low number of neutrophils puts a patient at risk of infection. Colony- stimulating factors (CSFs) are medications sometimes given to patients getting cancer treatmentto prevent or treat febrile neutropenia. CSFs are given as an injection under the skin or into a vein.

Current guidelines say that doctors should give CSF during cancer treatment based on how likelyit is that the drugs will raise the risk of febrile neutropenia. Research shows that many doctors do not follow these guidelines. This may be harming patients. Underuse of CSFs can raise a patient's risk for febrile neutropenia. Overuse or unneeded use of CSFs can lead to side effects, like bone and muscle pain, but give no benefit and can be costly to the patient.

In some clinics there is an automated system that helps doctors decide when to use CSFs. The system prescribes CSFs when there is a high risk that the drugs will cause febrile neutropenia. Itdoes not prescribe CSFs when there is a low risk that the drugs will cause febrile neutropenia.
The research study team wants to find out if this type of system can help doctors use CSF whenit is needed and not use it when it is not needed. The study team also wants to learn about the benefits and risks of using CSF with cancer treatment drugs that have a moderate (not high andnot low) risk of febrile neutropenia.

You are being asked to take part in this study because you will be receiving cancer treatment at aclinic that has chosen to take part in this study. Clinics that choose to take part in this study are assigned to one of the following four groups.

> Clinic Group #1: Clinics that already have an automated system that helps doctors decidewhen to use CSFs will be assigned to Group #1. These systems prescribe CSFs when there is a high risk that the cancer treatment drugs will cause febrile neutropenia. They do not prescribe CSFs when there is a low risk that the drugs will cause febrile

SWOG Authorized Document

neutropenia. The study will not ask these clinics to change what they usually do. Theseclinics will use their existing system to decide when to use CSFs. For patients at these clinics, care will not be different from care that they would receive outside the study.
There are eight clinics participating in the study in Group #1. (Effective December 2,2019, Group #1 is permanently closed to enrollment).

Clinics that do not have an automated system will be assigned to Clinic Group #2, #3, or #4. Acomputer will assign each clinic to a group by chance. This is called randomization.
Assignment is done by chance because no one knows if one study group is better or worse thanthe others.

<u>Clinic Group #2</u>: At clinics assigned to Group #2, no automated system is installed. Thestudy will not ask these clinics to change what they usually do. For patients at these clinics, care will not be different from care that they would receive outside the study.
There are eight clinics participating in the study in Group #2.

<u>Clinic Group #3</u>: Clinics assigned to Group #3 do not have an automated system, but will have an automated system installed as part of this study. The system will suggest thatCSFs be used for cancer treatment drugs that have a high risk or moderate risk of febrile neutropenia. The difference between Group #3 and Group #4 is that for clinics in Group #3, the system will suggest that CSF be used for regimens that have a moderate risk of febrile neutropenia. The system will suggest that CSFs not be used for drugs that have a low risk of febrile neutropenia. If the doctor does not agree with the suggestion from the system, he or she does not need to follow it. For patients at these clinics, cancer treatment will not be different from the treatment that they would receive outside the study. However, the study may affect whether the patient receives CSFs. There are twelve clinics participating in the study in Group #3.

<u>Clinic Group #4</u>: Clinics assigned to Group #4 do not have automated system, but will have an automated system installed as part of this study. The system will suggest that CSFs be used for cancer treatment drugs that have a high risk of febrile neutropenia. The system will suggest that CSFs not be used for drugs that have a low or moderate risk of febrile neutropenia. The difference between Group #3 and Group #4 is that for clinics in Group #4, the system will suggest that CSF *not* be used for drugs that have a moderate risk of febrile neutropenia. If the doctor does not agree with the suggestion from the system, he or she does not need to follow it. For patients at these clinics, cancer treatmentwill not be different from the treatment they would have received outside the study.
However, the study may affect whether the patient receives CSFs. There are twelveclinics participating in the study in Group #4.

Your clinic has been assigned to Group #___________.

There will be about 3,600 people taking part in this study.

SWOG Authorized Document

What is the usual approach to use of medical information for research?

If you agree to be in the study, the study team will review your medical records and yourresponses to questionnaires to answer research questions.

Hospitals or doctor's offices usually use a "Release of Medical Information" form to get medicalinformation from patients. For this study, the researchers are using a consent form that describeswhat type of information they want from your medical records. The consent form asks your permission to use this information from your medical records along with your answers to the questionnaires for research.

What are my other choices if I do not take part in this study?

Your decision to participate (or not to participate) in this research study will NOT affect your cancer treatment. If you decide not to take part in this research study, you have other choices. Forexample:

- You can get treatment for your cancer without being on a study
- You may choose to take part in a different study, if one is available

How long will I be in this study?You will be

in the study for 12 months. **What is involved?**

If you agree to take part in the study, information from your medical records will be obtained at the following times:

- At the time you join the study (baseline)
- After the first cycle of treatment
- 1 month after you join the study
- 6 months after you join the study
- 12 months after you join the study

The researchers will obtain the following information from your medical record:

- Basic information about you (e.g., gender, height, weight)
- Type of cancer
- Your medical history, current medical issues, health status
- Name of cancer treatment drugs and amount (planned and received)
- Name of CSFs and amount received
- Names of other drugs received during treatment

SWOG Authorized Document

- Occurrence of febrile neutropenia (including related hospital and emergency room visits)
- Side effects
- Blood test results

You will also be asked to answer questionnaires at the following times:

- At the time you join the study (baseline)
- After the first cycle of treatment
- 6 months after you join the study

The researchers will obtain the following information, directly from you, through the questionnaires:

- Information about you (education level, income level)
- Physical problems not related to cancer
- Information about out of pocket expenses for your treatment
- What you know about CSF drugs and febrile neutropenia
- Information about how and where you received CSFs and antibiotics
- Information about your physical, emotional, and social well-being and how well you arefunctioning

You will be given up to three questionnaires at each time point: three at baseline, two at the end of your first cycle of treatment and one at 6 months. It may take between 15-20 minutes to answerthe questionnaires (each time you answer them). The researchers would like for you to fill out thequestionnaires during the clinic visit. You may fill out the questionnaires at home, or over the phone. If you fill out the questionnaires at home, you can either mail them back to the research staff using a pre-addressed stamped envelope that will be provided, or you can bring them with you the next time you come into clinic for an appointment. The research staff will work with youto find the easiest option for you.

What extra tests and procedures will I have if I take part in this study?

No extra testing or procedures are needed for participation in this study.

What are the possible risks of taking part in this study?

If you choose to take part in this study, there is a risk that you may feel uncomfortable being askedabout your functional, physical, emotional, and social well-being.

The study team has policies in place to protect your personal information and they will do their best to make sure that the personal information used for this study will be kept private. However,they cannot guarantee total privacy. Your personal information may be given out if required by law. If information from this study is published or presented at scientific meetings, your name andother personal information will **not** be used.

SWOG Authorized Document

To help make sure your information is private, your doctor or nurse will go to a secure data submission portal sponsored by the National Cancer Institute (NCI) to send the researchers yourinformation. The research study team can then go to the same secure portal and get your information to include it with the information from all of the other patients taking part in the study.

What are the possible benefits of taking part in this study?

The information you provide will help the researchers to better understand how doctors and clinics are prescribing CSFs and if the way they are being prescribed is effective in reducingfebrile neutropenia.

Can I stop taking part in this study?

Yes. You can decide to stop at any time. If you decide to stop for any reason, it is important to letthe study doctor know as soon as possible. If you stop, you can decide whether or not to let the study doctor continue to provide your medical information to the organization running the study.

What are my rights in this study?

Taking part in this study is your choice. No matter what decision you make, and even if yourdecision changes, there will be no penalty to you. You will not lose medical care or any legalrights.

For questions about your rights while in this study, call the____*(insert name of center)* Institutional Review Board at______________________________*(insert telephone number).(Note to Local Investigator: Contact information for patient representatives or other individualsat a local institution who are not on the IRB or research team but take calls regarding clinical trial questions can also be listed here.)*

What are the costs of taking part in this study?

There are no costs for participating in the study. The research study team will arrange their research with your other medical appointments so that you do not need to make separate trips tothe clinic for this study. The research staff will provide a stamped envelope if you choose to return questionnaires by mail.

You and/or your health plan/insurance company will need to pay for all of the other costs of treating your cancer while in this study, including the cost of tests, procedures, or medicines tomanage any side effects, unless you are told that certain tests are supplied at no charge. Beforeyou decide to be in the study, you should check with your health plan or insurance company tofind out exactly what they will pay for.

SWOG Authorized Document

What happens if I am injured or hurt because I took part in this study?

There will be little risk of being injured on this study since the study team is only collectinginformation from medical records and questionnaires.

Who will see my medical information?

Your privacy is very important to the researchers and they will make every effort to protect it. The study doctors have a privacy permit to help protect your records if there is a court case. Yourinformation may be given out if required by law. For example, certain states require doctors to report to health boards if they find a disease like tuberculosis. However, the researchers will do their best to make sure that any information that is released will not identify you. Some of your health information from this study will be kept in a central database for research. Your name or contact information will not be put in the database.

There are organizations that may inspect your records. These organizations are required to makesure your information is kept private, unless required by law to provide information. Some of these organizations are:

- The study sponsor, SWOG.
- The Institutional Review Board, IRB, is a group of people who review the research withthe goal of protecting the people who take part in the study.
- National Cancer Institute
- Hutchinson Institute for Cancer Outcomes Research

[Note to Local Investigators: The NCI has recommended that HIPAA regulations be addressedby the local institution. The regulations may or may not be included in the informed consent form depending on local institutional policy.]

Where can I get more information?

You may visit the NCI Web site at http://cancer.gov/ for more information about studies or general information about cancer. You may also call the NCI Cancer Information Service to getthe same information at: 1-800-4-CANCER (1-800-422-6237).

A description of this clinical trial will be available on http://www.ClinicalTrials.gov, as required by U.S. Law. This Web site will not include information that can identify you. Atmost, the Web site will include a summary of the results. You can search this Web site at any time.

Who can answer my questions about this study?

You can talk to the study doctor about any questions or concerns you have about this study or toreport side effects or injuries. Contact the study doctor____________*(insert name of study doctor[s])* at_______*(insert telephone number).*

SWOG Authorized Document

Future Contact

Sometimes researchers working with SWOG may have another research idea that relates to people who were on a SWOG study. In some cases, to carry out the new research, researcherswould need to contact participants in a particular study. You can agree or not agree to future contact by circling "yes or no"

I agree to allow my study doctor, or someone approved by my study doctor, tocontact me regarding future research involving my participation in this study.

YES **NO**

My Signature Agreeing to Take Part in the Study

I have read this consent form or had it read to me. I have discussed it with the study doctor and my questions have been answered. I will be given a signed copy of this form. I agree to take partin this study.

Participant's (or legally authorized representative's) signature____

Date of signature____

Signature of researcher obtaining consent ____

Date of signature ____

SWOG Authorized Document

<u>Título del Estudio para Participantes:</u>

"TrACER": Ensayo para Evaluar la Eficacia y los Riesgos de recetar FactoresEstimulantes de Colonias

<u>Título Oficial del Estudio para Búsquedas conducidas por el Internet http://www.ClinicalTrials.gov:</u>

<u>S1415CD</u>, "Un ensayo pragmático para evaluar la orden permanente de intervenir con Factores Estimulantes de Colonias y el uso de ellos como profilaxis para pacientes de riesgo intermedio de neutropenia febril que recibenquimioterapia - Ensayo para Evaluar la Eficacia y los Riesgos de recetar Factores Estimulantes de Colonias"

¿Por qué se está realizando este estudio?

Unos fármacos que se utilizan en el tratamiento de cáncer aumentan el riesgo al paciente de neutropenia febril. Neutropenia febril es una condición que involucra una fiebre y cuenta baja deneutrófilos (un tipo de célula blanca) en la sangre. Al tener una cantidad baja de neutrófilos le pone al paciente en riesgo de infección. Factores estimulantes de Colonias (FECs) son medicamentos que se administran a veces a los pacientes que están recibiendo tratamiento de cáncer para prevenir que les toque o tratar a pacientes que tienen neutropenia febril. FECs se inyectan bajo la piel o en una vena.

Al menudo las guías indican que los médicos deben de basar su administración de FECs durante el tratamiento de cáncer según la probabilidad de que esos fármacos aumentarían el riesgo de neutropenia febril. Investigaciones han mostrado que muchos médicos no siguen estas directrices. Esto puede resultar dañino a pacientes. El uso bajo de FECs puede aumentar el riesgoque un paciente tenga de neutropenia febril. El uso excesivo o innecesario de FECs puede conducir a efectos secundarios como dolores óseos y musculares, y resultar de alto costo al paciente sin darle ningún beneficio.

En unas clínicas hay un sistema automatizado que ayuda los médicos a decidir cuándo utilizar los FECs. El sistema receta los FECs cuando existe alto riesgo que los fármacos causarán neutropenia febril. No receta los FECs cuando hay bajo riesgo que los fármacos causarán neutropenia febril. El equipo de investigación del estudio quiere saber si este tipo de sistema puede ayudar a los médicos usar los FECs cuando sea necesario y evitar el uso cuando no se necesita. El equipo del estudio también quiere aprender acerca de los beneficios y riesgos de lautilización de FECs con fármacos de tratamiento de cáncer que tienen un riesgo moderado (ni alto ni bajo) de neutropenia febril.

Se le pide a usted que tome parte en este estudio porque estará recibiendo su tratamiento de cáncer en una clínica que ha elegido participar en este estudio. Clínicas que eligen participar eneste estudio se asignan a uno de los cuatro grupos siguientes:

Grupo Clinical #1: Clínicas que ya tienen un sistema automatizado que ayuda a los médicos en decidir cuándo se deben de usar los FECs serán asignados al Grupo #1. Estossistemas recetan los FECs cuando existe alto riesgo que los fármacos de tratamiento de cáncer causarán neutropenia febril. No recetan los FECs cuando hay bajo riesgo de que los fármacos causarán neutropenia febril. El estudio no les pedirá que las clínicas cambian lo que normalmente hacen. Estas clínicas usarán su sistema para decidir cuándousar los FECs. Para pacientes en estas clínicas, el cuidado no diferenciará a lo que recibirán fuera del estudio. Hay ocho clínicas participando en el Grupo #1 del estudio. (La inscripción al Grupo Clínico #1 está cerrada definitivamente a partir del 2 de Diciembre, 2019.)

Clínicas que no tienen un sistema automatizado serán asignados a Grupo Clinical o #2, o #3, o #4. Una computadora asignará a cada clínica a un grupo de manera al azar. Esto se llama aleatorización. Asignación se hace de forma aleatoria porque para que nadie sabe si uno grupo esmejor o peor que los demás.

Grupo Clinical #2: En las clínicas asignadas al Grupo #2 no hay sistemas automatizadosinstalados. El estudio no les pedirá que las clínicas cambian lo que hacen usualmente.
Para pacientes en estas clínicas, el cuidado no diferenciará a lo que recibirán fuera delestudio. Hay ocho clínicas participando en el Grupo #2 del estudio

Grupo Clinical #3: Clínicas asignadas al Grupo #3 no tienen sistemas automatizados pero se instalarán sistemas automatizados como parte del estudio. El sistema se les sugerirá que los FECs sean recetados por los fármacos que tienen riesgo alto o moderadode neutropenia febril. La diferencia entre el Grupo #3 y Grupo #4 es que para clínicas delGrupo #3 el sistema les sugerirá que el FEC sea utilizado para regímenes que tienen riesgo moderado de neutropenia febril. El sistema que no se utilice FECs para los fármacos que tienen bajo riesgo de neutropenia febril. Si el médico no está de acuerdo con la sugerencia él/ella no la tiene que seguir. Para pacientes de estas clínicas, el tratamiento no será diferente a lo que recibiría fuera del estudio. Sin embargo, pueda ser que el estudio afecta si un paciente recibe FECs. Hay doce clínicas participando en el estudio del Grupo #3.

Grupo Clinical #4: Clínicas asignadas al Grupo #4 no tienen sistemas automatizados.Pero uno se instalará como parte del estudio. El sistema sugerirá que los FECs sean recetados por los fármacos que tienen riesgo alto de neutropenia febril. El sistema sugerirá que los FECs no se utilizan para fármacos de bajo o moderado riesgo de neutropenia febril. La diferencia Grupo #3 y Grupo #4 es que para las clínicas en el Grupo #4 el sistema que *no* se utiliza los FECs para los fármacos que tienen riesgo

moderado de neutropenia febril. Si el médico no está de acuerdo con la sugerencia él/ellano la tiene que seguir. Para pacientes de estas clínicas, el tratamiento no será diferente a lo que recibiría fuera del estudio. Sin embargo, pueda ser que el estudio afecta si un paciente recibe FECs. Hay doce clínicas participando en el estudio del Grupo #4.

Su clínica ha sido asignado al Grupo #__________.

Habrán aproximadamente 3,600 personas tomando parte en este estudio.

¿Qué es el enfoque usual del uso de información médica para investigaciones?

Si usted está de acuerdo ser parte del estudio, el equipo del estudio revisará sus archivos médicosy sus respuestas a los cuestionarios para responder a preguntas de la investigación

Hospitales y consultorios médicos usualmente utilizan un formulario llamado "Divulgación deInformación Médica" para conseguir información médica de pacientes. Para este estudio, los investigadores están utilizando un formulario de consentimiento que describe cuales tipos de información les gustaría obtener de sus archivos médicos. El formulario de consentimiento le pide a usted su permiso para conseguir esta información de su historial médico junto con sus respuestas a los cuestionarios de investigación.

¿Cuáles son mis otras opciones si decido no tomar parte en este estudio?

Su decisión de participar (o no) en este estudio de investigación NO afectará su tratamiento decáncer. Si decide no tomar parte en este estudio de investigación, tiene otras elecciones. Por ejemplo:

- Puede recibir tratamiento por su cáncer sin participar en un estudio
- Pueda elegir tomar parte en otro estudio, si hay uno disponible

¿Por cuánto tiempo permaneceré en el estudio?

Estará en el estudio por 12 meses.

¿De qué consta?

Si decide tomar parte en el estudio, información de sus archivos médicos se obtendrán en lostiempos siguientes:

- Al tiempo que se una con el estudio (basal)
- Después del primer ciclo de tratamiento
- 1 mes después de que se una al estudio
- 6 meses después de unirse al estudio

- 12 meses después de unirse al estudio

Los investigadores obtendrán la siguiente información de sus archivos médicos:

- Información básica de usted (p.ej., género, estatura, peso)
- Tipo de cáncer
- Su historial médico, problemas médicos actuales, estado de salud
- Nombres y dosis de fármacos por el tratamiento de su cáncer (planeado y recibido/actual)
- Nombres y dosis de los FECs recibidos
- Nombres de otros fármacos recibidos durante tratamiento
- Ocurrencia de neutropenia febril (incluso visitas al hospital y/o urgencias)
- Efectos secundarios
- Resultados de pruebas de sangre

También se le pedirá que responda a cuestionarios en los tiempos siguientes:

- Al tiempo que usted se une con el estudio (basal)
- Después del primer ciclo de tratamiento
- 6 meses después de unirse al estudio

Los investigadores obtendrán la información siguiente, directamente de usted por medio de suscuestionarios:

- Información acerca de usted (nivel de educación, ingresos)
- Problemas físicos no relacionados a cáncer
- Información de gastos no re-embolsados de su tratamiento
- Lo que usted sabe de los fármacos FECs y de la neutropenia febril
- Información acerca de dónde y cómo recibió los FECs y antibióticos
- Información acerca de su bienestar físico, emocional y social y a qué nivel usted estáfuncionando

A usted le darán hasta tres cuestionarios en cada punto de tiempo: tres al tiempo basal, dos al terminar su primer ciclo de tratamiento y uno a los 6 meses. Le puede tomar entre 15-20 minutos para responder a los cuestionarios (cada vez que conteste uno). Los investigadores preferirían que usted rellene los cuestionarios durante su visita a la clínica, pero también puede rellenar los cuestionarios en casa o por medio del teléfono. Si rellene los cuestionarios en casa los puede devolver por correo en un sobre con franqueo pre-pagado y pre-impreso con la dirección del estudio, o traerlos consígo a la próxima cita en la clínica. El personal de la investigacióntrabajará con usted para determinar cuál opción sea lo más fácil para sí.

¿Cuáles otras pruebas y procedimientos me tocarán se tomo parte en este estudio?

No se necesita pruebas ni procedimientos extras para participar en este estudio.

¿Cuáles son los posibles riesgos de tomar parte en este estudio?

Si opte tomar parte en este estudio, hay un riesgo de que se siente incómodo que le preguntan acerca de su bienestar funcional, físico, emocional y social.

El equipo del estudio ha implementado pólizas para proteger sus datos personales y harán todo loposible para asegurar que se guarde la privacidad de ellos. Aun así, no le puede garantizar privacidad total. Pueda ser que se divulga datos personales suyos si se lo requiere la ley. Si información del estudio se publica o se presenta en reuniones científicas el nombre suyo junto con sus otros datos personales **no** se utilizarán.

Para ayudar a mantener la privacidad de su información, su médico o enfermera la enviará a otros investigadores por medio de un portal seguro de presentación de datos patrocinado por elInstituto Nacional del Cáncer (NCI por sus siglas en inglés). Luego el equipo del estudio puederecurrir al mismo portal seguro para recuperar su información e incluirla con la de todos los pacientes demás que están tomando parte en el estudio.

¿Cuáles son los posibles beneficios de tomar parte en este estudio?

La información que provee pueda ayudar a que los investigadores mejoran su conocimiento la manera en que los médicos y clínicas recetan los FECs y si la forma en que los recetan sea eficazal reducir la neutropenia febril.

¿Puedo dejar de participar en el estudio?

Sí. Usted puede dejarse de participar en cualquier momento. Si decide dejar de participar por cualquier razón, es importante que deje saber al médico del estudio tan pronto que sea posible. Sidecide parar su participación, puede decidir si quiere permitir o no que el médico del estudio sigaproviendo su información médica a la organización que está conduciendo el estudio.

¿Cuáles son mis derechos en este estudio?

Tomarse parte en este estudio es una elección suya. No importa la decisión que haga y aunque sudecisión cambie, no habrá ninguna pena a usted. No perderá cuidado médico ni derechos legales.

Para preguntas de sus derechos mientras participe en este estudio, por favor llameal____*(insertar nombre del centro)*
Consejo de Revisión Institucional (CRI) al______________________________*(insertar número telefónico). (Nota al Investigador Local: Información de contacto de representativos de pacientes y otros individuos u otros individuos en la institución local quienes no son del CRI/Consejo de RevisiónInstitucional ni el equipo del estudio pero que sí reciben llamadas relacionadas a preguntas acerca de ensayos clínicos también se pueden anotar aquí).*

¿Cuáles son los costos de participar en este estudio?

No hay ningún costo de participar en el estudio. Los del equipo del estudio organizarán sus investigaciones alrededor de las otras citas médicas que usted tiene para que no tenga que hacerviajes separados a la clínica para este estudio. Personal de la investigación le proveerán sobres con franqueo si elige devolver los cuestionarios por medio del correo.

Usted y/o su plan de salud/compañía de seguro necesitará(n) pagar por todos los costos demás detratar su cáncer durante el tiempo que permanezca en el estudio, incluso los costos de pruebas, procedimientos o fármacos para manejar efectos secundarios alguno, a menos que le digan que ciertas pruebas serán provistas sin costo. Antes que decida participar en el estudio debe de averiguar con su plan de salud o compañía de seguro para enterarse precisamente de lo que pagan.

¿Qué sucede si me lesiono o estoy herido en este estudio?

Existe poco riesgo de que sea lesionado en este estudio cada que los del equipo de investigaciónsólo están recolectando información de sus archivos médicos y de cuestionarios.

¿Quiénes verán mi información médica?

Su privacidad es muy importante a los investigadores y harán todos los esfuerzos posibles para protegerla. Los médicos del estudio tienen una licencia de privicidad para ayudar a proteger su registro si hay un proceso judicial. Su información se puede divulgar si se lo requiere la ley. Por ejemplo, ciertos estados requieren que médicos informen a Consejerías de Sanidad si encuentran una enfermedad como tuberculosis. No obstante, los investigadores harán lo posible para asegurar que la información que divulgan no le identificará a usted. Unos datos suyos de salud deeste estudio se mantendrán en un base de datos central para investigación. No se pondrán su nombre ni información de contacto en la base de datos.

Hay organizaciones que pueden inspeccionar sus archivos. A estas organizaciones, se requiere que aseguran que mantienen su información privada, a menos que la ley requiere que se divulgainformación. Algunas de las organizaciones son:

- El patrocinador, SWOG.
- Consejo de Revisión Institucional (CRI), (IRB por sus siglas en inglés), es un grupo depersonas que revisan la investigación con la meta de proteger a las personas que participan en el estudio.
- Instituto Nacional del Cáncer (NCI por sus siglas en inglés)
- El Instituto Hutchinson para Investigaciones de los Resultados de Cáncer (HICOR porsus siglas en inglés)

[Nota a Investigadores Locales: El NCI ha recomendado que los reglamentos de HIPAA sean dirigidos por la institución local. Los reglamentos puedan ser incluidos o no en el formulario deconsentimiento informado según la póliza de la institución local.]

¿Dónde puedo conseguir más información?

Vea al sitio web del NCI al http://cancer.gov/espanol para más información de estudios o información general del cáncer. También puede llamar el Servicio Informativo de Cáncer delNCI al: 1-800-4-CANCER (1-800-422-6237).

Una descripción de este ensayo clínico estará disponible al: http://www.ClinicalTrials.gov, como se requiere la ley de los Estados Unidos. Este sitio web no incluirá información que leidentificará a usted. A lo más, el sitio web incluirá un resumen de los resultados. Puede buscar el sitio web en cualquier momento.

¿Quién podría contestar preguntas que tenga de este estudio?

Usted puede conversar con un médico del estudio de cualquier pregunta o preocupación que tenga del estudio o para informarle de efectos secundarios o lesiones. Contáctelo al médico delestudio al___*(insertar nombre(s) del médico/de los médicos del estudio)* al
______________________ *(insertar número telefónico).*

Contacto Futuro

A veces los investigadores que trabajan con SWOG tienen otras ideas de investigación que relacionan a gente que fueron parte de un estudio de SWOG. En unos casos, para llevar a cabo investigación, investigadores necesitan contactar a los participantes en algún estudio particular.Usted puede estar de acuerdo o no a contacto futuro al poner un círculo a “sí o no”

Estoy de acuerdo en permitir que el médico del estudio o alguien aprobado porél/ella se comunique conmigo de investigación(es) futura(s) referente a mi participación en este estudio.

SÍ **NO**

Mi firma que muestra que estoy de acuerdo en participar en este estudio

He leído, o alguien me ha leído, este formulario de consentimiento. El médico del estudio y yo hemos hablado y ha contestado mis preguntas. Me dará una copia del formulario firmado. Estoyde acuerdo tomar parte en este estudio.

Firma del participante (o representante legal) firma____

Fecha de firma____

Firma del investigador que está obteniendo consentimiento _________________________

Fecha de firma ____
