# Supplementary material for: Effects of a Guideline-Informed Clinical Decision Support System Intervention to Improve Colony-Stimulating Factor Prescribing: A Cluster Randomized Clinical Trial
Source: JAMA Netw Open. 2022 Oct 24;5(10):e2238191. doi: 10.1001/jamanetworkopen.2022.38191 (PMC9593234; doi:10.1001/jamanetworkopen.2022.38191)
Supplement: Supplement 2. — eTable 1. PP-CSF Use: Comparison of Mixed and GEE Models: High and Low-Risk Patients eTable 2. Temporal Trends in PP-CSF Use, Assessed Using Registration Year in High-Risk and Low-Risk Patients eTable 3. CSF Use by Age (≤65 Years vs >65 Years) in (a) High-Risk and (b) Low-Risk Patients eTable 4. Febrile Neutropenia: Comparison of Mixed and GEE Models: High and Low-Risk Patients eTable 5. Febrile Neutropenia by Chemotherapy Risk Group, Conditional on CSF Prescription eTable 6. Febrile Neutropenia by Chemotherapy Risk Group, Conditional on No CSF Prescription [file jamanetwopen-e2238191-s002.pdf]

Supplemental Online Content

Ramsey SD, Bansal A, Sullivan SD, et al. Effects of a guideline-informed clinical decision support system intervention to improve colony-stimulating factor prescribing: a cluster randomized clinical trial. *JAMA Netw Open*. 2022;5(10):e2238191. doi:10.1001/jamanetworkopen.2022.38191

eTable 1. PP-CSF Use: Comparison of Mixed and GEE Models: High and Low-Risk Patients
eTable 2. Temporal Trends in PP-CSF Use, Assessed Using Registration Year in High-Risk and Low-Risk Patients
eTable 3. CSF Use by Age (≤65 Years vs >65 Years) in (a) High-Risk and (b) Low-Risk Patients
eTable 4. Febrile Neutropenia: Comparison of Mixed and GEE Models: High and Low-Risk Patients
eTable 5. Febrile Neutropenia by Chemotherapy Risk Group, Conditional on CSF Prescription
eTable 6. Febrile Neutropenia by Chemotherapy Risk Group, Conditional on No CSF Prescription

This supplemental material has been provided by the authors to give readers additional information about their work.

© 2022 Ramsey SD et al. *JAMA Network Open.*

eTable 1. PP-CSF Use: Comparison of Mixed and GEE Models:

High and Low-Risk Patients

Model of CSF use among high-risk[a] patients

	Mixed Model		GEE Population-Average Model	
	Odds Ratio (95% CI)	p-value	Odds Ratio (95% CI)	p-value
**Intervention vs. Usual Care**	0.44 (0.12-1.57)	P=0.21	0.46 (0.18-1.19)	P=0.11
**Random effect variance**	1.536	(p<0.001)	NA	NA
**Intraclass correlation (exch)**	NA	NA	0.140	NA
**Age group**	NA	P=0.59	NA	P=0.51
**Age < 50**	1.0 (ref)	NA	1.0 (ref)	NA
**Age 50-59**	0.84 (0.48-1.48)	NA	0.87 (0.57-1.33)	NA
**Age 60-69**	0.70 (0.40-1.22)	NA	0.74 (0.50-1.11)	NA
**Age 70+**	1.01 (0.46-2.21)	NA	1.01 (0.59-1.72)	NA
**Male**	NA[b]	NA[b]	NA[b]	NA[b]
**Cancer type**	NA	NA	NA	NA
**Breast**	All high-risk patients	NA	All high-risk patients	NA
**Colorectal**	NA	NA	NA	NA
**NSCLC**	NA	NA	NA	NA
**Comorbidity**	NA	P=0.23	NA	P=0.16
**> 0 vs 0**	0.76 (0.48-1.19)	NA	0.80 (0.58-1.09)	NA
**Race**	NA	P=0.09	NA	P=0.08
**Asian**	0.32 (0.10-0.97)	NA	0.41 (0.17-0.98)	NA
**Black**	1.78 (0.85-3.74)	NA	1.66 (0.73-3.81)	NA
**White**	1.0 (ref)	NA	1.0 (ref)	NA
**Other/mixed[d]**	0.59 (0.19-1.81)	NA	0.65 (0.30-1.41)	NA
**Unknown**	0.81 (0.25-2.58)	NA	0.86 (0.39-1.93)	NA
**Hispanic ethnicity**	NA	P=0.89	NA	P=0.46
**Hispanic**	1.07 (0.49-2.32)	NA	1.00 (0.60-1.67)	NA
**Unknown**	0.70 (0.14-3.56)	NA	0.78 (0.52-1.17)	NA
**White**	1.0 (ref)	NA	1.0 (ref)	NA

Abbreviations: CI, Confidence Interval; CSF, Colony Stimulating Factor; GEE, Generalized Estimating Equation; ICC, Intraclass Correlation Coefficient; NA, Not Applicable; NSCLC, Non-small Cell Lung Cancer

[a]High-risk = >20% risk of febrile neutropenia, low-risk = <10% risk of febrile neutropenia

[b] Sex not modeled due to only 13 men with breast cancer

[c] Cancer type, comorbidity, race, Hispanic ethnicity not modeled due to small number of events

[d] "Other" includes Native American, Pacific Islander, and Multiple Races

© 2022 Ramsey SD et al. *JAMA Network Open.*

Model of CSF use among low-risk[a] patients

	Mixed Model		GEE Population-Average Model	
	Odds Ratio (95% CI)	p-value	Odds Ratio (95% CI)	p-value
**Intervention vs. Usual Care**	1.18 (0.44-3.20)	P=0.74	1.12 (0.43-2.90)	P=0.74
**Random effect variance**	0.125	(p=0.29)	NA	NA
**Intraclass correlation (exch)**	NA	NA	0.008	NA
**Age group**	NA	P=0.11	NA	P=0.11
**Age < 50**	1.0 (ref)	NA	1.0 (ref)	NA
**Age 50-59**	0.40 (0.06-2.73)	NA	0.39 (0.06-2.61)	NA
**Age 60-69**	0.78 (0.15-4.11)	NA	0.76 (0.20-2.88)	NA
**Age 70+**	1.58 (0.32-7.88)	NA	1.56 (0.41-5.91)	NA
**Male**	0.43 (0.19-0.95)	P=0.04	0.43 (0.21-0.87)	P=0.04
**Cancer type**	NA	P<0.001	NA	P<0.001
**Breast**	1.00 (ref)	NA	1.00 (ref)	NA
**Colorectal**	0.50 (0.05-5.06)	NA	0.50 (0.11-2.35)	NA
**NSCLC**	11.35 (3.10-41.5)	NA	11.71 (4.72-29.04)	NA
**Comorbidity**	NA	NA[2]	NA	NA[2]
**> 0 vs 0**	NA[c]	NA	NA[c]	NA
**Race**	NA	NA[2]	NA	NA[2]
**Asian**	NA[c]	NA	NA[c]	NA
**Black**	NA[c]	NA	NA[c]	NA
**White**	NA[c]	NA	NA[c]	NA
**Other/mixed[d]**	NA[c]	NA	NA[c]	NA
**Unknown**	NA[c]	NA	NA[c]	NA
**Hispanic ethnicity**	NA	NA[2]	NA	NA[2]
**Hispanic**	NA[c]	NA	NA[c]	NA
**Unknown**	NA[c]	NA	NA[c]	NA
**White**	NA[c]	NA	NA[c]	NA

Abbreviations: CI, Confidence Interval; CSF, Colony Stimulating Factor; GEE, Generalized Estimating Equation; ICC, Intraclass Correlation Coefficient; NA, Not Applicable; NSCLC, Non-small Cell Lung Cancer

[a]High-risk = >20% risk of febrile neutropenia, low-risk = <10% risk of febrile neutropenia

[b] Sex not modeled due to only 13 men with breast cancer

[c] Cancer type, comorbidity, race, Hispanic ethnicity not modeled due to small number of events

[d] "Other" includes Native American, Pacific Islander, and Multiple Races

© 2022 Ramsey SD et al. *JAMA Network Open.*

eTable 2. Temporal Trends in PP-CSF Use, Assessed Using Registration Year in High-Risk and Low-Risk Patients

Registration Year	Usual Care	Intervention
**High FN-risk**		
Denominator	128	458
All (n=1259)	95.8%	89.2%
2016-2017 (n=304)	95.8%	90.0%
2018 (n=446)	95.3%	86.4%
2019-2020 (n=509)	96.3%	91.0%
**Low FN-risk**		
Denominator	309	949
All (n=586)	5.5%	6.3%
2016-2017 (n=188)	8.0%	2.9%
2018 (n=172)	5.1%	7.5%
2019-2020 (n=226)	2.6%	8.0%

Abbreviations: FN, Febrile Neutropenia; PP-CSF, Primary Prophylactic Colony Stimulating Factor

© 2022 Ramsey SD et al. *JAMA Network Open.*

eTable 3. CSF Use by Age (≤65 Years vs >65 Years) in (a) High-Risk and (b) Low-Risk Patients

	Usual Care	Intervention
**High FN-risk**		
Denominator	309	949
Overall (n=1259)	95.8%	89.2%
<= 65 (n=1000)	96.7%	89.1%
> 65 (n=259)	92.2%	89.2%
**Low FN-risk**		
Denominator	128	458
Overall (n=586)	5.5%	6.3%
<= 65 (n=333	1.4%	3.9%
> 65 (n=253)	11.1%	9.5%

Abbreviations: CSF, Colony Stimulating Factor; FN, Febrile Neutropenia

© 2022 Ramsey SD et al. *JAMA Network Open.*

eTable 4. Febrile Neutropenia: Comparison of Mixed and GEE Models:

High and Low-Risk Patients

Model of FN among high-risk[a] patients

	Mixed Model		GEE Population-Average Model	
	Odds Ratio (95% CI)	p-value	Odds Ratio (95% CI)	p-value
**Intervention vs. Usual Care**	1.49 (0.75-2.95)	P=0.26	1.48 (0.75-2.90)	P=0.26
**Random effect variance**	0.109	0.17	NA	NA
**Intraclass correlation (exch)**	NA	NA	0.003	NA
**Age group**	NA	P=0.29	NA	P=0.27
**Age < 50**	1.0 (ref)	NA	1.0 (ref)	NA
**Age 50-59**	1.29 (0.68-2.47)	NA	1.29 (0.81-2.05)	NA
**Age 60-69**	1.40 (0.72-2.69)	NA	1.40 (0.75-2.61)	NA
**Age 70+**	2.05 (0.98-4.29)	NA	2.08 (0.99-4.37)	NA

Abbreviations: CI, Confidence Interval; FN, Febile Neutopenia; GEE, Generalized Estimating Equation; ICC, Intraclass Correlation Coefficient; NA, Not Applicable;
[a]High-risk = >20% risk of febrile neutropenia, low-risk = <10% risk of febrile neutropenia
Cancer type, comorbidity, race, Hispanic ethnicity not modeled due to small number of events

Model of FN among low-risk[a] patients

	Mixed Model		GEE Population-Average Model	
	Odds Ratio (95% CI)	p-value	Odds Ratio (95% CI)	p-value
**Intervention vs. Usual Care**	2.09 (0.23-18.80)	P=0.51	1.97 (0.27-14.25)	P=0.50
**Random effect variance**	0.417	0.34	NA	NA
**Intraclass correlation (exch)**	NA	NA	0.000	NA
**Cancer type**	NA	P=0.39	NA	P=0.41
**Breast**	1.0 (ref)	NA	1.0 (ref)	NA
**Colorectal**	4.57 (0.49-42.80)	NA	3.83 (0.53-27.84)	NA
**NSCLC**	2.68 (0.27-26.92)	NA	2.00 (0.28-14.53)	NA

Abbreviations: CI, Confidence Interval; FN, Febile Neutopenia; GEE, Generalized Estimating Equation; ICC, Intraclass Correlation Coefficient; NA, Not Applicable; NSCLC, Non-small Cell Lung Cancer
[a]High-risk = >20% risk of febrile neutropenia, low-risk = <10% risk of febrile neutropenia
Age, biologic sex, comorbidity, race, Hispanic ethnicity not modeled due to small number of events

© 2022 Ramsey SD et al. *JAMA Network Open.*

eTable 5. Febrile Neutropenia by Chemotherapy Risk Group, Conditional on CSF Prescription

	Randomized Usual Care	All Randomized	Cohort
**High FN Risk**			
Denominator	296	1141	357
**Observed FN**	**11 (3.7%)**	**54 (4.7%)**	**19 (5.3%)**
**Low FN Risk**			
Denominator	7	34	10
**Observed FN**	**0 (0.0%)**	**0 (0.0%)**	**0 (0.0%)**

Abbreviations: CSF, Colony Stimulating Factor; FN, Febrile Neutropenia; NA, Not Applicable

© 2022 Ramsey SD et al. *JAMA Network Open.*

eTable 6. Febrile Neutropenia by Chemotherapy Risk Group, Conditional on No CSF Prescription

	**Randomized Usual Care**	**All Randomized**	**Cohort**
**High FN Risk**			
Denominator	12	114	27
**Observed FN**	**2 (16.7%)**	**17 (14.9%)**	**6 (22.2%)**
**Low FN Risk**			
Denominator	120	545	111
**Observed FN**	**1 (0.8%)**	**8 (1.5%)**	**2 (1.8%)**

Abbreviations: CSF, Colony Stimulating Factor; FN, Febrile Neutropenia

© 2022 Ramsey SD et al. *JAMA Network Open.*
