# Supplementary material for: Effects of a Guideline-Informed Clinical Decision Support System Intervention to Improve Colony-Stimulating Factor Prescribing: A Cluster Randomized Clinical Trial
Source: JAMA Netw Open. 2022 Oct 24;5(10):e2238191. doi: 10.1001/jamanetworkopen.2022.38191 (PMC9593234; doi:10.1001/jamanetworkopen.2022.38191)
Supplement: Supplement 3. — Data Sharing Statement [file jamanetwopen-e2238191-s003.pdf]

Data Sharing Statement

Ramsey. Effects of a Guideline-Informed Clinical Decision Support System Intervention to Improve Colony-Stimulating Factor Prescribing. *JAMA Netw Open*. Published October 24, 2022. doi:10.1001/jamanetworkopen.2022.38191

Data
Data available: Yes
Data types: Deidentified participant data, Data dictionary
How to access data: National Clinical Trials Network (NCTN) Data Archives
When available: With publication

Supporting Documents
Document types: None

Additional Information
Who can access the data: The National Cancer Institute's (NCI) NCTN/NCORP Data Archives is a centralized, controlled-access database for sharing datasets generated from clinical trials of the National Clinical Trials Network (NCTN) and the NCI Community Oncology Research Program (NCORP). These datasets are made available on appropriate terms and conditions to researchers who wish to analyze the data in secondary studies to enhance the public health benefit of the original work.
Types of analyses: Secondary studies to enhance the public health benefit of the original work.
Mechanisms of data availability: Requirements may include but are not limited to: a research plan, a data use agreement (DUA) and legally binding signatures.
